# Supplementary material for: Representativeness in population-based studies of older adults: five waves of cross-sectional examinations in the Gothenburg H70 Birth Cohort Study
Source: BMJ Open. 2022 Dec 16;12(12):e068165. doi: 10.1136/bmjopen-2022-068165 (PMC9764666; doi:10.1136/bmjopen-2022-068165)
Supplement: Supplementary data [file bmjopen-2022-068165supp001.pdf]

Additional file 1

BMJ Open

Representativeness in population-based studies of older adults – Five waves of cross-sectional examinations in the Gothenburg H70 Birth Cohort Study

Hanna Wetterberg, MSc[ab]*, Lina Rydén, MD[abc]*, Felicia Ahlner, MSc[ab], Hanna Falk Erhag, RN, PhD[ab], Pia Gudmundsson, PhD[ab], Xinxin Guo, MD, PhD[ab],[d], Erik Joas, PhD[ab], Lena Johansson, RN, PhD[ab], Silke Kern, MD, PhD[abc], Madeleine Mellqvist Fässberg, PhD[ab], Jenna Najar, MD, PhD[abc], Mats Ribbe, MD, PhD[ab], Therese Rydberg Sterner, PhD[abe], Jessica Samuelsson, MSc[ab], Simona Sacuiu MD, PhD[abc], Robert Sigström, MD, PhD[abc], Johan Skoog, PhD[ab], Margda Waern, MD, PhD[abf], Anna Zettergren, PhD[ab], Ingmar Skoog, MD, PhD[abc]

[*] HW and LR are joint first authors

[a]*Neuropsychiatric Epidemiology Unit, Department of Psychiatry and Neurochemistry, Institute of Neuroscience and Physiology, Sahlgrenska Academy, at the University of Gothenburg, Sweden;* [b]*Centre for Ageing and Health (AGECAP) at the University of Gothenburg, Sweden;* [c]*Region Västra Götaland, Sahlgrenska University Hospital, Psychiatry, Cognition and Old Age Psychiatry Clinic, Gothenburg, Sweden;* [d]*Region Västra Götaland, Sahlgrenska University Hospital, Department of Mood disorders, Gothenburg, Sweden;* [e]*Aging Research Center, Department of Neurobiology, Care Sciences and Society, Karolinska Institutet and Stockholm University, Stockholm, Sweden;* [f]*Region Västra Götaland, Department of Psychiatry, Psychotic Disorders, Sahlgrenska University Hospital, Mölndal, Sweden*

Corresponding author:

Hanna Wetterberg, hanna.wetterberg@gu.se

Table s1*. *Examinations included at each wave

	2000	2005	2009	2015	2018
General examinations					
General health interview	YES	YES	YES	YES	YES
Psychiatric interview	YES	YES	YES	YES	YES
Cognitive examination[a]	YES [g]	YES	YES	YES	YES
Physical examination by medical doctor	YES	YES	NO	NO	NO
Physical examination[b]	YES	YES	YES	YES	YES
Social factors interview	YES	YES	YES	YES	YES
Physiotherapy examination	YES	YES	YES	YES	YES
Functional ability[d]	YES	YES	YES	YES	YES
Periodic limb movement	YES[h]	YES	NO	NO	NO
Audiological examination	YES	YES	NO	YES[i]	NO
Dental examination	YES	YES	NO	NO	NO
Blood sampling[e]	YES	YES	YES	YES	YES
Genetic analyses	YES	YES	YES	YES	YES
Urine sampling	YES	YES	NO	NO	NO
Personality tests (Cesarec Marke Personality Scheme and Eysenck)	NO	YES	YES	YES	NO
Additional examinations					
Close informant interview	YES	YES	YES	YES	YES
Dietary examination	YES	NO	NO	YES	NO
Body composition examination					
Dual-energy X-ray absorptiometry	NO	YES[h]	YES[h]	YES[h]	YES[h]
Bioelectrical impedance spectroscopy	NO	YES[h]	YES[h]	YES[h]	YES[h]
Brain imaging examination					
Magnetic resonance imaging	NO	NO	NO	YES	YES
Computed tomography	YES	NO	YES	YES	YES
Cerebrospinal fluid sampling[f]	NO	NO	YES	YES	YES
Extended audiological examination	NO	NO	NO	YES[h]	YES[h]

[a] Including clinical screening tests as well as more extensive psychometric tests
[b] Including examinations such as blood pressure, anthropometry, visual acuity, neurological status, electrocardiogram, lung function examinations, Bioelectrical Impedance Analysis,
[d] Measured as Activities of Daily Living (ADL), and instrumental activities of daily living (IADL) similar to Katz and Lawton ADL, and from 2015 also the Barthel index.
[e] Used for e.g. clinical chemistry analyses.
[f] Collected by lumbar puncture.
[g] Half of the sample performed an extended psychometric test, which in later cohorts were performed by all participants.
[h] A subsample was invited.
[i] Performed on those examined at the research clinic and a subsample of those examined at home.

Table s2. The hospital discharge diagnoses included

	ICD 9	ICD 10
Cancer	140-208	C00-C97
Neuropsychiatric disorders including dementia disorders	290-319, 324-359<br>290, 291C, 294, 331	F01-F99, G06-G98<br>F01, F02, G30, G31]
Cardiovascular diseases	390-459	I00-I99
Diabetes mellitus	250	E10-E14
Unipolar depression	296B, 311	F32-F33
Alcohol-related disorders	291, 303, 305A	F10
Ischemic heart disease	410-414	I20-I25
Cerebrovascular diseases	430-438	I60-I69
Chronic obstructive pulmonary disease	490-492, 495-496	J40-J44
Osteoarthritis	715	M15-M19

Table s3. Comparison between examinations of excluded individuals

**Total excluded (includes did not speak Swedish, died after sampling, and not traceable)**					
	**2000** 2.8% (22/775)	**2005** 4.3% (54/1250)	**2009** 10.0% (106/1056)	**2015** 12.0% (92/764)	**2018** 9.0% (50/555)
**2000**, 2.8% (22/775)	-	p=0.088	**p<0.001**	**p<0.001**	**p<0.001**
**2005**, 4.3% (54/1250)		-	**p<0.001**	**p<0.001**	**p<0.001**
**2009**, 10.0% (106/1056)			-	p=0.175	p=0.507
**2015**, 12.0% (92/764)				-	p=0.079
**2018**, 9.0% (50/555)					-

**Did not speak Swedish**					
	**2000** 1.6% (12/775)	**2005** 1.9% (24/1250)	**2009** 3.7% (39/1056)	**2015** 4.1% (31/764)	**2018** 1.6% (9/555)
**2000,**1.6% (12/775)	**-**	p=0.538	**p=0.006**	**p=0.003**	p=0.916
**2005,** 1.9% (24/1250)		-	**p=0.009**	**p=0.001**	p=0.867
**2009,** 3.7% (39/1056)			-	p=0.690	**p=0.020**
**2015,** 4.1% (31/764)				-	**p=0.011**
**2018,** 1.6% (9/555)					**-**

**Died after sampling**					
	**2000** 0.7% (5/775)	**2005** 0.9% (11/1250)	**2009** 3.1% (33/1056)	**2015** 5.5% (42/764)	**2018** 3.8% (21/555)
**2000,** 0.7% (5/775)	-	p=0.562	**p<0.001**	**p<0.001**	**p<0.001**
**2005,** 0.9% (11/1250)		-	**p<0.001**	**p<0.001**	**p=0.017**
**2009,** 3.1% (33/1056)			-	**p=0.012**	p=0.485
**2015,** 5.5% (42/764)				-	p=0.150
**2018,** 3.8% (21/555)					-

**Not traceable**					
	**2000** 0.5% (4/775)	**2005** 1.4% (17/1250)	**2009** 3.1% (33/1056)	**2015** 0.8% (6/764)	**2018** 3.6% (20/555)
**2000**, 0.5% (4/775)	-	p=0.074	**p<0.001**	p=0.544	**p<0.001**
**2005**, 1.4% (17/1250)		-	**p=0.004**	p=0.324	**p<0.001**
**2009**, 3.1% (33/1056)			-	**p=0.001**	p=0.609
**2015**, 0.8% (6/764)				-	**p<0.001**
**2018**, 3.6% (20/555)					-

Statistical analysis: to test differences between groups χ2 or Fisher's exact test were used. Bolded numbers are significant at p <0.05.

Table s4. Characteristics of 70 years old participants and refusals, and same aged individuals in Gothenburg and Sweden in 2000, stratified by sex

	**Women**				**Men**			
	**Participants**	**Refusals**	**Gothenburg**	**Sweden**	**Participants**	**Refusals**	**Gothenburg**	**Sweden**
N	281 (53.6)	109 (47.6)	1 965 (56.0)	40 655 (53.9)	243[a] (46.4)	120 (52.4)	1 543 (44.0)	34 813 (46.1)
**Sociodemographic factors**								
Marital status, % (n)								
Married	46.3 (130)	49.5 (54)	49.0 (963)	**55.2 (22 425)****	74.8 (181)	**56.7 (68)*****	**65.1 (1 005)****	69.7 (24 272)
Widowed	24.9 (70)	22.0 (24)	23.2 (455)	24.4 (9 937)	2.9 (7)	**8.3 (10)***	**6.4 (98)***	**6.8 (2 354)***
Divorced	23.1 (65)	19.3 (21)	21.3 (419)	**14.5 (5 899)*****	14.9 (36)	15.0 (18)	16.9 (260)	13.4 (4 677)
Never married	5.7 (16)	9.2 (10)	6.5 (128)	5.9 (2 394)	7.4 (18)	**20.0 (24)*****	11.7 (180)	10.1 (3 510)
Education, % (n)								
Elementary school	54.4 (153)	58.7 (64)	51.5 (1 012)	55.3 (22 497)	41.0 (*100*)	**58.0 (*69*)****	43.2 (666)	**52.3 (18 197)*****
Upper secondary school	31.7 (89)	22.0 (24)	30.1 (591)	29.9 (12 150)	36.4 (88)	32.5 (39)	36.2 (558)	31.2 (10 877)
Higher education	12.1 (34)	15.6 (17)	14.0 (276)	12.5 (5 088)	21.5 (52)	**7.5 (9)*****	17.4 (269)	**14.9 (5 203)****
Born in Sweden, % (n)	83.6 (235)	75.2 (82)	80.0 (1 572)	86.2 (35 049)	83.9 (203)	77.5 (93)	80.9 (1 249)	**89.3 (31 075)****
Income, mean (SD)	8970 (5098)	8270 (6637)	8670 (5190)	8520 (7320)	14370 (10824)	**11980 (8646)***	**12270 (1403)****	**12770 (43821)***
Paid labour, % (n)	3.6 (10)	3.0 (*3*)	2.8 (55)	2.2 (875)	7.0 (17)	5.8 (7)	5.4 (83)	5.7 (1 975)
**Hospital discharge diagnoses,** % (n)								
Cancer	6.8 (19)	9.2 (10)	9.3 (182)	7.6 (3087)	8.2 (20)	10.0 (12)	8.6 (132)	6.9 (2401)
Neuropsychiatric diseases	5.7 (16)	7.3 (8)	9.0 (176)	7.8 (3161)	6.6 (16)	**20.8 (25)*****	**11.5 (177)***	9.2 (3196)
Dementia	1.1 (3)	1.8 (2)	0.6 (11)	0.5 (190)	0.4 (1)	**4.2 (5)***	0.6 (9)	0.5 (167)
Cardiovascular diseases	18.1 (51)	**29.4 (32)***	21.0 (412)	21.3 (8654)	30.0 (73)	40.0 (48)	33.4 (515)	32.9 (11455)
Diabetes mellitus	2.8 (8)	3.7 (4)	3.5 (69)	3.9 (1580)	6.2 (15)	5.8 (7)	6.6 (102)	6.1 (2105)
Unipolar depression	1.1 (3)	0 (0)	1.8 (36)	1.6 (659)	0.4 (1)	0 (0)	1.2 (18)	1.2 (400)
Alcohol-related disorders	0.7 (2)	1.8 (2)	0.8 (15)	0.6 (240)	2.5 (6)	**8.3 (10)***	3.2 (50)	2.3 (816)
Ischemic heart diseases	6.4 (18)	7.3 (8)	7.0 (137)	7.2 (2929)	14.8 (36)	17.5 (21)	17.2 (266)	16.4 (5712)
Cerebrovascular diseases	3.9 (11)	4.6 (5)	4.2 (83)	4.4 (1782)	1.2 (3)	**11.7 (14)*****	**7.0 (108)*****	**7.6 (2640)*****
chronic obstructive pulmonary disease	2.5 (7)	**8.3 (9)****	3.4 (66)	2.0 (808)	2.1 (5)	**6.7 (8)***	3.2 (49)	2.3 (811)

Osteoarthritis	7.8 (22)	*2.8 (3)*	5.3 (104)	6.1 (2479)	4.5 (11)	*1.7 (2)*	4.0 (61)	5.5 (1905)

Comparisons made between participants and refusals, Gothenburg and Sweden.
Statistical analysis: to test differences between groups t test or χ2 test were used. *** p <0.001, ** p <0.01, * p <0.05; bolded numbers are significant at p <0.05; all other values are not significant.
[a] One individual is missing in the sociodemographic variables due personal identity number not found in the register.
Numbers in italics are not exact (true value within a 1-3 range) due to protection of the anonymity.

Table s5. Characteristics of 75 years old participants and refusals, and same aged individuals in Gothenburg and Sweden in 2005, stratified by sex

	Women				Men			
	**Participants**	**Refusals**	**Gothenburg**	**Sweden**	**Participants**	**Refusals**	**Gothenburg**	**Sweden**
N (%)	438 (57.1)	246 (57.3)	1 768 (57.5)	37 075 (55.5)	329 [a] (42.9)	183 (42.7)	1 307 (42.5)	29 780 (44.5)
**Sociodemographic factors**								
Marital status, % (n)								
Married	41.8 (183)	41.9 (103)	42.9 (759)	46.2 (17 134)	71.9 (235)	**55.7 (102)***	**65.2 (852)***	67.5 (20 104)
Widowed	33.1 (145)	26.8 (66)	30.3 (535)	33.9 (12 581)	8.0 (26)	**14.8 (27)***	9.6 (126)	10.5 (3 134)
Divorced	20.1 (88)	25.6 (63)	20.7 (366)	**14.2 (5 282)***	13.1 (43)	16.4 (30)	14.8 (194)	12.7 (3 769)
Never married	5.0 (22)	5.7 (14)	6.1 (108)	5.6 (2 078)	7.0 (23)	**13.1 (24)***	10.3 (135)	9.3 (2 773)
Education, % (n)								
Elementary school	49.0 (*213*)	**59.3 (146)****	51.5 (910)	**54.7 (20 280)***	38.8 (127)	**57.9 (106)***	42.6 (557)	**51.4 (15 304)***
Upper secondary school	35.8 (157)	**23.6 (58)***	**30.3 (535)***	**30.2 (11 190)***	37.0 (121)	**26.2 (48)***	36.0 (470)	**31.4 (9 363)***
Higher education	15.1 (66)	13.0 (32)	14.1 (250)	12.9 (4 772)	22.9 (75)	**12.6 (23)****	18.6 (243)	**15.6 (4 636)***
Born in Sweden, % (n)	83.6 (366)	80.1 (197)	80.4 (1 421)	86.4 (32 025)	81.7 (267)	80.3 (147)	80.6 (1 053)	**89.4 (26 615)***
Income, mean (SD)	10240 (8273)	9920 (7564)	10010 (7157)	9650 (8295)	13680 (7477)	12760 (7789)	13630 (10275)	13410 (24038)
Paid labour, % (n)	1.6 (7)	1.0 (*2*)	1.8 (31)	1.1 (409)	3.7 (12)	2.7 (5)	3.4 (44)	3.4 (1 016)
**Hospital discharge diagnoses,** % (n)								
Cancer	12.1 (53)	11.0 (27)	12.7 (225)	10.4 (3872)	14.6 (48)	14.2 (26)	13.2 (172)	**10.8 (3216)***
Neuropsychiatric diseases	12.3 (54)	14.2 (35)	12.3 (218)	11.2 (4157)	10.6 (35)	**18.6 (34)***	**16.4 (214)****	13.1 (3913)
Dementia	2.1 (9)	2.0 (5)	1.8 (32)	**0.9 (340)***	0.6 (2)	1.6 (3)	1.0 (13)	0.9 (264)
Cardiovascular diseases	32.9 (144)	35.4 (87)	33.5 (592)	32.5 (12042)	42.2 (139)	**53.0 (97)***	47.4 (619)	45.6 (13576)
Diabetes mellitus	8.0 (35)	8.5 (21)	6.3 (111)	6.0 (2238)	9.7 (32)	10.9 (20)	10.6 (139)	8.8 (2634)
Unipolar depression	1.4 (6)	**3.7 (9)***	2.8 (49)	2.4 (883)	1.8 (6)	1.6 (3)	2.5 (33)	1.6 (489)
Alcohol-related disorders	1.1 (5)	0.8 (2)	0.7 (13)	0.6 (230)	1.5 (5)	3.3 (6)	2.9 (38)	2.3 (676)
Ischemic heart diseases	9.6 (42)	11.0 (27)	11.1 (196)	11.1 (4103)	22.5 (74)	23.5 (43)	24.2 (316)	22.7 (6773)

Cerebrovascular diseases	6.4 (28)	6.5 (16)	6.4 (113)	7.1 (2635)	10.6 (35)	10.9 (20)	11.6 (151)	11.5 (3419)
chronic obstructive pulmonary disease	4.3 (19)	6.5 (16)	3.9 (69)	3.0 (1094)	4.0 (13)	6.6 (12)	5.4 (71)	3.9 (1158)
Osteoarthritis	12.1 (53)	**6.1 (15)***	9.5 (167)	10.3 (3834)	6.1 (20)	3.8 (7)	5.7 (74)	8.9 (2643)

Comparisons made between participants and refusals, Gothenburg and Sweden.
Statistical analysis: to test differences between groups t test or χ2 test were used. *** p <0.001, ** p <0.01, * p <0.05; bolded numbers are significant at p <0.05; all other values are not significant.
[a] One individual is missing in the sociodemographic variables due personal identity number not found in the register. Numbers in italics are not exact (true value within a 1-3 range) due to protection of the anonymity.

Table s6. Characteristics of 79 years old participants and refusals, and same aged individuals in Gothenburg and Sweden in 2009, stratified by sex

	Women				Men			
	**Participants**	**Refusals**	**Gothenburg**	**Sweden**	**Participants**	**Refusals**	**Gothenburg**	**Sweden**
N (%)	331 (57.1)	225 (60.8)	1566 (59.2)	33167 (57.0)	249 (42.9)	145 (39.2)	1081 (40.8)	25017 (43.0)
**Sociodemographic factors**								
Marital status, % (n)								
Married	34.1 (113)	37.8 (85)	35.2 (552)	37.7 (12519)	69.9 (174)	**54.5 (79)** **	63.5 (686)	64.8 (16217)
Widowed	40.8 (135)	34.7 (78)	38.4 (602)	43.2 (14325)	12.4 (31)	17.2 (25)	13.5 (146)	14.6 (3648)
Divorced	19.6 (65)	23.1 (52)	20.6 (322)	13.7 (4536)**	9.6 (24)	15.9 (23)	14.0 (151)	12.0 (3014)
Never married	5.4 (18)	4.4 (10)	5.7 (90)	5.4 (1787)	8.0 (20)	12.4 (18)	9.1 (98)	8.5 (2138)
Education, % (n)								
Elementary school	44.0 (*144*)	**63.0 (*142*)*****	**50.1 (784) ***	**54.1 (17955)*****	36.1 (90)	**52.0 (*75*)****	39.0 (422)	**50.4 (12601)*****
Upper secondary school	38.1 (126)	**23.6 (53) *****	**30.6 (479) ****	**30.2 (10033) ****	39.8 (99)	31.7 (46)	37.4 (404)	**31.8 (7953)****
Higher education	17.8 (59)	12.4 (28)	15.2 (238)	**13.3 (4419) ***	24.1 (60)	**14.5 (21)***	20.7 (224)	**16.2 (4056)*****
Born in Sweden, % (n)	83.7 (277)	86.2 (194)	80.4 (1259)	86.4 (28655)	85.1 (212)	86.2 (125)	80.6 (871)	**89.5 (22391)***
Income, mean (SD)	12720 (13490)	**10440 (3880)****	11740 (9393)	**11230 (11699)***	16680 (10368)	14750 (11032)	16150 (15193)	**14780 (19926)****
Paid labour, % (n)	1.0 (*2*)	1.0 (2)	0.6 (10)	0.5 (160)	2.4 (6)	2.0 (*3*)	0.8 (9 [a])*	1.7 (432)
**Hospital discharge diagnoses,** % (n)								
Cancer	16.0 (53)	11.1 (25)	14.7 (230)	12.7 (4217)	15.3 (38)	17.2 (25)	16.5 (178)	14.5 (3634)
Neuropsychiatric diseases	14.2 (47)	16.4 (37)	16.8 (263)	15.6 (5168)	19.3 (48)	13.8 (20)	20.4 (221)	17.7 (4422)
Dementia	2.1 (7)	1.3 (3)	1.8 (28)	1.7 (568)	1.6 (4)	0 (0)	2.0 (22)	1.8 (451)
Cardiovascular diseases	42.9 (142)	37.3 (84)	44.8 (702)	44.9 (14903)	53.0 (132)	53.1 (77)	56.2 (608)	56.5 (14132)
Diabetes mellitus	7.6 (25)	6.2 (14)	8.0 (126)	8.4 (2792)	10.8 (27)	9.0 (13)	11.8 (128)	2903 (11.6)
Unipolar depression	2.7 (9)	4.0 (9)	4.2 (66)	3.3 (1094)	4.4 (11)	2.8 (4)	3.7 (40)	**2.1 (526)***
Alcohol-related disorders	1.2 (4)	0.9 (2)	0.7 (11)	0.6 (189)	0.4 (1)	**5.5 (8)****	**3.1 (34)***	**2.2 (554)***
Ischemic heart diseases	12.4 (41)	9.3 (21)	14.0 (219)	14.5 (4795)	23.7 (59)	29.0 (42)	26.5 (287)	27.2 (6794)
Cerebrovascular diseases	7.6 (25)	6.7 (15)	9.6 (151)	9.8 (3236)	14.9 (37)	11.7 (17)	15.2 (164)	14.9 (3737)
chronic obstructive	3.9 (13)	4.9 (11)	5.4 (85)	4.0 (1342)	6.0 (15)	3.4 (5)	6.1 (66)	5.4 (1340)

pulmonary disease								
Osteoarthritis	17.2 (57)	**9.3 (21)****	14.4 (226)	14.8 (4919)	11.2 (28)	**3.4 (5)****	9.2 (99)	12.4 (3110)

Comparisons made between participants and refusals, Gothenburg and Sweden.
Statistical analysis: to test differences between groups t test or χ2 test were used. *** p <0.001, ** p <0.01, * p <0.05; bolded numbers are significant at p <0.05; all other values are not significant.
[a] Information is missing on 3 individuals
Numbers in italics are not exact (true value within a 1-3 range) due to protection of the anonymity.

Table s7. Characteristics of 85 years old participants and refusals, and same aged individuals in Gothenburg and Sweden in 2015, stratified by sex

	Women				Men			
	**Participants**	**Refusals**	**Gothenburg**	**Sweden**	**Participants**	**Refusals**	**Gothenburg**	**Sweden**
N (%)	251[a] (60.3)	159[a] (62.1)	1 132 (62.6)	24 417 (60.6)	165 (39.7)	97[a] (37.9)	676 (37.4)	15 899 (39.4)
**Sociodemographic factors**								
Marital status, % (n)								
Married	22.4 (56)	20.9 (33)	23.1 (262)	23.3 (5700)	61.2 (101)	49.0 (47)	55.9 (378)	57.4 (9124)
Widowed	54.0 (135)	53.8 (85)	52.1 (590)	58.6 (14319)	24.8 (41)	27.1 (26)	25.7 (174)	24.2 (3853)
Divorced	18.8 (47)	22.2 (35)	19.8 (224)	**13.1 (3196)****	7.9 (13)	11.5 (11)	11.7 (79)	11.2 (1776)
Never married	4.8 (12)	3.2 (5)	4.9 (56)	4.9 (1202)	6.1 (10)	12.5 (12)	6.7 (45)	7.2 (1146)
Education, % (n)								
Elementary school	41.0 (*103*)	**58.0 (*92*)*****	46.8 (530)	**52.7 (12865)*****	33.0 (*54*)	**50.0 (*48*)****	38.2 (258)	**48.0 (7634)*****
Upper secondary school	39.6 (99)	**28.5 (45)***	**32.0 (362)***	**30.8 (7516)****	40.6 (67)	32.3 (31)	37.7 (255)	**32.5 (5171)***
Higher education	18.0 (45)	12.0 (19)	17.0 (193)	14.3 (3483)	24.8 (41)	**14.6 (14)***	21.0 (142)	**17.9 (2840)***
Born in Sweden, % (n)	84.0 (210)	82.3 (130)	80.4 (910)	86.8 (21194)	87.3 (144)	85.4 (82)	81.8 (553)	89.6 (14251)
Income, mean (SD)	14130 (10371)	14780 (21934)	14810 (25351)	13750 (20675)	18480 (8103)	15670 (17839)	17530 (15431)	17200 (23336)
Paid labour, % (n)	N/A	N/A	N/A	N/A	N/A	N/A	N/A	N/A
**Hospital discharge diagnoses,** % (n)								
Cancer	17.9 (45)	18.9 (30)	18.8 (213)	16.3 (3990)	23.6 (39)	16.5 (16)	22.3 (151)	20.4 (3246)
Neuropsychiatric diseases	23.1 (58)	23.3 (37)	24.5 (277)	25.3 (6175)	23.6 (39)	20.6 (20)	25.0 (169)	25.6 (4070)
Dementia	<4†	<4†	2.4 (27)	3.6 (877)	<4†	<4†	1.3 (9)	3.4 (536)
Cardiovascular diseases	58.6 (147)	60.4 (96)	60.5 (685)	63.2 (15428)	67.9 (112)	69.1 (67)	68.8 (465)	70.6 (11232)
Diabetes mellitus	6.8 (17)	10.1 (16)	9.0 (102)	**11.5 (2812)***	16.4 (27)	8.2 (8)	14.5 (98)	14.9 (2364)
Unipolar depression	3.6 (9)	<4†	5.8 (66)	4.5 (1109)	6.1 (10)	<4†	4.3 (29)	**2.8 (446)***
Alcohol-related disorders	<4†	<4†	0.5 (6)	0.5 (125)	<4†	<4†	2.2 (15)	1.7 (272)
Ischemic heart diseases	12.0 (30)	11.9 (19)	16.4 (186)	**18.7 (4561)****	27.9 (46)	33.0 (32)	30.9 (209)	32.3 (5137)
Cerebrovascular diseases	10.4 (26)	16.4 (26)	13.3 (150)	14.1 (3440)	16.4 (27)	18.6 (18)	18.0 (122)	19.3 (3063)
chronic obstructive	4.8 (12)	5.0 (8)	4.8 (54)	5.2 (1272)	4.8 (8)	4.1 (4)	7.0 (47)	7.1 (1133)

pulmonary disease								
Osteoarthritis	23.9 (60)	17.0 (27)	21.9 (248)	21.2 (5188)	13.9 (23)	7.2 (7)	12.9 (87)	16.8 (2666)

Comparisons made between participants and refusals, Gothenburg and Sweden.
Statistical analysis: to test differences between groups t test or χ2 test were used. *** p <0.001, ** p <0.01, * p <0.05; bolded numbers are significant at p <0.05; all other values are not significant.
[a] One individual is missing in the sociodemographic variables due personal identity number not found in the register.
[b] Information is missing on 2 individuals
Numbers in italics are not exact (true value within a 1-3 range) due to protection of the anonymity.
N/A Not applicable as the cut of age for paid labour in Statistics Sweden registers is 84.
†Less than four individuals. exact number not presented to protect anonymity. Statistical comparison not performed.

Table s8. Characteristics of 88 years old participants and refusals, and same aged individuals in Gothenburg and Sweden in 2018

	Women				Men			
	**Participants**	**Refusals**	**Gothenburg**	**Sweden**	**Participants**	**Refusals**	**Gothenburg**	**Sweden**
N (%)	162 (62.8)	151 (61.1)	850 (64.3)	18589 (63.1)	96 (37.2)	96[a] (38.9)	471 (35.7)	10883 (36.9)
**Sociodemographic factors**								
Marital status, % (n)								
Married	10.5 (17)	15.9 (24)	**17.2 (146)****	**16.6 (3078)****	54.2 (52)	51.6 (49)	53.1 (250)	51.9 (5650)
Widowed	69.1 (112)	61.6 (93)	**59.8 (508)****	66.3 (12325)	34.4 (33)	27.4 (26)	30.4 (143)	31.0 (3375)
Divorced	16.0 (26)	19.9 (30)	18.2 (155)	12.4 (2309)	7.3 (7)	10.5 (10)	10.8 (51)	10.6 (1154)
Never married	4.3 (7)	2.6 (4)	4.8 (41)	4.7 (877)	4.2 (4)	10.5 (10)	5.7 (27)	6.5 (704)
Education, % (n)								
Elementary school	37.7 (61)	**54.3 (82)****	45.6 (388)	**51.8 (9625)*****	37.5 (36)	38.9 (37)	36.1 (170)	46.1 (5017)
Upper secondary school	36.0 (*60*)	29.1 (44)	32.1 (273)	30.9 (5741)	33.0 (*34*)	39.0 (*38*)	38.9 (183)	33.1 (3606)
Higher education	23.5 (38)	**13.2 (20)***	17.9 (152)	**15.0 (2792)****	24.0 (23)	16.8 (16)	21.2 (100)	19.2 (2087)
Born in Sweden, % (n)	82.7 (134)	82.8 (125)	80.0 (680)	87.0 (16165)	84.4 (81)	82.1 (78)	81.5 (384)	89.7 (9765)
Income, mean (SD)	19590 (30923)	16200 (19687)	16200 (22412)	15600 (23116)	18850 (13561)	19800 (20415)	19320 (16731)	18960 (29208)
Paid labour, % (n)	N/A	N/A	N/A	N/A	N/A	N/A	N/A	N/A
**Hospital discharge diagnoses,** % (n)								
Cancer	19.1 (31)	20.5 (31)	19.8 (168)	17.4 (3240)	20.8 (20)	19.8 (19)	22.9 (108)	22.0 (2394)
Neuropsychiatric diseases	24.7 (40)	**40.4 (61)****	28.1 (239)	29.0 (5384)	25.0 (24)	26.0 (25)	27.0 (127)	29.0 (3159)
Dementia	<4†	3.3 (5)	2.1 (18)	4.5 (831)	<4†	5.2 (5)	1.9 (9)	4.2 (458)
Cardiovascular diseases	66.0 (107)	67.5 (102)	66.9 (569)	70.3 (13063)	63.5 (61)	75.0 (72)	72.2 (340)	**75.5 (8214)****
Diabetes mellitus	11.7 (19)	9.3 (14)	9.5 (81)	12.5 (2318)	12.5 (12)	16.7 (16)	14.4 (68)	15.6 (1701)
Unipolar depression	5.6 (9)	6.0 (9)	6.4 (54)	4.7 (870)	6.3 (6)	4.2 (4)	4.0 (19)	2.9 (321)
Alcohol-related disorders	0 (0)	<4†	0.5 (4)	0.4 (74)	0 (0)	<4†	2.1 (10)	1.5 (168)
Ischemic heart diseases	13.6 (22)	13.2 (20)	16.6 (141)	**20.0 (3726)***	25.0 (24)	32.3 (31)	29.9 (141)	33.8 (3679)
Cerebrovascular diseases	14.2 (23)	17.2 (26)	14.8 (126)	15.3 (2850)	15.6 (15)	14.6 (14)	17.6 (83)	20.2 (2194)
chronic obstructive	5.6 (9)	<4†	4.4 (37)	5.5 (1020)	5.2 (5)	<4†	5.9 (28)	7.1 (773)

pulmonary disease								
Osteoarthritis	29.0 (47)	**18.5 (28)***	24.5 (208)	23.1 (4292)	15.6 (15)	10.4 (10)	14.4 (68)	18.2 (1976)

Comparisons made between participants and refusals, Gothenburg and Sweden.
Statistical analysis: to test differences between groups t test or χ2 test were used. *** p <0.001, ** p <0.01, * p <0.05; bolded numbers are significant at p <0.05; all other values are not significant.
Numbers in italics are not exact (true value within a 1-3 range) due to protection of the anonymity.
N/A Not applicable as the cut of age for paid labour in Statistics Sweden registers is 84.
†Less than four individuals. exact number not presented to protect anonymity. Statistical comparison not performed.
