# Supplementary material for: Representativeness in population-based studies of older adults: five waves of cross-sectional examinations in the Gothenburg H70 Birth Cohort Study
Source: BMJ Open. 2022 Dec 16;12(12):e068165. doi: 10.1136/bmjopen-2022-068165 (PMC9764666; doi:10.1136/bmjopen-2022-068165)
Supplement: Supplementary data [file bmjopen-2022-068165supp002.pdf]

Fig s1. Response rates in different subgroups at all examination years

	2000			2005			2009			2015			2018		
	**Total**	**Women**	**Men**	**Total**	**Women**	**Men**	**Total**	**Women**	**Men**	**Total**	**Women**	**Men**	**Total**	**Women**	**Men**
**Response rate**	69.5% (523/752)	72.1% (281/390)	66.9% (242/362)	64.1% (765/1194)	64.0% (438/684)	64.1% (327/510)	61.1% (580/950)	59.5% (331/556)	63.2% (249/394)	62.0% (415/669)	61.3% (250/408)	63.2% (165/261)	51.2% (258/504)	51.8% (162/313)	50.3% (96/191)
**Marital status,** *% (n/N)*															
Married, *ref*	71.8% (311/433)	70.7% (130/184)	72.7% (181/249)	67.1% (418/623)	64.0% (183/286)	69.7% (235/337)	63.6% (287/451)	57.1% (113/198)	68.8% (174/253)	66.2% (157/237)	62.9% (56/89)	68.2% (101/148)	48.6% (69/142)	41.5% (17/41)	51.5% (52/101)
Widowed	69.4% (77/111)	74.5% (70/94)	**41.2%** (7/17)**	64.8% (171/264)	68.7% (145/211)	**49.1%** (26/53)**	61.7% (166/269)	63.4% (135/213)	55.4% (31/56)	61.3% (176/287)	61.4% (135/220)	61.2% (41/67)	54.9% (145/264)	54.6% (112/205)	55.9% (33/59)
Divorced	72.1% (101/140)	75.6% (65/86)	66.7% (36/54)	**58.5%* (131/224)**	58.3% (88/151)	58.9% (43/73)	**54.3%* (89/164)**	55.6% (65/117)	**51.1%* (24/47)**	56.6% (60/106)	57.3% (47/82)	54.2% (13/24)	45.2% (33/73)	46.4% (26/56)	41.2% (7/17)
Unmarried	**50.0%*** (34/68)**	61.5% (16/26)	**42.9%*** (18/42)**	**54.2%* (45/83)**	61.1% (22/36)	**48.9%** (23/47)**	57.6% (38/66)	64.3% (18/28)	**52.6%* (20/38)**	56.4% (22/39)	70.6% (12/17)	**45.5%* (10/22)**	44.0% (11/25)	63.6% (7/11)	28.6% (4/14)
**Education,** *% (n/N)*															
Elementary school ≤9y, *ref*	65.5% (253/386)	70.5% (153/217)	59.2% (100/169)	57.4% (340/592)	59.3% (213/359)	54.5% (127/233)	51.9% (234/451)	50.3% (144/286)	54.5% (90/165)	52.9% (157/297)	52.8% (103/195)	52.9% (54/102)	44.9% (97/216)	42.7% (61/143)	49.3% (36/73)
Upper secondary school	**73.8%* (177/240)**	78.8% (89/113)	69.3% (88/127)	**72.4%* (278/384)**	**73.0%*** (157/215)**	**71.6%*** (121/169)**	**69.4%*** (225/324)**	**70.4%*** (126/179)**	**68.3%* (99/145)**	**68.6%*** (166/242)**	**68.8%** (99/144)**	**68.4%* (67/98)**	53.4% (94/176)	**57.7%* (60/104)**	47.2% (34/72)
Higher education	**76.8%* (86/112)**	66.7% (34/51)	**85.2%*** (52/61)**	**71.9%* (141/196)**	67.3% (66/98)	**76.5%*** (75/98)**	**70.8%*** (119/168)**	**67.8%** (59/87)**	**74.1%** (60/81)**	**72.3%*** (86/119)**	**70.3%* (45/64)**	**74.5%** (41/55)**	**62.9%** (61/97)**	**65.5%** (38/58)**	59.0% (23/39)
**Ethnicity**, *% (n/N)*															
Born in Sweden, *ref*	71.5% (438/613)	74.1% (235/317)	68.6% (203/296)	64.8% (633/977)	65.0% (366/563)	64.5% (267/414)	60.5% (489/808)	58.8% (277/471)	62.9% (212/337)	62.5% (354/566)	61.8% (210/340)	63.7% (144/226)	51.4% (215/418)	51.7% (134/259)	50.9% (81/159)
Not born in Sweden	**61.2%* (85/139)**	63.0% (46/73)	59.1% (39/66)	60.8% (132/217)	59.5% (72/121)	62.5% (60/96)	64.1% (91/142)	63.5% (54/85)	64.9% (37/57)	59.2% (61/103)	58.8% (40/68)	60.0% (21/35)	50.0% (43/86)	51.9% (28/54)	46.9% (15/32)

The colour shading indicate response rate. The more dark colour, the higher response rate in comparison to the other subgroups the same examination year.

Comparisons made between the different subgroups at each examination year in the total groups, among women, and among men.

Statistical analysis: to test differences between groups χ^2 test were used. *** p <0.001, ** p <0.01, * p <0.05; bolded numbers are significant at p <0.05; all other values are not significant.
