# Supplementary material for: Representativeness in population-based studies of older adults: five waves of cross-sectional examinations in the Gothenburg H70 Birth Cohort Study
Source: BMJ Open. 2022 Dec 16;12(12):e068165. doi: 10.1136/bmjopen-2022-068165 (PMC9764666; doi:10.1136/bmjopen-2022-068165)
Supplement: Author's manuscript [file bmjopen-2022-068165.draft_revisions.pdf]

BMJ Open

BMJ Open is committed to open peer review. As part of this commitment we make the peer review history of every article we publish publicly available.

When an article is published we post the peer reviewers' comments and the authors' responses online. We also post the versions of the paper that were used during peer review. These are the versions that the peer review comments apply to.

The versions of the paper that follow are the versions that were submitted during the peer review process. They are not the versions of record or the final published versions. They should not be cited or distributed as the published version of this manuscript.

BMJ Open is an open access journal and the full, final, typeset and author-corrected version of record of the manuscript is available on our site with no access controls, subscription charges or pay-per-view fees (http://bmjopen.bmj.com).

If you have any questions on BMJ Open's open peer review process please email info.bmjopen@bmj.com

BMJ Open

Representativeness in population-based studies of older adults – Five waves of cross-sectional examinations in the Gothenburg H70 Birth Cohort Study

Journal:	*BMJ Open*
Manuscript ID	bmjopen-2022-068165
Article Type:	Original research
Date Submitted by the Author:	09-Sep-2022
Complete List of Authors:	Wetterberg, Hanna; University of Gothenburg Institute of Neuroscience and Physiology, Department of Psychiatry and Neurochemistry; University of Gothenburg, Centre for Ageing and Health (AGECAP)<br>Rydén, Lina; University of Gothenburg Institute of Neuroscience and Physiology, Department of Psychiatry and Neurochemistry,; University of Gothenburg, Centre for Ageing and Health (AGECAP)<br>Ahlner, Felicia; University of Gothenburg Institute of Neuroscience and Physiology, Department of Psychiatry and Neurochemistry; University of Gothenburg, Centre for Ageing and Health (AGECAP)<br>Falk, Hanna; Goteborgs universitet Sahlgrenska Akademin, Institute of Health and Care Sciences; Institute of Neuroscience and Physiology<br>Gudmundsson, Pia; University of Gothenburg Institute of Neuroscience and Physiology, Department of Psychiatry and Neurochemistry; University of Gothenburg, Centre for Ageing and Health (AGECAP)<br>Guo, Xinxin; University of Gothenburg Institute of Neuroscience and Physiology, Department of Psychiatry and Neurochemistry; University of Gothenburg, Centre for Ageing and Health (AGECAP)<br>Joas, Erik; University of Gothenburg Institute of Neuroscience and Physiology, Department of Psychiatry and Neurochemistry; University of Gothenburg, Centre for Ageing and Health (AGECAP)<br>Johansson, Lena; University of Gothenburg Sahlgrenska Academy, Institute of Health and Care Sciences; University of Gothenburg Sahlgrenska Academy, Department of Psychiatry and Neurochemistry<br>Kern, Silke; University of Gothenburg Institute of Neuroscience and Physiology, Department of Psychiatry and Neurochemistry; University of Gothenburg, Centre for Ageing and Health (AGECAP)<br>Fässberg, Madeleine; University of Gothenburg Institute of Neuroscience and Physiology, Psychiatry and Neurochemistry; University of Gothenburg, Department of Psychiatry and Neurochemistry<br>Najar, Jenna; University of Gothenburg Institute of Neuroscience and Physiology, Department of Psychiatry and Neurochemistry; University of Gothenburg, Centre for Ageing and Health (AGECAP)<br>Ribbe, Mats; University of Gothenburg Institute of Neuroscience and Physiology, Department of Psychiatry and Neurochemistry; University of London, Centre for Ageing and Health (AGECAP)<br>Sterner, Therese Rydberg; University of Gothenburg Institute of Neuroscience and Physiology, Department of Psychiatry and Neurochemistry; University of Gothenburg, Centre for Ageing and Health (AGECAP)

Samuelsson, Jessica; University of Gothenburg Institute of Neuroscience and Physiology, Department of Psychiatry and Neurochemistry; University of Gothenburg, Centre for Ageing and Health (AGECAP)
Sacuiu, S; University of Gothenburg Institute of Neuroscience and Physiology, Department of Psychiatry and Neurochemistry; University of Gothenburg, Centre for Ageing and Health (AGECAP)
Sigström, Robert; University of Gothenburg Institute of Neuroscience and Physiology, Department of Psychiatry and Neurochemistry; University of Gothenburg, Centre for Ageing and Health (AGECAP)
Skoog, Johan; University of Gothenburg Institute of Neuroscience and Physiology, Department of Psychiatry and Neurochemistry; University of Gothenburg, Centre for Ageing and Health (AGECAP)
Waern, M; University of Gothenburg Institute of Neuroscience and Physiology, Department of Psychiatry and Neurochemistry; University of Gothenburg, Centre for Ageing and Health (AGECAP)
Zettergren, Anna; University of Gothenburg Institute of Neuroscience and Physiology, Department of Psychiatry and Neurochemistry; University of Gothenburg, Centre for Ageing and Health (AGECAP)
Skoog, Ingmar; University of Gothenburg Institute of Neuroscience and Physiology, Department of Psychiatry and Neurochemistry; University of Gothenburg, Centre for Ageing and Health (AGECAP)

Keywords: EPIDEMIOLOGY, STATISTICS & RESEARCH METHODS, Health informatics < BIOTECHNOLOGY & BIOINFORMATICS

SCHOLARONE™ Manuscripts

I, the Submitting Author has the right to grant and does grant on behalf of all authors of the Work (as defined in the below author licence), an exclusive licence and/or a non-exclusive licence for contributions from authors who are: i) UK Crown employees; ii) where BMJ has agreed a CC-BY licence shall apply, and/or iii) in accordance with the terms applicable for US Federal Government officers or employees acting as part of their official duties; on a worldwide, perpetual, irrevocable, royalty-free basis to BMJ Publishing Group Ltd ("BMJ") its licensees and where the relevant Journal is co-owned by BMJ to the co-owners of the Journal, to publish the Work in this journal and any other BMJ products and to exploit all rights, as set out in our licence.

The Submitting Author accepts and understands that any supply made under these terms is made by BMJ to the Submitting Author unless you are acting as an employee on behalf of your employer or a postgraduate student of an affiliated institution which is paying any applicable article publishing charge ("APC") for Open Access articles. Where the Submitting Author wishes to make the Work available on an Open Access basis (and intends to pay the relevant APC), the terms of reuse of such Open Access shall be governed by a Creative Commons licence – details of these licences and which Creative Commons licence will apply to this Work are set out in our licence referred to above.

Other than as permitted in any relevant BMJ Author's Self Archiving Policies, I confirm this Work has not been accepted for publication elsewhere, is not being considered for publication elsewhere and does not duplicate material already published. I confirm all authors consent to publication of this Work and authorise the granting of this licence.

For peer review only

Representativeness in population-based studies of older adults – Five waves of cross-sectional examinations in the Gothenburg H70 Birth Cohort Study

Hanna Wetterberg, MSc[ab]*, Lina Rydén, MD[abc]*, Felicia Ahlner, MSc[ab], Hanna Falk Erhag, RN, PhD[ab], Pia Gudmundsson, PhD[ab], Xinxin Guo, MD, PhD[ab],[d], Erik Joas, PhD[ab], Lena Johansson, RN, PhD[ab], Silke Kern, MD, PhD[abc], Madeleine Mellqvist Fässberg, PhD[ab], Jenna Najar, MD, PhD[abc], Mats Ribbe, MD, PhD[ab], Therese Rydberg Sterner, PhD[abe], Jessica Samuelsson, MSc[ab], Simona Sacuiu MD, PhD[abc], Robert Sigström, MD, PhD[abc], Johan Skoog, PhD[ab], Margda Waern, MD, PhD[abf], Anna Zettergren, PhD[ab], Ingmar Skoog, MD, PhD[abc]

* HW and LR are joint first authors

[a]*Neuropsychiatric Epidemiology Unit, Department of Psychiatry and Neurochemistry, Institute of Neuroscience and Physiology, Sahlgrenska Academy, at the University of Gothenburg, Sweden;* [b]*Centre for Ageing and Health (AGECAP) at the University of Gothenburg, Sweden;* [c]*Region Västra Götaland, Sahlgrenska University Hospital, Psychiatry, Cognition and Old Age Psychiatry Clinic, Gothenburg, Sweden;* [d]*Region Västra Götaland, Sahlgrenska University Hospital, Department of Mood disorders, Gothenburg, Sweden;* [e]*Aging Research Center, Department of Neurobiology, Care Sciences and Society, Karolinska Institutet and Stockholm University, Stockholm, Sweden;* [f]*Region Västra Götaland, Department of Psychiatry, Psychotic Disorders, Sahlgrenska University Hospital, Mölndal, Sweden*

E-mail addresses and ORCID for authors

Hanna Wetterberg hanna.wetterberg@gu.se (0000-0003-3836-825X)
Lina Rydén <lina.ryden@gu.se> (0000-0002-3817-2620)
Felicia Ahlner felicia.ahlner@gu.se (0000-0003-3116-7332)
Hanna Falk Erhag hanna.falk@gu.se (0000-0002-6494-2895)
Pia Gudmundsson pia.gudmundsson@gu.se (0000-0001-7173-4341)
Xinxin Guo xinxin.guo@gu.se (0000-0002-1353-287X)
Erik Joas erik.joas@gu.se (0000-0001-9546-2192)
Lena Johansson lena.maria.johansson@gu.se (0000-0002-9928-4909)
Silke Kern silke.kern@gu.se (0000-0001-7617-7214)
Madeleine Mellqvist Fässberg madeleine.mellqvist@gu.se (0000-0002-7366-4444)
Jenna Najar jenna.najar@gu.se (0000-0002-1762-4413)
Mats Ribbe mats.ribbe@gu.se (0000-0002-5998-656X)
Therese Rydberg Sterner therese.rydberg.sterner@gu.se (0000-0001-8202-8522),

For peer review only - http://bmjopen.bmj.com/site/about/guidelines.xhtml

Jessica Samuelsson jessica.samuelsson@gu.se (0000-0002-8891-6720)
Simona Sacuiu simona.sacuiu@neuro.gu.se (0000-0003-0472-7699)
Robert Sigström robert.sigstrom@gu.se (0000-0003-1505-1532)
Johan Skoog johan.skoog@psy.gu.se (0000-0001-9549-5875)
Margda Waern margda.waern@gu.se (0000-0002-8330-6915)
Anna Zettergren anna.zettergren@gu.se (0000-0002-7182-8417)
Ingmar Skoog ingmar.skoog@neuro.gu.se (0000-0002-9803-3438)

Corresponding author:

Hanna Wetterberg, Neuropsychiatric Epidemiology Unit, Wallinsgatan 6, SE 431 41 Mölndal

+46733424288, hanna.wetterberg@gu.se

Number of words: 3276

For peer review only

Abstract

Objectives: To describe representativeness in the Gothenburg H70 1930 birth cohort study.

Design: Repeated cross-sectional examinations of a population-based study.

Setting: Gothenburg, Sweden.

Participants: All residents in Gothenburg, Sweden, born on specific birth dates during 1930 were invited to a comprehensive health examination at ages 70, 75, 79, 85, and 88. The numbers of participants at each examination were: 524 at age 70, 767 at age 75, 580 at age 79, 416 at age 85, and 258 at age 88.

Primary outcome measures: We compared register data on sociodemographic characteristics and hospital discharge diagnoses between participants and a) refusals, b) all same-aged individuals in Gothenburg, and c) all same-aged individuals in Sweden. We also compared mortality rate between participants and refusals.

Results: Refusal rate increased with age. At two or more examination waves, participants compared to refusals, had higher educational level, more often osteoarthtitis, lower mortality rates, lower prevalence of neuropsychiatric, alcohol-related and cardiovascular disorders, and were more often married. At two examination waves, paricipants compared to same-aged individuals in Gothenburg, had higher education and were more often born in Sweden. At two examination waves or more, participants compared to same-aged individuals in Sweden, had higher education, higher average income, less often ischemic heart disease, were less often born in Sweden, and more often divorced.

Conclusion: Participants were more similar to the target population in Gothenburg than to refusals and same-aged individuals in Sweden. Our study shows the importance to have different comparison groups when assessing representativeness of population studies, which is important in evaluating generalizibility of results. The study also contribute with uniqe and up-to-date knowledge on participation bias in these high age groups.

Keywords: Epidemiologic studies, cohort studies, aged, selection bias

For peer review only

Article summary

Strengths and limitations of this study

- The use of multiple comparison groups provide useful information on how to quantify the representativess of population-based studies on older adults
- Included in the comparisons are a broad range of register-based variables providing information on the differences and similarities between the groups
- The data was received on an aggregated level due to ethical regulations, hence, only univariate analyses were possible to perform

For peer review only

Introduction

Cross-sectional population-based studies are important to measure the societal burden of physical and mental disorders and associated factors, as well as providing baseline data for incidence studies (1, 2). Biased participation decreases representativeness and may affect results of the studies and lead to spurious findings. This occurs when those more prone to accept have specific characteristics (2). One well-known potential bias is the "healthy volunteer effect", where participants have a lower disease burden than refusals. Other factors known to decrease representativeness are that women and individuals with higher socioeconomic status and higher education tend to participate more often (1, 3). Studies with low response rates are more vulnerable to self-selection bias than studies with high response rates.

Representativeness could be examined in different ways. One is to compare characteristics between participants and refusals. Another is to compare participants to the target population from which the sample was drawn. A third is to compare participants to the population of a larger geographical area, such as the country where the study was performed.

The Gothenburg H70 Birth Cohort Studies (the H70 studies) are multidisciplinary population studies examining birth cohorts of older populations in Gothenburg, Sweden. The aim is to study ageing and the prevalence of mental and physical disorders and their risk factors, considering the complex interactions with age, sex, gender, socio-economic gradients, secular changes, as well as psychosocial, neurobiological, and genetic factors occurring across the life course (4). The studies started in 1971 (5) and since then, five birth cohorts of 70–year olds (4), and three birth cohorts of 85-year olds (6) have been examined and followed longitudinally. The present study is concerned with the 1930 cohort, where we previously have published more than 30 papers based on cross-sectional examinations. However, no study has examined the representativeness of this cohort in detail, or made comparisons with the whole populations of Gothenburg and Sweden, or given a detailed overview of sampling methods for all examinations performed at ages 70, 75, 79, 85,

For peer review only - http://bmjopen.bmj.com/site/about/guidelines.xhtml

and 88. This type of information is important to evaluate the external validity of a study, and is essential to assess if the representativeness of studies changes over time (7). This setting gives a unique opportunity to evaluate in detail participation bias in an older population.

This paper aimed to describe the representativeness of participants of the examinations of the birth cohort born in 1930, at ages 70, 75, 79, 85, and 88 years. We studied three levels of representativeness; in relation to refusals, same-aged individuals in Gothenburg, and same-aged individuals in Sweden.

Methods

The H70 birth cohort 1930 was examined at five occasions. All samples included residents in Gothenburg born in 1930, who were systematically selected based on birth dates. An exception was in 2009-2011 when only those invited previously were included. Individuals who were not traceable or did not speak Swedish were excluded from the eligible samples. The invitation procedure has been nearly identical at all examination years. An invitation letter was sent to all sampled individuals, including information about the study and a consent form. After 1-2 weeks, the research staff contacted invited individuals by telephone. In cases of no contact or when the telephone number could not be found, reminders were posted. Individuals who were not able to visit the outpatient clinic were offered a home visit.

The H70 birth cohort 1930 includes participants born 1930 from the Prospective Population Study of Women (PPSW), a longitudinal study with baseline examination in 1968-1969 (8). The number of participants examined before 2000 within the PPSW study was 173 (33%) in 2000, 155 (20%) in 2005, 116 (20%) in 2009, 81 (20%) in 2015, and 53 (21%) in 2018.

Data collection procedures

For peer review only - http://bmjopen.bmj.com/site/about/guidelines.xhtml

Examinations

The examination procedures have been similar at all examinations (4), and include a six to eight hour general examination. In addition, participants were invited to a range of additional examinations and a close informant interview. See Additional file, Table s1 for details regarding the specific examinations conducted at the different waves.

Comparison groups

We investigated the representativeness of the participants in the study in comparison to the refusals, same-aged indivuals in Gothenburg and same-aged indivuals in Sweden. Included in the comparison groups of Gothenburg and Sweden are all indivudals born in 1930 who are registered residents in the municipality of Gothenburg and in Sweden, respectively. Hence, in the Gothenburg group indivudals that participated in the study is included as well. This data was received from Statistics Sweden on an aggregated level.

Outcome variables

Information on sociodemographic variables for participants, refusals, and same-aged individuals from the municipality of Gothenburg as well as Sweden were received on an aggregated level from Statistics Sweden, and included marital status (married/widowed/divorced/never married), highest attained educational level (elementary/upper secondary/higher), country of birth (Sweden, yes/no), paid labour (occupational status, yes/no), and average individual income per month (means and standard deviations (SD)).

Hospital discharge diagnoses were obtained from the inpatient part of the National Patient Register (NPR), using codes from the Swedish version of the International Classification of Diseases (ICD) 9 and ICD 10. Diagnoses from 1987 up to each examination year were used in the analyses. Data was available at an individual level for participants and refusals in 2000, 2005, and 2009. Data on same-aged individuals living in Gothenburg and Sweden in 2000, 2005, 2009, 2015

For peer review only - http://bmjopen.bmj.com/site/about/guidelines.xhtml

and 2018, and data on participants and non-participants at the examinations in 2015 and 2018 were received on aggregated level from the National Board of Health and Welfare. The NPR gained full national coverage in 1987 and all citizens in Sweden have access to medical care and therefore equal chance to be included in the NPR (9). The hospital discharge diagnoses included cancer, neuropsychiatric disorders (including dementia disorders), cardiovascular diseases, diabetes mellitus, unipolar depression, alcohol-related disorders, ischemic heart disease, cerebrovascular diseases, chronic obstructive pulmonary disease, and osteoarthritis (see Additional file, Table s2, for ICD codes).

Dates of death were available from the Swedish Tax Agency.

Statistical analyses

Differences in sociodemographic factors and hospital discharge diagnoses between participants and refusals, same-aged individuals in Gothenburg and same-aged individuals in Sweden were compared using Pearson's chi-squared test or Fisher's exact test for categorical variables and independent samples t-test for continuous variables. As the data was revieced on an aggregated level, only univariate analyses were performed.

Cox proportional hazards models adjusted for sex were used to compare mortality between participants and refusals, presented as hazard ratios (HR) and 95% confidence intervals (CI). The risk time was calculated as the surviving time up to five years from age 70, 75, 79, 85, and two years from age 88. Individuals who emigrated during follow-up were excluded from mortality analyses, as their status is not known (four from examination in 2000, two from 2005, none from 2009, and one each from 2015 and 2018). The proportional hazard assumption was verified using Schoenfeld residuals. Mortality analyses were performed in R (version 3.5.3) using survminer and survival packages. P<0.05 (two-tailed) was considered statistically significant in all analyses.

Figure 1 to be placed here

For peer review only - http://bmjopen.bmj.com/site/about/guidelines.xhtml

Participant and public involvement statement

It was not possible to involve participants or the public in our research's design, conduction, reporting or dissemination plans. This report complied with the Strengthening the Reporting of Observational Studies in Epidemiology guidelines for observational studies (including cross-sectional studies), as stated in the Research Checklist (10).

Results

Samples (Figure 1)

The sample flow charts are displayed in Figure 1.

In 2000, 775 70-year-old residents in Gothenburg were sampled. Among these, 753 were eligble (see Figure 1), and 524 accepted to participate (response rate: 69.6%). Of these, 14 (2.7%) were examined in their home. The study was conducted September 4, 2000 - March 28, 2002.

In 2005, 1250 75-year-old residents in Gothenburg were sampled. Among these, 1196 were eligble (see Figure 1), and 767 accepted to participate (response rate: 64.1%). Of these, 117 (15.3%) were examined in their home. The study was conducted September 6, 2005 - March 20, 2007.

In 2009, 1056 79-year-old residents in Gothenburg, who had been invited at the 2000 or 2005 examinations were invited, and 950 were eligble (see Figure 1), and 580 accepted to participate (response rate: 61.1%). Of these, 144 (24.8%) were examined in their home, and 6 (1.0%) had a combination of clinic and at home examinations. The study was conducted November 6, 2009 - September 19, 2011.

In 2015, 764 85-year-old residents in Gothenburg were sampled. Among these, 672 were eligble (see Figure 1), and 416 accepted to participate (eligible response rate: 61.9%). Of these,

For peer review only - http://bmjopen.bmj.com/site/about/guidelines.xhtml

174 (41.8%) were examined in their home, and 18 (4.3%) had a combination of clinic and home examinations. The study was conducted October 7, 2015 - May 29, 2017.

In 2018, 555 88-year-old residents in Gothenburg were sampled. Among these, 505 were eligble (see Figure 1), and 258 accepted to participate (eligible response rate: 51.1%). Of these, 131 (50.8%) were examined in their home, and 11 (4.3%) had a combination of clinic and home examinations. The study was conducted September 24, 2018 - December 20, 2019.

Results

The mean age of those examined was 70.6 years (SD 0.3) in 2000, 75.7 years (SD 0.4) in 2005, 80.1 years (SD 0.2) in 2009, 86.0 years (SD 0.2) in 2015, and 88.6 years (SD 0.2) in 2018. Among those who participated, 74.1% (n=811) participated more than once.

The response rate was higher in 2000 and lower in 2018 compared to all other examinations (Table 1). There were no differences in response rates between women and men (see Additional file 2, Figure s1). For response rates in various subgroups, see Additional file 2, Figure s1. The proportion excluded from the sample because they did not speak Swedish, died after sampling, or were non-traceable was higher in 2009, 2015, and 2018 compared to 2000 and 2005 (Additional file 1, Table s3).

Table 1. Comparison of response rates between examinations

Exam. Year<br>Response rate	2000<br>69.5%	2005<br>64.1%	2009<br>61.1%	2015<br>62.0%	2018<br>51.2%
2000<br>69.5%	-	$p = 0.013$	$p < 0.000$	$p = 0.003$	$p < 0.000$
2005<br>64.1%		-	$p = 0.151$	$p = 0.381$	$p < 0.000$
2009<br>61.1%			-	$p = 0.690$	$p < 0.000$
2015<br>62.0%				-	$p < 0.000$
2018<br>51.2%					-
Statistical analysis: to test differences between groups χ2 test were used.					

For peer review only - http://bmjopen.bmj.com/site/about/guidelines.xhtml

For peer review only

Mortality

Mortality was higher in refusals compared to participants at all examination years (Table 2). The test of Schoenfeld's residuals indicated deviation from the proportional hazards assumption in the survival analysis in 2005, showing that the higher mortality among refusals was limited to the first two years (Table 2).

Table 2. Mortality rates in participants and refusals

	HR	95% CI	Number of deaths (%)
70-year olds invited in 2000			
Participants (n=522)	Ref		21 (4.0)
Refusals (n=227)	3.7	2.1-6.3	33 (14.5)
75-year olds invited in 2005			
Year 0-2			
Participants (n=690)	Ref		16 (2.3)
Refusals (n=377)	4.8	2.7-8.6	41 (10.9)
Year 3-5			
Participants (n=751)	Ref		77 (10.3)
Participants (n=386)	1.3	0.9-1.9	50 (13.0)
79-year olds invited in 2009			
Participants (n=580)	Ref		107 (18.4)
Refusals (n=370)	1.6	1.2-2.1	97 (26.2)
85-year olds invited in 2015			
Participants (n=415)	Ref		128 (30.8)
Refusals (n=256)	1.6	1.2-2.1	114 (44.5)
88-year olds invited in 2018			
Participants (n=258)	Ref		23 (8.9)
Refusals (n=246)	2.8	1.7-4.5	56 (22.8)

Mortality rates derived from Cox regression analysis, adjusted by sex. HR: Hazard ratio, CI: confidence interval.
2000: 5-year mortality from age 70
2005: 0-2 year and 3-5 year mortality (the 5 year survival analysis deviated from the proportional hazards assumption, showing that the higher mortality among refusals was limited to the first 2 years) from age 75
2009: 5-year mortality from age 79
2015: 5-year mortality from age 85
2018: 2-year mortality from age 88.

For peer review only

Sociodemographic factors and hospital discharge diagnoses from registers

Table 3 shows sociodemographic factors and hospital discharge diagnoses for participants, refusals, and all same-aged individuals living in Gothenburg and in Sweden during the corresponding year. Analyses stratified by sex are shown in Additional file 1, Table s4-s8.

For peer review only

For peer review only - http://bmjopen.bmj.com/site/about/guidelines.xhtml

Table 3. Characteristics of 70-, 75- and 79-year-old participants and refusals, and same aged individuals in Gothenburg and Sweden in 2000, 2005 and 2009

	70-year olds in 2000				75-year olds in 2005				79-year olds in 2009			
	Participants	Refusals	Gothenburg	Sweden	Participants	Refusals	Gothenburg	Sweden	Participants	Refusals	Gothenburg	Sweden
N	524[a]	229	3508	75 468	767[a]	429	3075	66 855	580	370	2647	58184
Sociodemographic factors												
Marital status, % (n)												
Married	59.5 (311)	53.3 (122)	56.1 (1968)	61.9 (46 697)	54.6 (418)	**47.8 (205)***	52.4 (1 611)	55.7 (37 238)	49.5 (287)	44.3 (164)	46.8 (1 238)	49.4 (28 736)
Widowed	14.7 (77)	14.8 (34)	15.8 (553)	16.3 (12 291)	22.4 (171)	21.7 (93)	21.5 (661)	23.5 (15 715)	28.6 (166)	27.8 (103)	28.3 (748)	30.9 (17 973)
Divorced	19.3 (101)	17.0 (39)	19.4 (679)	**14.0 (10 576)*****	17.1 (131)	21.7 (93)	18.2 (560)	**13.5 (9 051)****	15.3 (89)	20.3 (75)	17.9 (473)	13.0 (7 550)
Never married	6.5 (34)	**14.8 (34)*****	8.8 (308)	7.8 (5 904)	5.9 (45)	8.9 (38)	7.9 (243)	7.3 (4 851)	6.6 (38)	7.6 (28)	7.1 (188)	6.7 (3 925)
Education, % (n)												
Elementary school	48.3 (*253*)	**58.1 (*133*)***	47.8 (1678)	**53.9 (40 694)***	44.4 (*340*)	**58.7 (252)*****	47.7 (1 467)	**53.2 (35 584)*****	40.3 (*234*)	**58.6 (217)*****	**45.6 (1 206)***	**52.5 (30 556)*****
Upper secondary school	33.8 (177)	27.5 (63)	32.8 (1149)	30.5 (23 027)	36.3 (278)	**24.7 (106)*****	32.7 (1 005)	**30.7 (20 553)*****	38.8 (225)	**26.8 (99)*****	**33.4 (883)***	**30.9 (17 986)*****
Higher education	16.4 (86)	11.4 (26)	15.5 (545)	13.6 (10 291)	18.4 (141)	**12.8 (55)***	16.0 (493)	**14.1 (9 408)*****	20.5 (119)	**13.2 (49)****	17.5 (462)	**14.6 (8 475)*****
Born in Sweden, % (n)	83.8 (438)	**76.4 (175)***	80.4 (2821)	87.6 (66 124)**	82.7 (633)	80.2 (344)	80.5 (2 474)	**87.7 (58 640)*****	84.3 (489)	86.2 (319)	80.5 (2 130)*	**87.7 (51 046)***
Income, mean (SD)	11 469 (8 677)	10 214 (7 958)	**10 253 (9 401)****	**10 481 (30 318)***	11 710 (8 118)	11 131 (7 780)	11 549 (8 803)	11 325 (17 293)	14 420 (12 394)	**12 129 (7 815)*****	13 541 (12 292)	**12 756 (15 869)****
Paid labour, % (n)	5.2 (27)	4.4 (*10*)	3.9 (138)	3.8 (2 850)	2.5 (19)	1.6 (7)	2.4 (75)	2.1 (1 425)	1.4 (*8*)	1.0 (5)	0.7 (19[b])	1.0 (592)
Hospital discharge diagnoses, % (n)												
Cancer	7.4 (39)	9.6 (22)	9.0 (314)	7.3 (5488)	13.2 (101)	12.4 (53)	12.9 (397)	**10.6 (7088)***	15.7 (91)	13.5 (50)	15.4 (408)	13.5 (7 851)
Neuropsychiatric diseases	6.1 (32)	**14.4 (33)*****	**10.1 (2353)****	8.4 (6357)	11.6 (89)	**16.1 (69)***	14.1 (432)	12.1 (8070)	16.4 (95)	15.4 (57)	18.3 (484)	16.5 (9 590)

For peer review only

For peer review only - http://bmjopen.bmj.com/site/about/guidelines.xhtml

Dementia	0.8 (4)	**3.1 (7)***	0.6 (20)	0.5 (357)	1.4 (11)	1.9 (8)	1.5 (45)	0.9 (604)	1.9 (11)	0.8 (3)	1.9 (50)	1.8 (1 019)
Cardiovascular diseases	23.7 (124)	**34.9 (80)****	26.4 (927)	26.7 (20 109)	36.9 (283)	**42.9 (184)***	39.4 (1 211)	38.3 (25 618)	47.2 (274)	43.5 (161)	49.5 (1 310)	49.9 (29 035)
Diabetes mellitus	4.4 (23)	4.8 (11)	4.9 (171)	4.9 (3 685)	8.7 (67)	9.6 (41)	8.1 (250)	7.3 (4 872)	9.0 (52)	7.3 (27)	9.6 (254)	9.8 (5 695)
Unipolar depression	0.8 (4)	0.0 (0)	1.5 (54)	1.4 (1 059)	1.6 (12)	**2.8 (12)****	2.7 (82)	2.1 (1 372)	3.4 (20)	3.5 (13)	4.0 (106)	2.8 (1 620)
Alcohol-related disorders	1.5 (8)	**5.2 (12)****	1.9 (65)	1.4 (1 056)	1.3 (10)	1.9 (8)	1.7 (51)	1.4 (906)	0.9 (5)	**2.7 (10)***	1.7 (45)	1.3 (743)
Ischemic heart diseases	10.3 (54)	12.7 (29)	11.5 (403)	11.5 (8 641)	15.1 (116)	16.3 (70)	16.7 (512)	16.3 (1 087)	17.2 (100)	17.0 (63)	19.1 (506)	19.9 (11 589)
Cerebrovascular diseases	2.7 (14)	**8.3 (19)*****	**5.4 (191)****	**5.9 (4 422)****	8.2 (63)	8.4 (36)	8.6 (264)	9.1 (6 054)	10.7 (62)	8.6 (32)	11.9 (315)	12.0 (6 973)
chronic obstructive pulmonary disease	2.3 (12)	**7.4 (17)*****	3.3 (115)	2.2 (1 619)	4.2 (32)	6.5 (28)	4.6 (140)	3.4 (2 252)	4.8 (28)	4.3 (16)	5.7 (151)	4.6 (2 682)
Osteoarthritis	6.3 (33)	**2.2 (5)***	4.7 (165)	5.8 (4 384)	9.5 (73)	**5.1 (22)****	7.8 (241)	9.7 (6 477)	14.7 (85)	**7.0 (26)*****	12.3 (325)	13.8 (8 029)

Comparisons made between participants and refusals, Gothenburg and Sweden.
Statistical analysis: to test differences between groups t test or χ2 test were used. *** p <0.001, ** p <0.01, * p <0.05; bolded numbers are significant at p <0.05; all other values are not significant.
[a] One individual is missing in the sociodemographic variables due personal identity number not found in the register.
[b] Information is missing on 3 individuals (all men)
Numbers in italics are not exact (true value within a 1-3 range) due to protection of the anonymity.

For peer review only

Table 3 continued. Characteristics of 85 and 88 years old participants and refusals, and same aged individuals in Gothenburg and Sweden in 2015 and 2018

	85-year olds in 2015				88-year olds in 2018			
	Participants	Refusals	Gothenburg	Sweden	Participants	Refusals	Gothenburg	Sweden
N	416[a]	256[b]	1 808	40 316	258	247[a]	1 321	29472
Sociodemographic factors								
Marital status, % (n)								
Married	37.8 (157)	31.5 (80)	35.4 (640)	36.8 (14 824)	26.7 (69)	29.7 (73)	30.0 (396)	29.6 (8 728)
Widowed	42.4 (176)	43.7 (111)	42.3 (764)	45.1 (18 172)	56.2 (145)	48.4 (119)	**49.3 (651)***	53.3 (15 700)
Divorced	14.5 (60)	18.1 (46)	16.8 (303)	12.3 (4 972)	12.8 (33)	16.3 (40)	15.6 (206)	11.8 (3 463)
Never married	5.3 (22)	6.7 (17)	5.6 (101)	5.8 (2 348)	4.3 (11)	5.7 (14)	5.1 (68)	5.4 (1 581)
Education, % (n)								
Elementary school	37.8 (157)	**55.1 (140)*****	**43.6 (788)***	**50.8 (20 499)*****	37.6 (97)	**48.4 (119)***	42.2 (558)	**49.7 (14 642)*****
Upper secondary school	40.0 (166)	**29.9 (76)****	**34.1 (617)***	**31.5 (12 687)*****	36.4 (94)	33.3 (82)	34.5 (456)	31.7 (9 347)
Higher education	20.7 (86)	**13.0 (33)***	18.5 (335)	**15.7 (6 323)****	23.6 (61)	**14.6 (36)***	19.1 (252)	**16.6 (4 879)****
Born in Sweden, % (n)	85.3 (354)	83.5 (212)	**80.9 (1 463)***	87.9 (35 445)	83.3 (215)	82.5 (203)	80.5 (1 064)	**88.0 (25 930)***
Income, mean (SD)	15 860 (9 759)	15 116 (20 451)	15 827 (22 202)	15 111 (21 828)	19 315 (25 829)	17 590 (20 007)	17 312 (20 615)	16 841 (25 586)
Paid labour, % (n)	N/A	N/A	N/A	N/A	N/A	N/A	N/A	N/A
Hospital discharge diagnoses, % (n)								
Cancer	20.2 (84)	18.0 (46)	20.1 (364)	17.9 (7 236)	19.8 (51)	20.2 (50)	20.9 (276)	19.1 (5 634)
Neuropsychiatric diseases	23.3 (97)	22.3 (57)	24.7 (446)	25.4 (1 0245)	24.8 (64)	**34.8 (86)***	27.7 (366)	29.0 (8 543)
Dementia	3.1 (13)	1.6 (4)	2.0 (36)	3.5 (1 413)	<4†	4.0 (10)	2.0 (27)	4.4 (1289)
Cardiovascular diseases	62.3 (259)	63.7 (163)	63.6 (1 150)	66.1 (26 660)	65.1 (168)	70.4 (174)	68.8 (909)	**72.2 (21 277)***
Diabetes mellitus	10.6 (44)	9.4 (24)	11.1 (200)	12.8 (5 176)	12.0 (31)	12.1 (30)	11.3 (149)	13.6 (4 019)

For peer review only - http://bmjopen.bmj.com/site/about/guidelines.xhtml

Unipolar depression	4.6 (19)	4.7 (12)	5.3 (95)	3.9 (1 555)	5.8 (15)	5.3 (13)	5.5 (73)	4.0 (1 191)
Alcohol-related disorders	<4†	<4†	1.2 (21)	1.0 (397)	0.0 (0)	<4†	1.1 (14)	0.8 (242)
Ischemic heart diseases	18.3 (76)	9.9 (51)	21.8 (395)	**24.1 (9 698)****	17.8 (46)	20.6 (51)	21.3 (282)	**25.1 (7 405)****
Cerebrovascular diseases	12.7 (53)	17.2 (44)	15.0 (272)	16.1 (6 503)	14.7 (38)	16.2 (40)	15.8 (209)	17.1 (5 044)
chronic obstructive pulmonary disease	4.8 (20)	4.7 (12)	5.6 (101)	6.0 (2 405)	5.4 (14)	2.8 (7)	4.9 (65)	6.1 (1 793)
Osteoarthritis	20.0 (83)	**13.3 (34)***	18.5 (335)	19.5 (7 854)	24.0 (62)	**15.4 (38)***	20.9 (276)	21.3 (6 268)

Comparisons made between participants and refusals, Gothenburg and Sweden.
Statistical analysis: to test differences between groups t test or χ2 test were used. *** *p* <0.001, ** *p* <0.01, * *p* <0.05; bolded numbers are significant at *p* <0.05; all other values are not significant.
[a] One individual is missing in the sociodemographic variables due personal identity number not found in the register.
[b] Two individuals are missing in the sociodemographic variables due personal identity number not found in the register.
N/A Not applicable as the cut of age for paid labour in Statistics Sweden registers is 84.
Numbers in italics are not exact (true value within a 1-3 range) due to protection of the anonymity.
†Less than four individuals. Exact number not presented to protect anonymity. Statistical comparison not performed.

For peer review only

For peer review only - http://bmjopen.bmj.com/site/about/guidelines.xhtml

Participants compared to refusals

Compared to refusals, participants had higher educational level and more often osteoarthritis at all examinations, less often neuropsychiatric disorders in 2000, 2005, and 2018, less often cardiovascular disorders in 2000 and 2005, were more often married in 2000 and 2005, had higher average income per month in 2009, had less often alcohol-related disorders in 2000 and 2009, were more often born in Sweden, had less often cerebrovascular diseases, chronic obstructive pulmonary disease, and dementia in 2000, and less often unipolar depression in 2005 (Table 3).

Participants compared to the target population of same-aged individuals in Gothenburg

The participants comprised 14.9% (2000), 24.9% (2005), 21.9% (2009), 23.0% (2015), and 19.5% (2018) of the target population, i.e. all same-aged individuals in the municipality of Gothenburg.

Compared to same-aged individuals in Gothenburg, participants had higher educational level in 2009 and 2015, had higher average income per month in 2000, were more often born in Sweden in 2009 and 2015, were more often widowed in 2018, and had less often cerebrovascular and neuropsychiatric disorders in 2000 (Table 3).

Participants compared to same-aged individuals in Sweden

Compared to same-aged individuals in Sweden, participants had higher educational level at all examinations, were less often born in Sweden at all examinations except in 2015, had higher average income per month in 2000 and 2009, were more often divorced in 2000 and 2005, had less often ischemic heart disease in 2015 and 2018, more often cancer in 2005, less often cardiovascular diseases in 2018, and less often cerebrovascular diseases in 2000 (Table 3).

Discussion

We examined a birth cohort from Gothenburg, Sweden, at repeated cross-sectional examinations from age 70 to 88, and found that response rate declined with age. Participants were examined regarding three levels of representativeness: in relation to refusals, in relation to same-aged

For peer review only - http://bmjopen.bmj.com/site/about/guidelines.xhtml

individuals in Gothenburg (the target population), and in relation to same-aged individuals in Sweden. We found a number of differences in sociodemographic factors and diseases in registry data between participants and refusals, as well as a lower mortality rate among participants. Participants were similar regarding a number of factors to the target population of all same-aged individuals in Gothenburg, while they differed compared to all same-aged individuals in Sweden.

This study has both strengths and limitations. The strengths of this study include five time-points of cross-sectional health examinations, the use of register data to compare participants not only with refusals, but also with same-aged individuals from Gothenburg and same-aged individuals from the entire country. There are also several limitations. First, the same individuals are included at repeated examinations and might thus be influenced by the Hawthorne effect, i.e. they might change behaviours as a result of their participation, resulting in increased differences between participants and refusals (11). Second, participants make up 15-25% of the total comparison group of individuals in Gothenburg. This made the comparison group more similar to participants. On the other hand, if we excluded participants, the comparison group would have been less representative of same-aged individuals in Gothenburg. Third, using register data to compare participants with the target population might miss important differences. A previous study found only minor differences in disease burden between participants and non-participants when using registers, but large differences when using clinical and biochemical variables from a baseline examination conducted 20 years earlier (12). It should also be noted that the sensitivity of disorders in the NPR varies, with higher sensitivity for more severe disorders and less for milder disorders (9, 13). Fourth, in some years the examinations were delayed due to logistic issues, creating a higher mean age in the population examined than was targeted. This increased the number of individuals who died or developed disorders before the examination, a bias which becomes more important with age. Finally, a small group was excluded due to either not speaking Swedish (1.6% to 4.1%) or not being traceable (0.5%

For peer review only - http://bmjopen.bmj.com/site/about/guidelines.xhtml

to 3.6%). These groups were however small, and the exclusion did probably not affect the main results.

The participation rate was initially 70% at age 70, declined to 61-64% at ages 75-85, and dropped to 51% at age 88. Response rates between 50-70% has previously been reported in similar Swedish population-based studies of adults aged 65 years and older (14), with inicdations that non-response was higher among the oldest population. Possible reasons for declining response rate with advancing age may be participation exhaustion, an age effect, or a period effect. Participation exhaustion might occur due to a high number of extensive examinations. To minimize this effect, we offered to perform the examination at the participant's homes, or to split up the examinations at different occasions (15). However, the response rates in our previous studies rather support an age effect where the response rate among 70-year-olds born 1944 and 1930 was around 70% (4), and among 85-year-olds born 1923-24 and 1930 around 60% (6). Other explanations for an age effect, reported also by others (16-18), include cognitive impairment (17, 18), frailty, and illness (17), which all increase with age. Population-based studies in older populations might thus be less representative with increasing age. Support for a period effect is suggested by the declining response rates in research studies over the last decades (1). For example, the first H70 examination conducted in 1971 on 70-years-olds had a response rate of 85% (5). At that examination, participants and non-participants were very similar, in contrast to the examination of 70-year-olds in our study. Another example is a Finnish health survey (19) conducted 1987-2012. They reported a declining participation rate over time in all socioeconomic groups, but a steeper decline among those with low educational level. This resulted in increased differences between participants and non-participants (19). Reasons for period effects include an overall decrease in social participation, increased complexity of modern life, lower trust in society, science, and research, increased burden due to increasing number of invitations to participate in studies, and increasingly demanding research

For peer review only

For peer review only - http://bmjopen.bmj.com/site/about/guidelines.xhtml

projects (7). This increases the risk of healthy-responder bias, making the results from population-based studies less generalizable and cohort comparisons more difficult to perform.

Our finding that participants in general were healthier, and had higher education and income than refusals are in line with a number of population-based studies. For example, participants compared to refusals are reported to be younger (16-18), more often cohabiting or married (16, 20-22) and born in the study country or in neighbouring countries (20, 21), have higher income (21), educational level (20, 22), socioeconomic status (12), better health status (12, 23), less often a diagnosis in hospital discharge registers (12, 16, 20), and lower mortality rates (12, 16). Among older persons, participants reportedly have less cognitive impairment (17, 18), illness, and frailty (17). Interestingly, refusals had less often osteoarthritis in the NPR compared to participants at all examinations, in contrast to what was observed for most other disorders. Osteoarthritis is a disorder with a fairly large proportion of undiagnosed cases (24). It is thus possible that those not seeking medical attention or receiving specialized care for osteoarthritis are also less prone to participate in health examination studies.

We also examined differences between participants and the total population of same-aged individuals in Gothenburg, i.e. the target population, to get a better appreciation of external validity than is achieved by only examining differences compared to refusals. Our finding that participants were more similar to its target population than to refusals, especially at younger ages, is in line with previous studies (12, 22). However, there were some differences. Participants had higher educational level and were more often born in Sweden at ages 79 and 85, had less often cerebrovascular and neuropsychiatric disorders and higher mean income at age 70, and were more often widowed at age 88. The proportion being excluded due to not speaking Swedish was larger at ages 79 and 85, compared to the other examination years. This might explain why participants compared to same-aged individuals in Gothenburg were more often born in Sweden at these ages.

For peer review only - http://bmjopen.bmj.com/site/about/guidelines.xhtml

There were more differences between participants and same-aged individuals in Sweden. This is not surprising, as Gothenburg is one of Sweden's largest cities, and a university city. In addition, it has a higher proportion of individuals born outside Sweden, and higher educational level than rural areas of Sweden. This illustrates the difficulty to generalize findings from a local target population to even a relatively small and homogenous population, as Sweden.

We found no differences in response rates between women and men, in contrast to other population-based studies, which report higher response rates among women (16, 20, 22, 25). However, in two of these studies there were no sex differences at higher ages (16, 25), and the other two only included individuals aged below 64 and 54 years, respectively (20, 22). Some studies report that sex differences in response rates diminish with age (22, 26).

Conclusion

In conclusion, our data show that participants were similar to the target population of Gothenburg. However, participants differed most compared to refusals, and to a certain extent to same-aged individuals in Sweden, indicating that the studies might underestimate the burden of disease. These results contribute with uniqe and up-to-date knowledge on participation bias in these high age groups, and shows the importance to have different comparison groups when assessing representativeness. This finding is readily transferable to similar studies, and since population-based studies on older adults contribute with information on disease distribution and knowledge of risk-disease associations, it is important to be aware of potential limitations in the representativeness that might hamper generalizability.

For peer review only - http://bmjopen.bmj.com/site/about/guidelines.xhtml

Acknowledgments

Funding

The study was financed by grants from the Swedish state under the agreement between the Swedish government and the county councils, the ALF-agreement (ALF 716681); Stena Foundation; Swedish Research Council (11267, 2005-8460, 2007-7462, 2012-5041, 2015-02830, 2019-01096, 2013-8717, NEAR 2017-00639); Swedish Research Council for Health, Working Life and Wellfare (2004-0145, 2006-0596, 2008-1111, 2010-0870, 2013-1202, 2018-00471, 2001-2646, 2003-0234, 2004-0150, 2006-0020, 2008-1229, 2012-1138, AGECAP 2013-2300, 2013-2496); Konung Gustaf V:s och Drottning Victorias Frimurarestiftelse; Hjärnfonden (FO2014-0207, FO2016-0214, FO2018-0214, FO2019-0163, FO2020-0235); Alzheimerfonden (AF-554461, AF-647651, AF-743701, AF-844671, AF-930868, AF-940139); Eivind och Elsa K:son Sylvans stiftelse; The Alzheimer's Association Zenith Award (ZEN-01-3151); The Alzheimer's Association Stephanie B. Overstreet Scholars (IIRG-00-2159); The Bank of Sweden Tercentenary Foundation; Stiftelsen Söderström-Königska Sjukhemmet; Stiftelsen för Gamla Tjänarinnor; Handlanden Hjalmar Svenssons Forskningsfond.

Author SK was financed by grants from the Swedish state under the agreement between the Swedish government and the county councils, the ALF-agreement (ALFGBG-81392, ALF GBG-771071); the Alzheimerfonden (AF-842471, AF-737641); The Swedish Research Council (2019-02075); Stiftelsen Demensfonden; Stiftelsen Hjalmar Svenssons Forskningsfond; and Stiftelsen Wilhelm och Martina Lundgrens vetenskapsfond. Author SS was financed by grants from the ALF-agreement (ALFGBG-637271); and the Sahlgrenska Academy Homecoming Fellowship (Dnr V2012/294).

Disclosure of interest

The authors have no relevant financial or non-financial interests to disclose.

Author Contributions

For peer review only

Hanna Wetterberg, Lina Rydén, and Ingmar Skoog had full access to all the data in the study and take responsibility for the integrity of the data and the accuracy of the data analysis. Concept and design: Wetterberg, Rydén, and Skoog. Acquisition, analysis, or interpretation of data: All authors. Drafting of the manuscript: Wetterberg, Rydén, and Skoog. Statistical analysis: Wetterberg and Rydén. Principal investigator: Ingmar Skoog. Critical revision of the manuscript for important intellectual content: All authors.

Additional contributors

The authors would like to thank all participants of the H70 studies in Gothenburg, and the research group members for their cooperation in data collection and management. We also acknowledge seminal contributions to this study by the late Dr. Svante Östling.

Ethics approval

The H70 study was conducted according to the ethical standards of the institutional committee and with the 1964 Helsinki Declaration and its later amendments or comparable ethical standards. The Regional Ethical Review Board approved the H70 studies in Gothenburg (approval numbers: 240800/S227-00, 041104/T453-04, 090309/075-09, 270415/131-15, and 230418/278-18). The Swedish Ethical Review Authority has approved the publication of aggregated data on the individuals that declined participation in the study (approval numbers: 170620/2020-02181, 170620/2020-02188).

Consent to participate

Informed consent was obtained from all participants included in the study. In cases of dementia, informed consent was obtained from a close relative.

Availability of data and material

All data and analyses generated during the current study are available from the corresponding author on reasonable request.

For peer review only

References

1. Galea S, Tracy M. Participation Rates in Epidemiologic Studies. Ann Epidemiol. 2007;17(9):643-53.

2. Levin KA. Study design III: Cross-sectional studies. Evidence-Based Dentistry. 2006;7:24-5.

3. Mindell JS, Giampaoli S, Goesswald A, Kamtsiuris P, Mann C, Männistö S, Morgan K, Shelton NJ, Verschuren WM, Tolonen H. Sample selection, recruitment and participation rates in health examination surveys in Europe--experience from seven national surveys. BMC Med Res Methodol. 2015;15:78.

4. Rydberg Sterner T, Ahlner F, Blennow K, Dahlin-Ivanoff S, Falk H, Havstam Johansson L, Hoff M, Holm M, Horder H, Jacobsson T, Johansson B, Johansson L, Kern J, Kern S, Machado A, Mellqvist Fassberg M, Nilsson J, Ribbe M, Rothenberg E, Ryden L, Sadeghi A, Sacuiu S, Samuelsson J, Sigstrom R, Skoog J, Thorvaldsson V, Waern M, Westman E, Wetterberg H, Zetterberg H, Zetterberg M, Zettergren A, Ostling S, Skoog I. The Gothenburg H70 Birth cohort study 2014-16: design, methods and study population. Eur J Epidemiol. 2019;34(2):191-209.

5. Rinder L, Roupe S, Steen B, Svanborg A. Seventy-year-old people in Gothenburg. A population study in an industrialized Swedish city. Acta Med Scand. 1975;198(5):397-407.

6. Skoog I, Borjesson-Hanson A, Kern S, Johansson L, Falk H, Sigstrom R, Ostling S. Decreasing prevalence of dementia in 85-year olds examined 22 years apart: the influence of education and stroke. Sci Rep. 2017;7.

7. Morton SM, Bandara DK, Robinson EM, Carr PE. In the 21st Century, what is an acceptable response rate? Aust N Z J Public Health. 2012;36(2):106-8.

8. Bengtsson C, Blohme G, Hallberg L, Hallstrom T, Isaksson B, Korsan-Bengtsen K, Rybo G, Tibblin E, Tibblin G, Westerberg H. The study of women in Gothenburg 1968-1969--a population study. General design, purpose and sampling results. Acta Med Scand. 1973;193(4):311-8.

9. Ludvigsson JF, Andersson E, Ekbom A, Feychting M, Kim J-L, Reuterwall C, Heurgren M, Olausson PO. External review and validation of the Swedish national inpatient register. BMC Public Health. 2011;11:450-.

10. von Elm E, Altman DG, Egger M, Pocock SJ, Gotzsche PC, Vandenbroucke JP, Initiative S. Strengthening the Reporting of Observational Studies in Epidemiology (STROBE) statement: guidelines for reporting observational studies. BMJ. 2007;335(7624):806-8.

11. McCambridge J, Witton J, Elbourne DR. Systematic review of the Hawthorne effect: New concepts are needed to study research participation effects. J Clin Epidemiol. 2014;67(3):267-77.

12. Drivsholm T, Eplov LF, Davidsen M, Jørgensen T, Ibsen H, Hollnagel H, Borch-Johnsen K. Representativeness in population-based studies: A detailed description of non-response in a Danish cohort study. Scandinavian Journal of Public Health. 2006;34(6):623-31.

13. Ryden L, Sigstrom R, Nilsson J, Sundh V, Erhag HF, Kern S, Waern M, Ostling S, Wilhelmson K, Skoog I. Agreement between self-reports, proxy-reports and the National Patient Register regarding diagnoses of cardiovascular disorders and diabetes mellitus in a population-based sample of 80-year-olds. Age Ageing. 2019;48(4):513-8.

14. Lagergren M, Fratiglioni L, Hallberg IR, Berglund J, Elmstahl S, Hagberg B, Holst G, Rennemark M, Sjolund BM, Thorslund M, Wiberg I, Winblad B, Wimo A. A longitudinal study integrating population, care and social services data. The Swedish National study on Aging and Care (SNAC). Aging Clin Exp Res. 2004;16(2):158-68.

For peer review only

15. Lissner L, Skoog I, Andersson K, Beckman N, Sundh V, Waern M, Zylberstein DE, Bengtsson C, Bjorkelund C. Participation bias in longitudinal studies: experience from the Population Study of Women in Gothenburg, Sweden. Scand J Prim Health Care. 2003;21(4):242-7.

16. Bergholdt HKM, Bathum L, Kvetny J, Rasmussen DB, Moldow B, Hoeg T, Jemec GBE, Berner-Nielsen H, Nordestgaard BG, Ellervik C. Study design, participation and characteristics of the danish general suburban population study. Dan Med J. 2013;Sep;60(9):A469.

17. Chatfield MD, Brayne CE, Matthews FE. A systematic literature review of attrition between waves in longitudinal studies in the elderly shows a consistent pattern of dropout between differing studies. J Clin Epidemiol. 2005;58(1):13-9.

18. Matthews FE, Chatfield M, Brayne C, Cfas M. An investigation of whether factors associated with short-term attrition change or persist over ten years: data from the Medical Research Council Cognitive Function and Ageing Study (MRC CFAS). BMC Public Health. 2006;6.

19. Reinikainen J, Tolonen H, Borodulin K, Harkanen T, Jousilahti P, Karvanen J, Koskinen S, Kuulasmaa K, Mannisto S, Rissanen H, Vartiainen E. Participation rates by educational levels have diverged during 25 years in Finnish health examination surveys. Eur J Public Health. 2018;28(2):237-43.

20. Lundberg I, Damström Thakker K, Hällström T, Forsell Y. Determinants of non-participation, and the effects of non-participation on potential cause-effect relationships, in the PART study on mental disorders. Soc Psychiatry Psychiatr Epidemiol. 2005;40(6):475-83.

21. Bergstrand R, Vedin A, Wilhelmsson C, Wilhelmsen L. Bias Due to Non-Participation and Heterogenous Subgroups in Population Surveys. J Chronic Dis. 1983;36(10):725-8.

22. Korkeila K, Suominen S, Ahvenainen J, Ojanlatva A, Rautava P, Helenius H, Koskenvuo M. Non-response and related factors in a nation-wide health survey. Eur J Epidemiol. 2001;17(11):991-9.

23. Knudsen AK, Hotopf M, Skogen JC, Overland S, Mykletun A. The Health Status of Nonparticipants in a Population-based Health Study The Hordaland Health Study. Am J Epidemiol. 2010;172(11):1306-14.

24. Turkiewicz A, Gerhardsson de Verdier M, Engström G, Nilsson PM, Mellström C, Lohmander LS, Englund M. Prevalence of knee pain and knee OA in southern Sweden and the proportion that seeks medical care. Rheumatology. 2014;54(5):827-35.

25. Krokstad S, Langhammer A, Hveem K, Holmen TL, Midthjell K, Stene TR, Bratberg G, Heggland J, Holmen J. Cohort Profile: the HUNT Study, Norway. Int J Epidemiol. 2013;42(4):968-77.

26. Laaksonen M, Aittomäki A, Lallukka T, Rahkonen O, Saastamoinen P, Silventoinen K, Lahelma E. Register-based study among employees showed small nonparticipation bias in health surveys and check-ups. J Clin Epidemiol. 2008;61(9):900-6.

For peer review only

For peer review only - http://bmjopen.bmj.com/site/about/guidelines.xhtml

Figure captions

Fig. 1 Sample flow chart describing the five cross-sectional examination waves

Figure legend

Rr = Response rate (participants/ eligble sample)
[1]All 70-year-olds living in Gothenburg and born in 1930 on days 3, 6, 12, 18, 21, 24, or 30
[2]All 75-year-olds living in Gothenburg and born in 1930 on days 2, 3, 5, 6, 11, 12, 16, 18, 20, 21, 24, 27 (in January-May), or 30
[3]n=386 had previously been examined at age 70.
[4]Included only individuals that had been invited to the 2000 or 2005 examination
[5]Misclassified as not living in Gothenburg and therefore not invited
[6] n=539 had previously been examined (n=307 at age 70, and n=511 at age 75, and n=279 in both)
[7]All 85-year-olds living in Gothenburg and born in 1930 on days 2, 3, 5, 6, 11, 12, 16, 18, 20, 21, 24, 27, or 30
[8]Misclassified as not living in Gothenburg and therefore not invited
[9]n=370 had previously been examined (n=199 at age 70, n=338 at age 75, n=337 at age 79, and n=174 in all three)
[10]All 88-year-olds living in Gothenburg and born in 1930 on days 2, 3, 5, 6, 11, 12, 16, 18, 20, 21, 24, 27, or 30
11n=247 had previously been examined (n=120 at age 70, n=205 at age 75, n=207 at age 79, n=224 at age 85, and n=102 in all four)

For peer review only

For peer review only - http://bmjopen.bmj.com/site/about/guidelines.xhtml

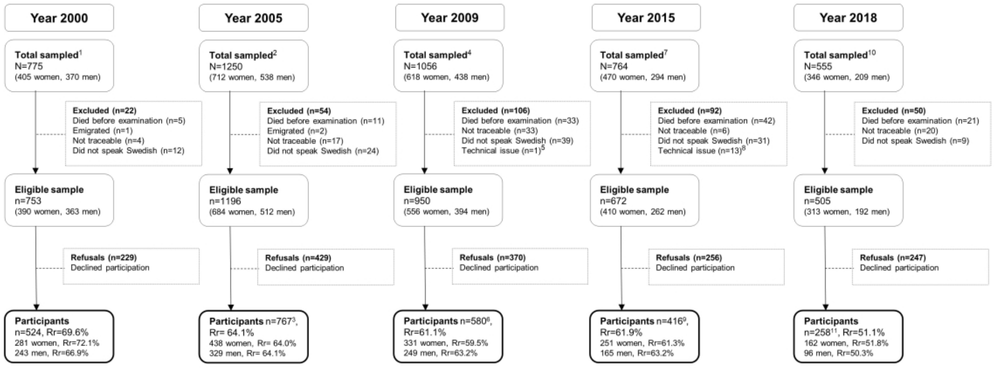

Figure captions.
Fig. 1 Sample flow chart describing the five cross-sectional examination waves.
Figure legend.
Rr = Response rate (participants/ eligble sample).
1 All 70-year-olds living in Gothenburg and born in 1930 on days 3, 6, 12, 18, 21, 24, or 30.
2 All 75-year-olds living in Gothenburg and born in 1930 on days 2, 3, 5, 6, 11, 12, 16, 18, 20, 21, 24, 27 (in January-May), or 30.
3 n=386 had previously been examined at age 70.
4 Included only individuals that had been invited to the 2000 or 2005 examination.
5 Misclassified as not living in Gothenburg and therefore not invited.
6 n=539 had previously been examined (n=307 at age 70, and n=511 at age 75, and n=279 in both).
7 All 85-year-olds living in Gothenburg and born in 1930 on days 2, 3, 5, 6, 11, 12, 16, 18, 20, 21, 24, 27, or 30.
8 Misclassified as not living in Gothenburg and therefore not invited.
9 n=370 had previously been examined (n=199 at age 70, n=338 at age 75, n=337 at age 79, and n=174 in all three).
10 All 88-year-olds living in Gothenburg and born in 1930 on days 2, 3, 5, 6, 11, 12, 16, 18, 20, 21, 24, 27, or 30.
11 n=247 had previously been examined (n=120 at age 70, n=205 at age 75, n=207 at age 79, n=224 at age 85, and n=102 in all four).

338x190mm (300 x 300 DPI)

Additional file 1

BMJ Open

Representativeness in population-based studies of older adults – Five waves of cross-sectional examinations in the Gothenburg H70 Birth Cohort Study

Hanna Wetterberg, MSc[ab]*, Lina Rydén, MD[abc]*, Felicia Ahlner, MSc[ab], Hanna Falk Erhag, RN, PhD[ab], Pia Gudmundsson, PhD[ab], Xinxin Guo, MD, PhD[ab],[d], Erik Joas, PhD[ab], Lena Johansson, RN, PhD[ab], Silke Kern, MD, PhD[abc], Madeleine Mellqvist Fässberg, PhD[ab], Jenna Najar, MD, PhD[abc], Mats Ribbe, MD, PhD[ab], Therese Rydberg Sterner, PhD[abe], Jessica Samuelsson, MSc[ab], Simona Sacuiu MD, PhD[abc], Robert Sigström, MD, PhD[abc], Johan Skoog, PhD[ab], Margda Waern, MD, PhD[abf], Anna Zettergren, PhD[ab], Ingmar Skoog, MD, PhD[abc]

[*] HW and LR are joint first authors

[a]Neuropsychiatric Epidemiology Unit, Department of Psychiatry and Neurochemistry, Institute of Neuroscience and Physiology, Sahlgrenska Academy, at the University of Gothenburg, Sweden; [b]Centre for Ageing and Health (AGECAP) at the University of Gothenburg, Sweden; [c]Region Västra Götaland, Sahlgrenska University Hospital, Psychiatry, Cognition and Old Age Psychiatry Clinic, Gothenburg, Sweden; [d]Region Västra Götaland, Sahlgrenska University Hospital, Department of Mood disorders, Gothenburg, Sweden; [e]Aging Research Center, Department of Neurobiology, Care Sciences and Society, Karolinska Institutet and Stockholm University, Stockholm, Sweden; [f]Region Västra Götaland, Department of Psychiatry, Psychotic Disorders, Sahlgrenska University Hospital, Mölndal, Sweden

Corresponding author:

Hanna Wetterberg, hanna.wetterberg@gu.se

For peer review only

Table s1*. *Examinations included at each wave

	2000	2005	2009	2015	2018
General examinations					
General health interview	YES	YES	YES	YES	YES
Psychiatric interview	YES	YES	YES	YES	YES
Cognitive examination[a]	YES [g]	YES	YES	YES	YES
Physical examination by medical doctor	YES	YES	NO	NO	NO
Physical examination[b]	YES	YES	YES	YES	YES
Social factors interview	YES	YES	YES	YES	YES
Physiotherapy examination	YES	YES	YES	YES	YES
Functional ability[d]	YES	YES	YES	YES	YES
Periodic limb movement	YES[h]	YES	NO	NO	NO
Audiological examination	YES	YES	NO	YES[i]	NO
Dental examination	YES	YES	NO	NO	NO
Blood sampling[e]	YES	YES	YES	YES	YES
Genetic analyses	YES	YES	YES	YES	YES
Urine sampling	YES	YES	NO	NO	NO
Personality tests (Cesarec Marke Personality Scheme and Eysenck)	NO	YES	YES	YES	NO
Additional examinations					
Close informant interview	YES	YES	YES	YES	YES
Dietary examination	YES	NO	NO	YES	NO
Body composition examination					
Dual-energy X-ray absorptiometry	NO	YES[h]	YES[h]	YES[h]	YES[h]
Bioelectrical impedance spectroscopy	NO	YES[h]	YES[h]	YES[h]	YES[h]
Brain imaging examination					
Magnetic resonance imaging	NO	NO	NO	YES	YES
Computed tomography	YES	NO	YES	YES	YES
Cerebrospinal fluid sampling[f]	NO	NO	YES	YES	YES
Extended audiological examination	NO	NO	NO	YES[h]	YES[h]

[a] Including clinical screening tests as well as more extensive psychometric tests
[b] Including examinations such as blood pressure, anthropometry, visual acuity, neurological status, electrocardiogram, lung function examinations, Bioelectrical Impedance Analysis,
[d] Measured as Activities of Daily Living (ADL), and instrumental activities of daily living (IADL) similar to Katz and Lawton ADL, and from 2015 also the Barthel index.
[e] Used for e.g. clinical chemistry analyses.
[f] Collected by lumbar puncture.
[g] Half of the sample performed an extended psychometric test, which in later cohorts were performed by all participants.
[h] A subsample was invited.
[i] Performed on those examined at the research clinic and a subsample of those examined at home.

For peer review only

Table s2. The hospital discharge diagnoses included

	ICD 9	ICD 10
Cancer	140-208	C00-C97
Neuropsychiatric disorders including dementia disorders	290-319, 324-359<br>290, 291C, 294, 331	F01-F99, G06-G98<br>F01, F02, G30, G31]
Cardiovascular diseases	390-459	I00-I99
Diabetes mellitus	250	E10-E14
Unipolar depression	296B, 311	F32-F33
Alcohol-related disorders	291, 303, 305A	F10
Ischemic heart disease	410-414	I20-I25
Cerebrovascular diseases	430-438	I60-I69
Chronic obstructive pulmonary disease	490-492, 495-496	J40-J44
Osteoarthritis	715	M15-M19

For peer review only

Table s3. Comparison between examinations of excluded individuals

Total excluded (includes did not speak Swedish, died after sampling, and not traceable)					
	**2000** 2.8% (22/775)	**2005** 4.3% (54/1250)	**2009** 10.0% (106/1056)	**2015** 12.0% (92/764)	**2018** 9.0% (50/555)
**2000**, 2.8% (22/775)	-	p=0.088	**p<0.001**	**p<0.001**	**p<0.001**
**2005**, 4.3% (54/1250)		-	**p<0.001**	**p<0.001**	**p<0.001**
**2009**, 10.0% (106/1056)			-	p=0.175	p=0.507
**2015**, 12.0% (92/764)				-	p=0.079
**2018**, 9.0% (50/555)					-
**Did not speak Swedish**					
	**2000** 1.6% (12/775)	**2005** 1.9% (24/1250)	**2009** 3.7% (39/1056)	**2015** 4.1% (31/764)	**2018** 1.6% (9/555)
**2000,**1.6% (12/775)	-	p=0.538	**p=0.006**	**p=0.003**	p=0.916
**2005**, 1.9% (24/1250)		-	**p=0.009**	**p=0.001**	p=0.867
**2009**, 3.7% (39/1056)			-	p=0.690	**p=0.020**
**2015**, 4.1% (31/764)				-	**p=0.011**
**2018**, 1.6% (9/555)					-
**Died after sampling**					
	**2000** 0.7% (5/775)	**2005** 0.9% (11/1250)	**2009** 3.1% (33/1056)	**2015** 5.5% (42/764)	**2018** 3.8% (21/555)
**2000**, 0.7% (5/775)	-	p=0.562	**p<0.001**	**p<0.001**	**p<0.001**
**2005**, 0.9% (11/1250)		-	**p<0.001**	**p<0.001**	**p=0.017**
**2009**, 3.1% (33/1056)			-	**p=0.012**	p=0.485
**2015**, 5.5% (42/764)				-	p=0.150
**2018**, 3.8% (21/555)					-
**Not traceable**					
	**2000** 0.5% (4/775)	**2005** 1.4% (17/1250)	**2009** 3.1% (33/1056)	**2015** 0.8% (6/764)	**2018** 3.6% (20/555)
**2000**, 0.5% (4/775)	-	p=0.074	**p<0.001**	p=0.544	**p<0.001**
**2005**, 1.4% (17/1250)		-	**p=0.004**	p=0.324	**p<0.001**
**2009**, 3.1% (33/1056)			-	**p=0.001**	p=0.609
**2015**, 0.8% (6/764)				-	**p<0.001**
**2018**, 3.6% (20/555)					-
Statistical analysis: to test differences between groups χ2 or Fisher's exact test were used. Bolded numbers are significant at p <0.05.					

For peer review only

Table s4. Characteristics of 70 years old participants and refusals, and same aged individuals in Gothenburg and Sweden in 2000, stratified by sex

	**Women**				**Men**			
	**Participants**	**Refusals**	**Gothenburg**	**Sweden**	**Participants**	**Refusals**	**Gothenburg**	**Sweden**
N	281 (53.6)	109 (47.6)	1 965 (56.0)	40 655 (53.9)	243[a] (46.4)	120 (52.4)	1 543 (44.0)	34 813 (46.1)
**Sociodemographic factors**								
Marital status, % (n)								
Married	46.3 (130)	49.5 (54)	49.0 (963)	**55.2 (22 425)****	74.8 (181)	**56.7 (68)*****	**65.1 (1 005)****	69.7 (24 272)
Widowed	24.9 (70)	22.0 (24)	23.2 (455)	24.4 (9 937)	2.9 (7)	**8.3 (10)***	**6.4 (98)***	**6.8 (2 354)***
Divorced	23.1 (65)	19.3 (21)	21.3 (419)	**14.5 (5 899)*****	14.9 (36)	15.0 (18)	16.9 (260)	13.4 (4 677)
Never married	5.7 (16)	9.2 (10)	6.5 (128)	5.9 (2 394)	7.4 (18)	**20.0 (24)*****	11.7 (180)	10.1 (3 510)
Education, % (n)								
Elementary school	54.4 (153)	58.7 (64)	51.5 (1 012)	55.3 (22 497)	41.0 (*100*)	**58.0 (*69*)****	43.2 (666)	**52.3 (18 197)*****
Upper secondary school	31.7 (89)	22.0 (24)	30.1 (591)	29.9 (12 150)	36.4 (88)	32.5 (39)	36.2 (558)	31.2 (10 877)
Higher education	12.1 (34)	15.6 (17)	14.0 (276)	12.5 (5 088)	21.5 (52)	**7.5 (9)*****	17.4 (269)	**14.9 (5 203)****
Born in Sweden, % (n)	83.6 (235)	75.2 (82)	80.0 (1 572)	86.2 (35 049)	83.9 (203)	77.5 (93)	80.9 (1 249)	**89.3 (31 075)****
Income, mean (SD)	8970 (5098)	8270 (6637)	8670 (5190)	8520 (7320)	14370 (10824)	**11980 (8646)***	**12270 (1403)****	**12770 (43821)***
Paid labour, % (n)	3.6 (10)	3.0 (*3*)	2.8 (55)	2.2 (875)	7.0 (17)	5.8 (7)	5.4 (83)	5.7 (1 975)
**Hospital discharge diagnoses,** % (n)								
Cancer	6.8 (19)	9.2 (10)	9.3 (182)	7.6 (3087)	8.2 (20)	10.0 (12)	8.6 (132)	6.9 (2401)
Neuropsychiatric diseases	5.7 (16)	7.3 (8)	9.0 (176)	7.8 (3161)	6.6 (16)	**20.8 (25)*****	**11.5 (177)***	9.2 (3196)
Dementia	1.1 (3)	1.8 (2)	0.6 (11)	0.5 (190)	0.4 (1)	**4.2 (5)***	0.6 (9)	0.5 (167)
Cardiovascular diseases	18.1 (51)	**29.4 (32)***	21.0 (412)	21.3 (8654)	30.0 (73)	40.0 (48)	33.4 (515)	32.9 (11455)
Diabetes mellitus	2.8 (8)	3.7 (4)	3.5 (69)	3.9 (1580)	6.2 (15)	5.8 (7)	6.6 (102)	6.1 (2105)
Unipolar depression	1.1 (3)	0 (0)	1.8 (36)	1.6 (659)	0.4 (1)	0 (0)	1.2 (18)	1.2 (400)
Alcohol-related disorders	0.7 (2)	1.8 (2)	0.8 (15)	0.6 (240)	2.5 (6)	**8.3 (10)***	3.2 (50)	2.3 (816)
Ischemic heart diseases	6.4 (18)	7.3 (8)	7.0 (137)	7.2 (2929)	14.8 (36)	17.5 (21)	17.2 (266)	16.4 (5712)
Cerebrovascular diseases	3.9 (11)	4.6 (5)	4.2 (83)	4.4 (1782)	1.2 (3)	**11.7 (14)*****	**7.0 (108)*****	**7.6 (2640)*****
chronic obstructive pulmonary disease	2.5 (7)	**8.3 (9)****	3.4 (66)	2.0 (808)	2.1 (5)	**6.7 (8)***	3.2 (49)	2.3 (811)

For peer review only

Osteoarthritis	7.8 (22)	2.8 (3)	5.3 (104)	6.1 (2479)	4.5 (11)	1.7 (2)	4.0 (61)	5.5 (1905)

Comparisons made between participants and refusals, Gothenburg and Sweden.
Statistical analysis: to test differences between groups t test or $\chi 2$ test were used. *** $p < 0.001$, ** $p < 0.01$, * $p < 0.05$; bolded numbers are significant at $p < 0.05$; all other values are not significant.
[a] One individual is missing in the sociodemographic variables due personal identity number not found in the register.
Numbers in italics are not exact (true value within a 1-3 range) due to protection of the anonymity.

For peer review only

Table s5. Characteristics of 75 years old participants and refusals, and same aged individuals in Gothenburg and Sweden in 2005, stratified by sex

	**Women**				**Men**			
	**Participants**	**Refusals**	**Gothenburg**	**Sweden**	**Participants**	**Refusals**	**Gothenburg**	**Sweden**
N (%)	438 (57.1)	246 (57.3)	1 768 (57.5)	37 075 (55.5)	329 [a] (42.9)	183 (42.7)	1 307 (42.5)	29 780 (44.5)
**Sociodemographic factors**								
Marital status, % (n)								
Married	41.8 (183)	41.9 (103)	42.9 (759)	46.2 (17 134)	71.9 (235)	**55.7 (102)*** **	**65.2 (852)***	67.5 (20 104)
Widowed	33.1 (145)	26.8 (66)	30.3 (535)	33.9 (12 581)	8.0 (26)	**14.8 (27)***	9.6 (126)	10.5 (3 134)
Divorced	20.1 (88)	25.6 (63)	20.7 (366)	**14.2 (5 282)*****	13.1 (43)	16.4 (30)	14.8 (194)	12.7 (3 769)
Never married	5.0 (22)	5.7 (14)	6.1 (108)	5.6 (2 078)	7.0 (23)	**13.1 (24)***	10.3 (135)	9.3 (2 773)
Education, % (n)								
Elementary school	49.0 (*213*)	**59.3 (146)****	51.5 (910)	**54.7 (20 280)***	38.8 (127)	**57.9 (106)*****	42.6 (557)	**51.4 (15 304)*****
Upper secondary school	35.8 (157)	**23.6 (58)*****	**30.3 (535)***	**30.2 (11 190)***	37.0 (121)	**26.2 (48)***	36.0 (470)	**31.4 (9 363)***
Higher education	15.1 (66)	13.0 (32)	14.1 (250)	12.9 (4 772)	22.9 (75)	**12.6 (23)****	18.6 (243)	**15.6 (4 636)*****
Born in Sweden, % (n)	83.6 (366)	80.1 (197)	80.4 (1 421)	86.4 (32 025)	81.7 (267)	80.3 (147)	80.6 (1 053)	**89.4 (26 615)*****
Income, mean (SD)	10240 (8273)	9920 (7564)	10010 (7157)	9650 (8295)	13680 (7477)	12760 (7789)	13630 (10275)	13410 (24038)
Paid labour, % (n)	1.6 (7)	1.0 (2)	1.8 (31)	1.1 (409)	3.7 (12)	2.7 (5)	3.4 (44)	3.4 (1 016)
**Hospital discharge diagnoses, %** (n)								
Cancer	12.1 (53)	11.0 (27)	12.7 (225)	10.4 (3872)	14.6 (48)	14.2 (26)	13.2 (172)	**10.8 (3216)***
Neuropsychiatric diseases	12.3 (54)	14.2 (35)	12.3 (218)	11.2 (4157)	10.6 (35)	**18.6 (34)***	**16.4 (214)****	13.1 (3913)
Dementia	2.1 (9)	2.0 (5)	1.8 (32)	**0.9 (340)***	0.6 (2)	1.6 (3)	1.0 (13)	0.9 (264)
Cardiovascular diseases	32.9 (144)	35.4 (87)	33.5 (592)	32.5 (12042)	42.2 (139)	**53.0 (97)***	47.4 (619)	45.6 (13576)
Diabetes mellitus	8.0 (35)	8.5 (21)	6.3 (111)	6.0 (2238)	9.7 (32)	10.9 (20)	10.6 (139)	8.8 (2634)
Unipolar depression	1.4 (6)	**3.7 (9)***	2.8 (49)	2.4 (883)	1.8 (6)	1.6 (3)	2.5 (33)	1.6 (489)
Alcohol-related disorders	1.1 (5)	0.8 (2)	0.7 (13)	0.6 (230)	1.5 (5)	3.3 (6)	2.9 (38)	2.3 (676)
Ischemic heart diseases	9.6 (42)	11.0 (27)	11.1 (196)	11.1 (4103)	22.5 (74)	23.5 (43)	24.2 (316)	22.7 (6773)

Cerebrovascular diseases	6.4 (28)	6.5 (16)	6.4 (113)	7.1 (2635)	10.6 (35)	10.9 (20)	11.6 (151)	11.5 (3419)
chronic obstructive pulmonary disease	4.3 (19)	6.5 (16)	3.9 (69)	3.0 (1094)	4.0 (13)	6.6 (12)	5.4 (71)	3.9 (1158)
Osteoarthritis	12.1 (53)	**6.1 (15)***	9.5 (167)	10.3 (3834)	6.1 (20)	3.8 (7)	5.7 (74)	8.9 (2643)

Comparisons made between participants and refusals, Gothenburg and Sweden.
Statistical analysis: to test differences between groups t test or χ2 test were used. *** $p < 0.001$, ** $p < 0.01$, * $p < 0.05$; bolded numbers are significant at $p < 0.05$; all other values are not significant.
[a] One individual is missing in the sociodemographic variables due personal identity number not found in the register.
Numbers in italics are not exact (true value within a 1-3 range) due to protection of the anonymity.

For peer review only

Table s6. Characteristics of 79 years old participants and refusals, and same aged individuals in Gothenburg and Sweden in 2009, stratified by sex

	Women				Men			
	Participants	Refusals	Gothenburg	Sweden	Participants	Refusals	Gothenburg	Sweden
N (%)	331 (57.1)	225 (60.8)	1566 (59.2)	33167 (57.0)	249 (42.9)	145 (39.2)	1081 (40.8)	25017 (43.0)
**Sociodemographic factors**								
Marital status, % (n)								
Married	34.1 (113)	37.8 (85)	35.2 (552)	37.7 (12519)	69.9 (174)	**54.5 (79)** **	63.5 (686)	64.8 (16217)
Widowed	40.8 (135)	34.7 (78)	38.4 (602)	43.2 (14325)	12.4 (31)	17.2 (25)	13.5 (146)	14.6 (3648)
Divorced	19.6 (65)	23.1 (52)	20.6 (322)	13.7 (4536)**	9.6 (24)	15.9 (23)	14.0 (151)	12.0 (3014)
Never married	5.4 (18)	4.4 (10)	5.7 (90)	5.4 (1787)	8.0 (20)	12.4 (18)	9.1 (98)	8.5 (2138)
Education, % (n)								
Elementary school	44.0 (*144*)	**63.0 (*142*)*** **	**50.1 (784)** *	**54.1 (17955)*** **	36.1 (90)	**52.0 (*75*)** **	39.0 (422)	**50.4 (12601)*** **
Upper secondary school	38.1 (126)	**23.6 (53)** ***	**30.6 (479)** **	**30.2 (10033)** **	39.8 (99)	31.7 (46)	37.4 (404)	**31.8 (7953)** **
Higher education	17.8 (59)	12.4 (28)	15.2 (238)	**13.3 (4419)** *	24.1 (60)	**14.5 (21)** *	20.7 (224)	**16.2 (4056)** ***
Born in Sweden, % (n)	83.7 (277)	86.2 (194)	80.4 (1259)	86.4 (28655)	85.1 (212)	86.2 (125)	80.6 (871)	**89.5 (22391)** *
Income, mean (SD)	12720 (13490)	**10440 (3880)** **	11740 (9393)	**11230 (11699)** *	16680 (10368)	14750 (11032)	16150 (15193)	**14780 (19926)** **
Paid labour, % (n)	1.0 (*2*)	1.0 (2)	0.6 (10)	0.5 (160)	2.4 (6)	2.0 (*3*)	0.8 (9 [a])*	1.7 (432)
**Hospital discharge diagnoses,** % (n)								
Cancer	16.0 (53)	11.1 (25)	14.7 (230)	12.7 (4217)	15.3 (38)	17.2 (25)	16.5 (178)	14.5 (3634)
Neuropsychiatric diseases	14.2 (47)	16.4 (37)	16.8 (263)	15.6 (5168)	19.3 (48)	13.8 (20)	20.4 (221)	17.7 (4422)
Dementia	2.1 (7)	1.3 (3)	1.8 (28)	1.7 (568)	1.6 (4)	0 (0)	2.0 (22)	1.8 (451)
Cardiovascular diseases	42.9 (142)	37.3 (84)	44.8 (702)	44.9 (14903)	53.0 (132)	53.1 (77)	56.2 (608)	56.5 (14132)
Diabetes mellitus	7.6 (25)	6.2 (14)	8.0 (126)	8.4 (2792)	10.8 (27)	9.0 (13)	11.8 (128)	2903 (11.6)
Unipolar depression	2.7 (9)	4.0 (9)	4.2 (66)	3.3 (1094)	4.4 (11)	2.8 (4)	3.7 (40)	**2.1 (526)** *
Alcohol-related disorders	1.2 (4)	0.9 (2)	0.7 (11)	0.6 (189)	0.4 (1)	**5.5 (8)** **	**3.1 (34)** *	**2.2 (554)** *
Ischemic heart diseases	12.4 (41)	9.3 (21)	14.0 (219)	14.5 (4795)	23.7 (59)	29.0 (42)	26.5 (287)	27.2 (6794)
Cerebrovascular diseases	7.6 (25)	6.7 (15)	9.6 (151)	9.8 (3236)	14.9 (37)	11.7 (17)	15.2 (164)	14.9 (3737)
chronic obstructive	3.9 (13)	4.9 (11)	5.4 (85)	4.0 (1342)	6.0 (15)	3.4 (5)	6.1 (66)	5.4 (1340)

For peer review only

pulmonary disease								
Osteoarthritis	17.2 (57)	**9.3 (21)****	14.4 (226)	14.8 (4919)	11.2 (28)	**3.4 (5)****	9.2 (99)	12.4 (3110)

Comparisons made between participants and refusals, Gothenburg and Sweden.
Statistical analysis: to test differences between groups t test or χ2 test were used. *** p <0.001, ** p <0.01, * p <0.05; bolded numbers are significant at p <0.05; all other values are not significant.
[a] Information is missing on 3 individuals
Numbers in italics are not exact (true value within a 1-3 range) due to protection of the anonymity.

For peer review only

Table s7. Characteristics of 85 years old participants and refusals, and same aged individuals in Gothenburg and Sweden in 2015, stratified by sex

	Women				Men			
	Participants	Refusals	Gothenburg	Sweden	Participants	Refusals	Gothenburg	Sweden
N (%)	251[a] (60.3)	159[a] (62.1)	1 132 (62.6)	24 417 (60.6)	165 (39.7)	97[a] (37.9)	676 (37.4)	15 899 (39.4)
**Sociodemographic factors**								
Marital status, % (n)								
Married	22.4 (56)	20.9 (33)	23.1 (262)	23.3 (5700)	61.2 (101)	49.0 (47)	55.9 (378)	57.4 (9124)
Widowed	54.0 (135)	53.8 (85)	52.1 (590)	58.6 (14319)	24.8 (41)	27.1 (26)	25.7 (174)	24.2 (3853)
Divorced	18.8 (47)	22.2 (35)	19.8 (224)	**13.1 (3196)****	7.9 (13)	11.5 (11)	11.7 (79)	11.2 (1776)
Never married	4.8 (12)	3.2 (5)	4.9 (56)	4.9 (1202)	6.1 (10)	12.5 (12)	6.7 (45)	7.2 (1146)
Education, % (n)								
Elementary school	41.0 (*103*)	**58.0 (*92*)*****	46.8 (530)	**52.7 (12865)*****	33.0 (*54*)	**50.0 (*48*)****	38.2 (258)	**48.0 (7634)*****
Upper secondary school	39.6 (99)	**28.5 (45)***	**32.0 (362)***	**30.8 (7516)****	40.6 (67)	32.3 (31)	37.7 (255)	**32.5 (5171)***
Higher education	18.0 (45)	12.0 (19)	17.0 (193)	14.3 (3483)	24.8 (41)	**14.6 (14)***	21.0 (142)	**17.9 (2840)***
Born in Sweden, % (n)	84.0 (210)	82.3 (130)	80.4 (910)	86.8 (21194)	87.3 (144)	85.4 (82)	81.8 (553)	89.6 (14251)
Income, mean (SD)	14130 (10371)	14780 (21934)	14810 (25351)	13750 (20675)	18480 (8103)	15670 (17839)	17530 (15431)	17200 (23336)
Paid labour, % (n)	N/A	N/A	N/A	N/A	N/A	N/A	N/A	N/A
**Hospital discharge diagnoses,** % (n)								
Cancer	17.9 (45)	18.9 (30)	18.8 (213)	16.3 (3990)	23.6 (39)	16.5 (16)	22.3 (151)	20.4 (3246)
Neuropsychiatric diseases	23.1 (58)	23.3 (37)	24.5 (277)	25.3 (6175)	23.6 (39)	20.6 (20)	25.0 (169)	25.6 (4070)
Dementia	<4†	<4†	2.4 (27)	3.6 (877)	<4†	<4†	1.3 (9)	3.4 (536)
Cardiovascular diseases	58.6 (147)	60.4 (96)	60.5 (685)	63.2 (15428)	67.9 (112)	69.1 (67)	68.8 (465)	70.6 (11232)
Diabetes mellitus	6.8 (17)	10.1 (16)	9.0 (102)	**11.5 (2812)***	16.4 (27)	8.2 (8)	14.5 (98)	14.9 (2364)
Unipolar depression	3.6 (9)	<4†	5.8 (66)	4.5 (1109)	6.1 (10)	<4†	4.3 (29)	**2.8 (446)***
Alcohol-related disorders	<4†	<4†	0.5 (6)	0.5 (125)	<4†	<4†	2.2 (15)	1.7 (272)
Ischemic heart diseases	12.0 (30)	11.9 (19)	16.4 (186)	**18.7 (4561)****	27.9 (46)	33.0 (32)	30.9 (209)	32.3 (5137)
Cerebrovascular diseases	10.4 (26)	16.4 (26)	13.3 (150)	14.1 (3440)	16.4 (27)	18.6 (18)	18.0 (122)	19.3 (3063)
chronic obstructive	4.8 (12)	5.0 (8)	4.8 (54)	5.2 (1272)	4.8 (8)	4.1 (4)	7.0 (47)	7.1 (1133)

For peer review only

pulmonary disease								
Osteoarthritis	23.9 (60)	17.0 (27)	21.9 (248)	21.2 (5188)	13.9 (23)	7.2 (7)	12.9 (87)	16.8 (2666)

Comparisons made between participants and refusals, Gothenburg and Sweden.
Statistical analysis: to test differences between groups t test or χ2 test were used. *** p <0.001, ** p <0.01, * p <0.05; bolded numbers are significant at p <0.05; all other values are not significant.
[a] One individual is missing in the sociodemographic variables due personal identity number not found in the register.
[b] Information is missing on 2 individuals
Numbers in italics are not exact (true value within a 1-3 range) due to protection of the anonymity.
N/A Not applicable as the cut of age for paid labour in Statistics Sweden registers is 84.
†Less than four individuals. exact number not presented to protect anonymity. Statistical comparison not performed.

For peer review only

Table s8. Characteristics of 88 years old participants and refusals, and same aged individuals in Gothenburg and Sweden in 2018

	Women				Men			
	Participants	Refusals	Gothenburg	Sweden	Participants	Refusals	Gothenburg	Sweden
N (%)	162 (62.8)	151 (61.1)	850 (64.3)	18589 (63.1)	96 (37.2)	96[a] (38.9)	471 (35.7)	10883 (36.9)
**Sociodemographic factors**								
Marital status, % (n)								
Married	10.5 (17)	15.9 (24)	**17.2 (146)***	**16.6 (3078)***	54.2 (52)	51.6 (49)	53.1 (250)	51.9 (5650)
Widowed	69.1 (112)	61.6 (93)	**59.8 (508)***	66.3 (12325)	34.4 (33)	27.4 (26)	30.4 (143)	31.0 (3375)
Divorced	16.0 (26)	19.9 (30)	18.2 (155)	12.4 (2309)	7.3 (7)	10.5 (10)	10.8 (51)	10.6 (1154)
Never married	4.3 (7)	2.6 (4)	4.8 (41)	4.7 (877)	4.2 (4)	10.5 (10)	5.7 (27)	6.5 (704)
Education, % (n)								
Elementary school	37.7 (61)	**54.3 (82)****	45.6 (388)	**51.8 (9625)*****	37.5 (36)	38.9 (37)	36.1 (170)	46.1 (5017)
Upper secondary school	36.0 (*60*)	29.1 (44)	32.1 (273)	30.9 (5741)	33.0 (*34*)	39.0 (*38*)	38.9 (183)	33.1 (3606)
Higher education	23.5 (38)	**13.2 (20)***	17.9 (152)	**15.0 (2792)****	24.0 (23)	16.8 (16)	21.2 (100)	19.2 (2087)
Born in Sweden, % (n)	82.7 (134)	82.8 (125)	80.0 (680)	87.0 (16165)	84.4 (81)	82.1 (78)	81.5 (384)	89.7 (9765)
Income, mean (SD)	19590 (30923)	16200 (19687)	16200 (22412)	15600 (23116)	18850 (13561)	19800 (20415)	19320 (16731)	18960 (29208)
Paid labour, % (n)	N/A	N/A	N/A	N/A	N/A	N/A	N/A	N/A
**Hospital discharge diagnoses,** % (n)								
Cancer	19.1 (31)	20.5 (31)	19.8 (168)	17.4 (3240)	20.8 (20)	19.8 (19)	22.9 (108)	22.0 (2394)
Neuropsychiatric diseases	24.7 (40)	**40.4 (61)****	28.1 (239)	29.0 (5384)	25.0 (24)	26.0 (25)	27.0 (127)	29.0 (3159)
Dementia	<4†	3.3 (5)	2.1 (18)	4.5 (831)	<4†	5.2 (5)	1.9 (9)	4.2 (458)
Cardiovascular diseases	66.0 (107)	67.5 (102)	66.9 (569)	70.3 (13063)	63.5 (61)	75.0 (72)	72.2 (340)	**75.5 (8214)****
Diabetes mellitus	11.7 (19)	9.3 (14)	9.5 (81)	12.5 (2318)	12.5 (12)	16.7 (16)	14.4 (68)	15.6 (1701)
Unipolar depression	5.6 (9)	6.0 (9)	6.4 (54)	4.7 (870)	6.3 (6)	4.2 (4)	4.0 (19)	2.9 (321)
Alcohol-related disorders	0 (0)	<4†	0.5 (4)	0.4 (74)	0 (0)	<4†	2.1 (10)	1.5 (168)
Ischemic heart diseases	13.6 (22)	13.2 (20)	16.6 (141)	**20.0 (3726)***	25.0 (24)	32.3 (31)	29.9 (141)	33.8 (3679)
Cerebrovascular diseases	14.2 (23)	17.2 (26)	14.8 (126)	15.3 (2850)	15.6 (15)	14.6 (14)	17.6 (83)	20.2 (2194)
chronic obstructive	5.6 (9)	<4†	4.4 (37)	5.5 (1020)	5.2 (5)	<4†	5.9 (28)	7.1 (773)

For peer review only

pulmonary disease								
Osteoarthritis	29.0 (47)	**18.5 (28)***	24.5 (208)	23.1 (4292)	15.6 (15)	10.4 (10)	14.4 (68)	18.2 (1976)

Comparisons made between participants and refusals, Gothenburg and Sweden.
Statistical analysis: to test differences between groups t test or χ2 test were used. *** p <0.001, ** p <0.01, * p <0.05; bolded numbers are significant at p <0.05; all other values are not significant.
Numbers in italics are not exact (true value within a 1-3 range) due to protection of the anonymity.
N/A Not applicable as the cut of age for paid labour in Statistics Sweden registers is 84.
†Less than four individuals. exact number not presented to protect anonymity. Statistical comparison not performed.

For peer review only

Fig s1. Response rates in different subgroups at all examination years

	2000			2005			2009			2015			2018		
	Total	Women	Men	Total	Women	Men	Total	Women	Men	Total	Women	Men	Total	Women	Men
**Response rate**	69.5% (523/752)	72.1% (281/390)	66.9% (242/362)	64.1% (765/1194)	64.0% (438/684)	64.1% (327/510)	61.1% (580/950)	59.5% (331/556)	63.2% (249/394)	62.0% (415/669)	61.3% (250/408)	63.2% (165/261)	51.2% (258/504)	51.8% (162/313)	50.3% (96/191)
**Marital status**, % *(n/N)*															
Married, *ref*	71.8% (311/433)	70.7% (130/184)	72.7% (181/249)	67.1% (418/623)	64.0% (183/286)	69.7% (235/337)	63.6% (287/451)	57.1% (113/198)	68.8% (174/253)	66.2% (157/237)	62.9% (56/89)	68.2% (101/148)	48.6% (69/142)	41.5% (17/41)	51.5% (52/101)
Widowed	69.4% (77/111)	74.5% (70/94)	**41.2%** (7/17)**	64.8% (171/264)	68.7% (145/211)	**49.1%** (26/53)**	61.7% (166/269)	63.4% (135/213)	55.4% (31/56)	61.3% (176/287)	61.4% (135/220)	61.2% (41/67)	54.9% (145/264)	54.6% (112/205)	55.9% (33/59)
Divorced	72.1% (101/140)	75.6% (65/86)	66.7% (36/54)	**58.5%* (131/224)**	58.3% (88/151)	58.9% (43/73)	**54.3%* (89/164)**	55.6% (65/117)	**51.1%* (24/47)**	56.6% (60/106)	57.3% (47/82)	54.2% (13/24)	45.2% (33/73)	46.4% (26/56)	41.2% (7/17)
Unmarried	**50.0%*** (34/68)**	61.5% (16/26)	**42.9%*** (18/42)**	**54.2%* (45/83)**	61.1% (22/36)	**48.9%** (23/47)**	57.6% (38/66)	64.3% (18/28)	**52.6%* (20/38)**	56.4% (22/39)	70.6% (12/17)	**45.5%* (10/22)**	44.0% (11/25)	63.6% (7/11)	28.6% (4/14)
**Education**, % *(n/N)*															
Elementary school ≤9y, *ref*	65.5% (253/386)	70.5% (153/217)	59.2% (100/169)	57.4% (340/592)	59.3% (213/359)	54.5% (127/233)	51.9% (234/451)	50.3% (144/286)	54.5% (90/165)	52.9% (157/297)	52.8% (103/195)	52.9% (54/102)	44.9% (97/216)	42.7% (61/143)	49.3% (36/73)
Upper secondary school	**73.8%* (177/240)**	78.8% (89/113)	69.3% (88/127)	**72.4%* (278/384)**	**73.0%*** (157/215)**	**71.6%*** (121/169)**	**69.4%*** (225/324)**	**70.4%*** (126/179)**	**68.3%* (99/145)**	**68.6%*** (166/242)**	**68.8%** (99/144)**	**68.4%* (67/98)**	53.4% (94/176)	**57.7%* (60/104)**	47.2% (34/72)
Higher education	**76.8%* (86/112)**	66.7% (34/51)	**85.2%*** (52/61)**	**71.9%* (141/196)**	67.3% (66/98)	**76.5%*** (75/98)**	**70.8%*** (119/168)**	**67.8%** (59/87)**	**74.1%** (60/81)**	**72.3%*** (86/119)**	**70.3%* (45/64)**	**74.5%** (41/55)**	**62.9%** (61/97)**	**65.5%** (38/58)**	59.0% (23/39)
**Ethnicity**, % *(n/N)*															
Born in Sweden, *ref*	71.5% (438/613)	74.1% (235/317)	68.6% (203/296)	64.8% (633/977)	65.0% (366/563)	64.5% (267/414)	60.5% (489/808)	58.8% (277/471)	62.9% (212/337)	62.5% (354/566)	61.8% (210/340)	63.7% (144/226)	51.4% (215/418)	51.7% (134/259)	50.9% (81/159)
Not born in Sweden	**61.2%* (85/139)**	63.0% (46/73)	59.1% (39/66)	60.8% (132/217)	59.5% (72/121)	62.5% (60/96)	64.1% (91/142)	63.5% (54/85)	64.9% (37/57)	59.2% (61/103)	58.8% (40/68)	60.0% (21/35)	50.0% (43/86)	51.9% (28/54)	46.9% (15/32)

The colour shading indicate response rate. The more dark colour, the higher response rate in comparison to the other subgroups the same examination year.

Comparisons made between the different subgroups at each examination year in the total groups, among women, and among men.

Statistical analysis: to test differences between groups χ2 test were used. *** p <0.001, ** p <0.01, * p <0.05; bolded numbers are significant at p <0.05; all other values are not significant.

338x190mm (300 x 300 DPI)

STROBE 2007 (v4) Statement—Checklist of items that should be included in reports of *cohort studies*

Section/Topic	Item #	Recommendation	Reported on page #
**Title and abstract**	1	(*a*) Indicate the study's design with a commonly used term in the title or the abstract	1
		(*b*) Provide in the abstract an informative and balanced summary of what was done and what was found	4
**Introduction**			
Background/rationale	2	Explain the scientific background and rationale for the investigation being reported	6
Objectives	3	State specific objectives, including any prespecified hypotheses	6
**Methods**			
Study design	4	Present key elements of study design early in the paper	6
Setting	5	Describe the setting, locations, and relevant dates, including periods of recruitment, exposure, follow-up, and data collection	6, 9
Participants	6	(*a*) Give the eligibility criteria, and the sources and methods of selection of participants. Describe methods of follow-up	6
		(*b*) For matched studies, give matching criteria and number of exposed and unexposed	N/A
Variables	7	Clearly define all outcomes, exposures, predictors, potential confounders, and effect modifiers. Give diagnostic criteria, if applicable	7-8
Data sources/ measurement	8*	For each variable of interest, give sources of data and details of methods of assessment (measurement). Describe comparability of assessment methods if there is more than one group	*7-8*
Bias	9	Describe any efforts to address potential sources of bias	8
Study size	10	Explain how the study size was arrived at	Figure 1
Quantitative variables	11	Explain how quantitative variables were handled in the analyses. If applicable, describe which groupings were chosen and why	8
Statistical methods	12	(*a*) Describe all statistical methods, including those used to control for confounding	8
		(*b*) Describe any methods used to examine subgroups and interactions	8
		(*c*) Explain how missing data were addressed	N/A
		(*d*) If applicable, explain how loss to follow-up was addressed	N/A
		(*e*) Describe any sensitivity analyses	N/A
**Results**			

For peer review only

Participants	13*	(a) Report numbers of individuals at each stage of study—eg numbers potentially eligible, examined for eligibility, confirmed eligible, included in the study, completing follow-up, and analysed	Figure 1
		(b) Give reasons for non-participation at each stage	Figure 1
		(c) Consider use of a flow diagram	Figure 1
Descriptive data	14*	(a) Give characteristics of study participants (eg demographic, clinical, social) and information on exposures and potential confounders	Table 3
		(b) Indicate number of participants with missing data for each variable of interest	N/A
		(c) Summarise follow-up time (eg, average and total amount)	N/A
Outcome data	15*	Report numbers of outcome events or summary measures over time	Table 2, Table 3
Main results	16	(*a*) Give unadjusted estimates and, if applicable, confounder-adjusted estimates and their precision (eg, 95% confidence interval). Make clear which confounders were adjusted for and why they were included	Table 2, Table 3
		(*b*) Report category boundaries when continuous variables were categorized	N/A
		(*c*) If relevant, consider translating estimates of relative risk into absolute risk for a meaningful time period	N/A
Other analyses	17	Report other analyses done—eg analyses of subgroups and interactions, and sensitivity analyses	N/A
**Discussion**			
Key results	18	Summarise key results with reference to study objectives	12
**Limitations**			
Interpretation	20	Give a cautious overall interpretation of results considering objectives, limitations, multiplicity of analyses, results from similar studies, and other relevant evidence	12
Generalisability	21	Discuss the generalisability (external validity) of the study results	15
**Other information**			
Funding	22	Give the source of funding and the role of the funders for the present study and, if applicable, for the original study on which the present article is based	16

*Give information separately for cases and controls in case-control studies and, if applicable, for exposed and unexposed groups in cohort and cross-sectional studies.

Note: An Explanation and Elaboration article discusses each checklist item and gives methodological background and published examples of transparent reporting. The STROBE checklist is best used in conjunction with this article (freely available on the Web sites of PLoS Medicine at http://www.plosmedicine.org/, Annals of Internal Medicine at http://www.annals.org/, and Epidemiology at http://www.epidem.com/). Information on the STROBE Initiative is available at www.strobe-statement.org.

For peer review only

BMJ Open

Representativeness in population-based studies of older adults – Five waves of cross-sectional examinations in the Gothenburg H70 Birth Cohort Study

Journal:	*BMJ Open*
Manuscript ID	bmjopen-2022-068165.R1
Article Type:	Original research
Date Submitted by the Author:	04-Nov-2022
Complete List of Authors:	Wetterberg, Hanna; University of Gothenburg Institute of Neuroscience and Physiology, Department of Psychiatry and Neurochemistry; University of Gothenburg, Centre for Ageing and Health (AGECAP)<br>Rydén, Lina; University of Gothenburg Institute of Neuroscience and Physiology, Department of Psychiatry and Neurochemistry,; University of Gothenburg, Centre for Ageing and Health (AGECAP)<br>Ahlner, Felicia; University of Gothenburg Institute of Neuroscience and Physiology, Department of Psychiatry and Neurochemistry; University of Gothenburg, Centre for Ageing and Health (AGECAP)<br>Falk, Hanna; Goteborgs universitet Sahlgrenska Akademin, Institute of Health and Care Sciences; Institute of Neuroscience and Physiology<br>Gudmundsson, Pia; University of Gothenburg Institute of Neuroscience and Physiology, Department of Psychiatry and Neurochemistry; University of Gothenburg, Centre for Ageing and Health (AGECAP)<br>Guo, Xinxin; University of Gothenburg Institute of Neuroscience and Physiology, Department of Psychiatry and Neurochemistry; University of Gothenburg, Centre for Ageing and Health (AGECAP)<br>Joas, Erik; University of Gothenburg Institute of Neuroscience and Physiology, Department of Psychiatry and Neurochemistry; University of Gothenburg, Centre for Ageing and Health (AGECAP)<br>Johansson, Lena; University of Gothenburg Sahlgrenska Academy, Institute of Health and Care Sciences; University of Gothenburg Sahlgrenska Academy, Department of Psychiatry and Neurochemistry<br>Kern, Silke; University of Gothenburg Institute of Neuroscience and Physiology, Department of Psychiatry and Neurochemistry; University of Gothenburg, Centre for Ageing and Health (AGECAP)<br>Fässberg, Madeleine; University of Gothenburg Institute of Neuroscience and Physiology, Psychiatry and Neurochemistry; University of Gothenburg, Department of Psychiatry and Neurochemistry<br>Najar, Jenna; University of Gothenburg Institute of Neuroscience and Physiology, Department of Psychiatry and Neurochemistry; University of Gothenburg, Centre for Ageing and Health (AGECAP)<br>Ribbe, Mats; University of Gothenburg Institute of Neuroscience and Physiology, Department of Psychiatry and Neurochemistry; University of London, Centre for Ageing and Health (AGECAP)<br>Sterner, Therese Rydberg; University of Gothenburg Institute of Neuroscience and Physiology, Department of Psychiatry and Neurochemistry; University of Gothenburg, Centre for Ageing and Health (AGECAP)

	Samuelsson, Jessica; University of Gothenburg Institute of Neuroscience and Physiology, Department of Psychiatry and Neurochemistry; University of Gothenburg, Centre for Ageing and Health (AGECAP)<br>Sacuiu, S; University of Gothenburg Institute of Neuroscience and Physiology, Department of Psychiatry and Neurochemistry; University of Gothenburg, Centre for Ageing and Health (AGECAP)<br>Sigström, Robert; University of Gothenburg Institute of Neuroscience and Physiology, Department of Psychiatry and Neurochemistry; University of Gothenburg, Centre for Ageing and Health (AGECAP)<br>Skoog, Johan; University of Gothenburg Institute of Neuroscience and Physiology, Department of Psychiatry and Neurochemistry; University of Gothenburg, Centre for Ageing and Health (AGECAP)<br>Waern, M; University of Gothenburg Institute of Neuroscience and Physiology, Department of Psychiatry and Neurochemistry; University of Gothenburg, Centre for Ageing and Health (AGECAP)<br>Zettergren, Anna; University of Gothenburg Institute of Neuroscience and Physiology, Department of Psychiatry and Neurochemistry; University of Gothenburg, Centre for Ageing and Health (AGECAP)<br>Skoog, Ingmar; University of Gothenburg Institute of Neuroscience and Physiology, Department of Psychiatry and Neurochemistry; University of Gothenburg, Centre for Ageing and Health (AGECAP)
**Primary Subject Heading**:	Epidemiology
Secondary Subject Heading:	Research methods
Keywords:	EPIDEMIOLOGY, STATISTICS & RESEARCH METHODS, Health informatics < BIOTECHNOLOGY & BIOINFORMATICS

SCHOLARONE™
Manuscripts

I, the Submitting Author has the right to grant and does grant on behalf of all authors of the Work (as defined in the below author licence), an exclusive licence and/or a non-exclusive licence for contributions from authors who are: i) UK Crown employees; ii) where BMJ has agreed a CC-BY licence shall apply, and/or iii) in accordance with the terms applicable for US Federal Government officers or employees acting as part of their official duties; on a worldwide, perpetual, irrevocable, royalty-free basis to BMJ Publishing Group Ltd ("BMJ") its licensees and where the relevant Journal is co-owned by BMJ to the co-owners of the Journal, to publish the Work in this journal and any other BMJ products and to exploit all rights, as set out in our licence.

The Submitting Author accepts and understands that any supply made under these terms is made by BMJ to the Submitting Author unless you are acting as an employee on behalf of your employer or a postgraduate student of an affiliated institution which is paying any applicable article publishing charge ("APC") for Open Access articles. Where the Submitting Author wishes to make the Work available on an Open Access basis (and intends to pay the relevant APC), the terms of reuse of such Open Access shall be governed by a Creative Commons licence – details of these licences and which Creative Commons licence will apply to this Work are set out in our licence referred to above.

Other than as permitted in any relevant BMJ Author's Self Archiving Policies, I confirm this Work has not been accepted for publication elsewhere, is not being considered for publication elsewhere and does not duplicate material already published. I confirm all authors consent to publication of this Work and authorise the granting of this licence.

For peer review only

Representativeness in population-based studies of older adults – Five waves of cross-sectional examinations in the Gothenburg H70 Birth Cohort Study

Hanna Wetterberg, MSc[ab]*, Lina Rydén, MD[abc]*, Felicia Ahlner, MSc[ab], Hanna Falk Erhag, RN, PhD[ab], Pia Gudmundsson, PhD[ab], Xinxin Guo, MD, PhD[ab],[d], Erik Joas, PhD[ab], Lena Johansson, RN, PhD[ab], Silke Kern, MD, PhD[abc], Madeleine Mellqvist Fässberg, PhD[ab], Jenna Najar, MD, PhD[abc], Mats Ribbe, MD, PhD[ab], Therese Rydberg Sterner, PhD[abe], Jessica Samuelsson, MSc[ab], Simona Sacuiu MD, PhD[abc], Robert Sigström, MD, PhD[abc], Johan Skoog, PhD[ab], Margda Waern, MD, PhD[abf], Anna Zettergren, PhD[ab], Ingmar Skoog, MD, PhD[abc]

* HW and LR are joint first authors

[a]*Neuropsychiatric Epidemiology Unit, Department of Psychiatry and Neurochemistry, Institute of Neuroscience and Physiology, Sahlgrenska Academy, at the University of Gothenburg, Sweden;* [b]*Centre for Ageing and Health (AGECAP) at the University of Gothenburg, Sweden;* [c]*Region Västra Götaland, Sahlgrenska University Hospital, Psychiatry, Cognition and Old Age Psychiatry Clinic, Gothenburg, Sweden;* [d]*Region Västra Götaland, Sahlgrenska University Hospital, Department of Mood disorders, Gothenburg, Sweden;* [e]*Aging Research Center, Department of Neurobiology, Care Sciences and Society, Karolinska Institutet and Stockholm University, Stockholm, Sweden;* [f]*Region Västra Götaland, Department of Psychiatry, Psychotic Disorders, Sahlgrenska University Hospital, Mölndal, Sweden*

E-mail addresses and ORCID for authors

Hanna Wetterberg hanna.wetterberg@gu.se (0000-0003-3836-825X)
Lina Rydén <lina.ryden@gu.se> (0000-0002-3817-2620)
Felicia Ahlner felicia.ahlner@gu.se (0000-0003-3116-7332)
Hanna Falk Erhag hanna.falk@gu.se (0000-0002-6494-2895)
Pia Gudmundsson pia.gudmundsson@gu.se (0000-0001-7173-4341)
Xinxin Guo xinxin.guo@gu.se (0000-0002-1353-287X)
Erik Joas erik.joas@gu.se (0000-0001-9546-2192)
Lena Johansson lena.maria.johansson@gu.se (0000-0002-9928-4909)
Silke Kern silke.kern@gu.se (0000-0001-7617-7214)
Madeleine Mellqvist Fässberg madeleine.mellqvist@gu.se (0000-0002-7366-4444)
Jenna Najar jenna.najar@gu.se (0000-0002-1762-4413)
Mats Ribbe mats.ribbe@gu.se (0000-0002-5998-656X)
Therese Rydberg Sterner therese.rydberg.sterner@gu.se (0000-0001-8202-8522),

For peer review only

For peer review only - http://bmjopen.bmj.com/site/about/guidelines.xhtml

Jessica Samuelsson jessica.samuelsson@gu.se (0000-0002-8891-6720)
Simona Sacuiu simona.sacuiu@neuro.gu.se (0000-0003-0472-7699)
Robert Sigström robert.sigstrom@gu.se (0000-0003-1505-1532)
Johan Skoog johan.skoog@psy.gu.se (0000-0001-9549-5875)
Margda Waern margda.waern@gu.se (0000-0002-8330-6915)
Anna Zettergren anna.zettergren@gu.se (0000-0002-7182-8417)
Ingmar Skoog ingmar.skoog@neuro.gu.se (0000-0002-9803-3438)

Corresponding author:

Hanna Wetterberg, Neuropsychiatric Epidemiology Unit, Wallinsgatan 6, SE 431 41 Mölndal

+46733424288, hanna.wetterberg@gu.se

Number of words: 3438

For peer review only

Abstract

Objectives: To describe representativeness in the Gothenburg H70 1930 birth cohort study.

Design: Repeated cross-sectional examinations of a population-based study.

Setting: Gothenburg, Sweden.

Participants: All residents in Gothenburg, Sweden, born on specific birth dates during 1930 were invited to a comprehensive health examination at ages 70, 75, 79, 85, and 88. The numbers of participants at each examination were: 524 at age 70, 767 at age 75, 580 at age 79, 416 at age 85, and 258 at age 88.

Primary outcome measures: We compared register data on sociodemographic characteristics and hospital discharge diagnoses between participants and a) refusals, b) all same-aged individuals in Gothenburg, and c) all same-aged individuals in Sweden. We also compared mortality rate between participants and refusals.

Results: Refusal rate increased with age. At two or more examination waves, participants compared to refusals, had higher educational level, more often osteoarthritis, lower mortality rates, lower prevalence of neuropsychiatric, alcohol-related and cardiovascular disorders, and were more often married. At two examination waves, participants compared to same-aged individuals in Gothenburg, had higher education and were more often born in Sweden. At two examination waves or more, participants compared to same-aged individuals in Sweden, had higher education, higher average income, less often ischemic heart disease, were less often born in Sweden, and more often divorced.

Conclusion: Participants were more similar to the target population in Gothenburg than to refusals and same-aged individuals in Sweden. Our study shows the importance to have different comparison groups when assessing representativeness of population studies, which is important in evaluating generalizability of results. The study also contributes with unique and up-to-date knowledge on participation bias in these high age groups.

Keywords: Epidemiologic studies, cohort studies, aged, selection bias

For peer review only - http://bmjopen.bmj.com/site/about/guidelines.xhtml

Article summary

Strengths and limitations of this study

- The use of multiple comparison groups provides useful information on how to quantify the representativeness of population-based studies on older adults
- Included in the comparisons are a broad range of register-based variables providing information on the differences and similarities between the groups
- The data was received on an aggregated level due to ethical regulations, hence, only univariate analyses were possible to perform

For peer review only

For peer review only - http://bmjopen.bmj.com/site/about/guidelines.xhtml

Introduction

Cross-sectional population-based studies are important to measure the societal burden of physical and mental disorders and associated factors, as well as providing baseline data for incidence studies (1, 2). Biased participation decreases representativeness and may affect results of the studies and lead to spurious findings. This occurs when those more prone to accept have specific characteristics (2). One well-known potential bias is the “healthy volunteer effect”, where participants have a lower disease burden than refusals. Other factors known to decrease representativeness are that women and individuals with higher socioeconomic status and higher education tend to participate more often (1, 3). Studies with low response rates are more vulnerable to self-selection bias than studies with high response rates.

Representativeness could be examined in different ways. One is to compare characteristics between participants and refusals. Another is to compare participants to the target population from which the sample was drawn. A third is to compare participants to the population of a larger geographical area, such as the country where the study was performed.

The Gothenburg H70 Birth Cohort Studies (the H70 studies) are multidisciplinary population studies examining birth cohorts of older populations in Gothenburg, Sweden. The aim is to study ageing and the prevalence of mental and physical disorders and their risk factors, considering the complex interactions with age, sex, gender, socio-economic gradients, secular changes, as well as psychosocial, neurobiological, and genetic factors occurring across the life course (4). The studies started in 1971 (5) and since then, five birth cohorts of 70–year olds (4), and three birth cohorts of 85-year olds (6) have been examined and followed longitudinally. The present study is concerned with the 1930 cohort, where we previously have published more than 30 papers based on cross-sectional examinations. However, no study has examined the representativeness of this cohort in detail, or made comparisons with the whole populations of Gothenburg and Sweden, or given a detailed overview of sampling methods for all examinations performed at ages 70, 75, 79, 85,

For peer review only - http://bmjopen.bmj.com/site/about/guidelines.xhtml

and 88. This type of information is important to evaluate the external validity of a study, and is essential to assess if the representativeness of studies changes over time (7). This setting gives a unique opportunity to evaluate in detail participation bias in an older population.

This paper aimed to describe the representativeness of participants of the examinations of the birth cohort born in 1930, at ages 70, 75, 79, 85, and 88 years. We studied three levels of representativeness: in relation to refusals, same-aged individuals in Gothenburg, and same-aged individuals in Sweden.

For peer review only

Methods

The H70 birth cohort 1930 was examined at five occasions. All samples included residents in Gothenburg born in 1930, who were systematically selected based on birth dates. An exception was in 2009-2011 when only those invited previously were included. Individuals who were not traceable or did not speak Swedish were excluded from the eligible samples. The invitation procedure has been nearly identical at all examination years. An invitation letter was sent to all sampled individuals, including information about the study and a consent form. After 1-2 weeks, the research staff contacted invited individuals by telephone. In cases of no contact or when the telephone number could not be found, reminders were posted. Individuals who were not able to visit the outpatient clinic were offered a home visit.

The H70 birth cohort 1930 includes participants born 1930 from the Prospective Population Study of Women (PPSW), a longitudinal study with baseline examination in 1968-1969 (8). The number of participants examined before 2000 within the PPSW study was 173 (33%) in 2000, 155 (20%) in 2005, 116 (20%) in 2009, 81 (20%) in 2015, and 53 (21%) in 2018.

Data collection procedures

For peer review only - http://bmjopen.bmj.com/site/about/guidelines.xhtml

Examinations

The examination procedures have been similar at all examinations (4), and include a six-to-eight-hour general examination. In addition, participants were invited to a range of additional examinations and a close informant interview. See Additional file, Table s1 for details regarding the specific examinations conducted at the different waves.

Comparison groups

We investigated the representativeness of the participants in the study in comparison to the refusals, same-aged individuals in Gothenburg and same-aged individuals in Sweden. Included in the comparison groups of Gothenburg and Sweden are all individuals born in 1930 who are registered residents in the municipality of Gothenburg and in Sweden, respectively. Hence, in the Gothenburg group individuals that participated in the study is included as well. This data was received from Statistics Sweden on an aggregated level.

Outcome variables

Information on sociodemographic variables for participants, refusals, and same-aged individuals from the municipality of Gothenburg as well as Sweden were received on an aggregated level from Statistics Sweden, and included marital status (married/widowed/divorced/never married), highest attained educational level (elementary/upper secondary/higher), country of birth (Sweden, yes/no), paid labour (occupational status, yes/no), and average individual income per month (means and standard deviations (SD)).

Hospital discharge diagnoses were obtained from the inpatient part of the National Patient Register (NPR), using codes from the Swedish version of the International Classification of Diseases (ICD) 9 and ICD 10. Diagnoses from 1987 up to each examination year were used in the analyses. Data was available at an individual level for participants and refusals in 2000, 2005, and 2009. Data on same-aged individuals living in Gothenburg and Sweden in 2000, 2005, 2009, 2015

For peer review only - http://bmjopen.bmj.com/site/about/guidelines.xhtml

and 2018, and data on participants and non-participants at the examinations in 2015 and 2018 were received on aggregated level from the National Board of Health and Welfare. The NPR gained full national coverage in 1987 and all citizens in Sweden have access to medical care and therefore equal chance to be included in the NPR (9). The hospital discharge diagnoses included cancer, neuropsychiatric disorders (including dementia disorders), cardiovascular diseases, diabetes mellitus, unipolar depression, alcohol-related disorders, ischemic heart disease, cerebrovascular diseases, chronic obstructive pulmonary disease, and osteoarthritis (see Additional file, Table s2, for ICD codes).

Dates of death were available from the Swedish Tax Agency.

Statistical analyses

Differences in sociodemographic factors and hospital discharge diagnoses between participants and refusals, same-aged individuals in Gothenburg and same-aged individuals in Sweden were compared using Pearson's chi-squared test or Fisher's exact test for categorical variables and independent samples t-test for continuous variables. As the data was received on an aggregated level, only univariate analyses were performed.

Cox proportional hazards models adjusted for sex were used to compare mortality between participants and refusals, presented as hazard ratios (HR) and 95% confidence intervals (CI). The risk time was calculated as the surviving time up to five years from age 70, 75, 79, 85, and two years from age 88. Individuals who emigrated during follow-up were excluded from mortality analyses, as their status is not known (four from examination in 2000, two from 2005, none from 2009, and one each from 2015 and 2018). The proportional hazard assumption was verified using Schoenfeld residuals. Mortality analyses were performed in R (version 3.5.3) using survminer and survival packages. $P<0.05$ (two-tailed) was considered statistically significant in all analyses.

For peer review only

This report complied with the Strengthening the Reporting of Observational Studies in Epidemiology guidelines for observational studies (including cross-sectional studies), as stated in the Research Checklist (10).

Figure 1 to be placed here

Participant and public involvement (PPI) statement

None.

Results

Samples (Figure 1)

The sample flow charts are displayed in Figure 1.

In 2000, 775 70-year-old residents in Gothenburg were sampled. Among these, 753 were eligible (see Figure 1), and 524 accepted to participate (response rate: 69.6%). Of these, 14 (2.7%) were examined in their home. The study was conducted September 4, 2000 - March 28, 2002.

In 2005, 1250 75-year-old residents in Gothenburg were sampled. Among these, 1196 were eligible (see Figure 1), and 767 accepted to participate (response rate: 64.1%). Of these, 117 (15.3%) were examined in their home. The study was conducted September 6, 2005 - March 20, 2007.

In 2009, 1056 79-year-old residents in Gothenburg, who had been invited at the 2000 or 2005 examinations were invited, and 950 were eligible (see Figure 1), and 580 accepted to participate (response rate: 61.1%). Of these, 144 (24.8%) were examined in their home, and 6 (1.0%) had a combination of clinic and at home examinations. The study was conducted November 6, 2009 - September 19, 2011.

For peer review only - http://bmjopen.bmj.com/site/about/guidelines.xhtml

In 2015, 764 85-year-old residents in Gothenburg were sampled. Among these, 672 were eligible (see Figure 1), and 416 accepted to participate (eligible response rate: 61.9%). Of these, 174 (41.8%) were examined in their home, and 18 (4.3%) had a combination of clinic and home examinations. The study was conducted October 7, 2015 - May 29, 2017.

In 2018, 555 88-year-old residents in Gothenburg were sampled. Among these, 505 were eligible (see Figure 1), and 258 accepted to participate (eligible response rate: 51.1%). Of these, 131 (50.8%) were examined in their home, and 11 (4.3%) had a combination of clinic and home examinations. The study was conducted September 24, 2018 - December 20, 2019.

Results

The mean age of those examined was 70.6 years (SD 0.3) in 2000, 75.7 years (SD 0.4) in 2005, 80.1 years (SD 0.2) in 2009, 86.0 years (SD 0.2) in 2015, and 88.6 years (SD 0.2) in 2018. Among those who participated, 74.1% (n=811) participated more than once.

The response rate was higher in 2000 and lower in 2018 compared to all other examinations (Table 1). There were no significant differences in response rates between women and men, although women had slightly higher response rates in 2000 (72.1% versus 66.9%, p=0.122) and lower in 2009 (59.5% versus 63.2%, p=0.254) (see Additional file 2, Figure s1). For response rates in various subgroups, see Additional file 2, Figure s1. The proportion excluded from the sample because they did not speak Swedish, died after sampling, or were non-traceable was higher in 2009, 2015, and 2018 compared to 2000 and 2005 (Additional file 1, Table s3).

Table 1. Comparison of response rates between examinations

Exam. Year<br>Response rate	2000<br>69.5%	2005<br>64.1%	2009<br>61.1%	2015<br>62.0%	2018<br>51.2%
2000<br>69.5%	-	p = 0.013	p < 0.000	p = 0.003	p < 0.000
2005<br>64.1%		-	p = 0.151	p = 0.381	p < 0.000

For peer review only - http://bmjopen.bmj.com/site/about/guidelines.xhtml

2009 61.1%			-	$p = 0.690$	$p < 0.000$
2015 62.0%				-	$p < 0.000$
2018 51.2%					-
Statistical analysis: to test differences between groups χ2 test were used.					

Mortality

Mortality was higher in refusals compared to participants at all examination years (Figure 2). The test of Schoenfeld's residuals indicated deviation from the proportional hazards assumption in the survival analysis in 2005, showing that the higher mortality among refusals was limited to the first two years (Figure 2).

Figure 2 to be placed here

Sociodemographic factors and hospital discharge diagnoses from registers

Table 2 shows sociodemographic factors and hospital discharge diagnoses for participants, refusals, and all same-aged individuals living in Gothenburg and in Sweden during the corresponding year. Analyses stratified by sex are shown in Additional file 1, Table s4-s8.

For peer review only

For peer review only - http://bmjopen.bmj.com/site/about/guidelines.xhtml

Table 2. Characteristics of participants and refusals, and same aged individuals in Gothenburg and Sweden by age and examination year.

	70-year-olds in 2000				75-year-olds in 2005				79-year-olds in 2009			
	Participants	Refusals	Gothenburg	Sweden	Participants	Refusals	Gothenburg	Sweden	Participants	Refusals	Gothenburg	Sweden
N	524[a]	229	3508	75 468	767[a]	429	3075	66 855	580	370	2647	58184
Sociodemographic factors												
Marital status, % (n)												
Married	59.5 (311)	53.3 (122)	56.1 (1968)	61.9 (46 697)	54.6 (418)	**47.8 (205)***	52.4 (1 611)	55.7 (37 238)	49.5 (287)	44.3 (164)	46.8 (1 238)	49.4 (28 736)
Widowed	14.7 (77)	14.8 (34)	15.8 (553)	16.3 (12 291)	22.4 (171)	21.7 (93)	21.5 (661)	23.5 (15 715)	28.6 (166)	27.8 (103)	28.3 (748)	30.9 (17 973)
Divorced	19.3 (101)	17.0 (39)	19.4 (679)	**14.0 (10 576)*****	17.1 (131)	21.7 (93)	18.2 (560)	**13.5 (9 051)****	15.3 (89)	20.3 (75)	17.9 (473)	13.0 (7 550)
Never married	6.5 (34)	**14.8 (34)*****	8.8 (308)	7.8 (5 904)	5.9 (45)	8.9 (38)	7.9 (243)	7.3 (4 851)	6.6 (38)	7.6 (28)	7.1 (188)	6.7 (3 925)
Education, % (n)												
Elementary school	48.3 (*253*)	**58.1 (133)***	47.8 (1678)	**53.9 (40 694)***	44.4 (*340*)	**58.7 (252)*****	47.7 (1 467)	**53.2 (35 584)*****	40.3 (*234*)	**58.6 (217)*****	**45.6 (1 206)***	**52.5 (30 556)*****
Upper secondary school	33.8 (177)	27.5 (63)	32.8 (1149)	30.5 (23 027)	36.3 (278)	**24.7 (106)*****	32.7 (1 005)	**30.7 (20 553)*****	38.8 (225)	**26.8 (99)*****	**33.4 (883)***	**30.9 (17 986)*****
Higher education	16.4 (86)	11.4 (26)	15.5 (545)	13.6 (10 291)	18.4 (141)	**12.8 (55)***	16.0 (493)	**14.1 (9 408)*****	20.5 (119)	**13.2 (49)****	17.5 (462)	**14.6 (8 475)*****
Born in Sweden, % (n)	83.8 (438)	**76.4 (175)***	80.4 (2821)	87.6 (66 124)**	82.7 (633)	80.2 (344)	80.5 (2 474)	**87.7 (58 640)*****	84.3 (489)	86.2 (319)	80.5 (2 130)*	**87.7 (51 046)***
Income, mean (SD)	11 469 (8 677)	10 214 (7 958)	**10 253 (9 401)****	**10 481 (30 318)***	11 710 (8 118)	11 131 (7 780)	11 549 (8 803)	11 325 (17 293)	14 420 (12 394)	**12 129 (7 815)*****	13 541 (12 292)	**12 756 (15 869)****
Paid labour, % (n)	5.2 (27)	4.4 (*10*)	3.9 (138)	3.8 (2 850)	2.5 (19)	1.6 (7)	2.4 (75)	2.1 (1 425)	1.4 (*8*)	1.0 (5)	0.7 (19[b])	1.0 (592)
Hospital discharge diagnoses, % (n)												
Cancer	7.4 (39)	9.6 (22)	9.0 (314)	7.3 (5488)	13.2 (101)	12.4 (53)	12.9 (397)	**10.6 (7088)***	15.7 (91)	13.5 (50)	15.4 (408)	13.5 (7 851)
Neuropsychiatric diseases	6.1 (32)	**14.4 (33)*****	**10.1 (2353)****	8.4 (6357)	11.6 (89)	**16.1 (69)***	14.1 (432)	12.1 (8070)	16.4 (95)	15.4 (57)	18.3 (484)	16.5 (9 590)
Dementia	0.8 (4)	**3.1 (7)***	0.6 (20)	0.5 (357)	1.4 (11)	1.9 (8)	1.5 (45)	0.9 (604)	1.9 (11)	0.8 (3)	1.9 (50)	1.8 (1 019)

For peer review only - http://bmjopen.bmj.com/site/about/guidelines.xhtml

Cardiovascular diseases	23.7 (124)	**34.9 (80)****	26.4 (927)	26.7 (20 109)	36.9 (283)	**42.9 (184)***	39.4 (1 211)	38.3 (25 618)	47.2 (274)	43.5 (161)	49.5 (1 310)	49.9 (29 035)
Diabetes mellitus	4.4 (23)	4.8 (11)	4.9 (171)	4.9 (3 685)	8.7 (67)	9.6 (41)	8.1 (250)	7.3 (4 872)	9.0 (52)	7.3 (27)	9.6 (254)	9.8 (5 695)
Unipolar depression	0.8 (4)	0.0 (0)	1.5 (54)	1.4 (1 059)	1.6 (12)	**2.8 (12)****	2.7 (82)	2.1 (1 372)	3.4 (20)	3.5 (13)	4.0 (106)	2.8 (1 620)
Alcohol-related disorders	1.5 (8)	**5.2 (12)****	1.9 (65)	1.4 (1 056)	1.3 (10)	1.9 (8)	1.7 (51)	1.4 (906)	0.9 (5)	**2.7 (10)***	1.7 (45)	1.3 (743)
Ischemic heart diseases	10.3 (54)	12.7 (29)	11.5 (403)	11.5 (8 641)	15.1 (116)	16.3 (70)	16.7 (512)	16.3 (1 087)	17.2 (100)	17.0 (63)	19.1 (506)	19.9 (11 589)
Cerebrovascular diseases	2.7 (14)	**8.3 (19)*****	**5.4 (191)****	**5.9 (4 422)****	8.2 (63)	8.4 (36)	8.6 (264)	9.1 (6 054)	10.7 (62)	8.6 (32)	11.9 (315)	12.0 (6 973)
chronic obstructive pulmonary disease	2.3 (12)	**7.4 (17)*****	3.3 (115)	2.2 (1 619)	4.2 (32)	6.5 (28)	4.6 (140)	3.4 (2 252)	4.8 (28)	4.3 (16)	5.7 (151)	4.6 (2 682)
Osteoarthritis	6.3 (33)	**2.2 (5)***	4.7 (165)	5.8 (4 384)	9.5 (73)	**5.1 (22)****	7.8 (241)	9.7 (6 477)	14.7 (85)	**7.0 (26)*****	12.3 (325)	13.8 (8 029)

Comparisons made between participants and refusals, Gothenburg and Sweden.

Statistical analysis: to test differences between groups t test or χ2 test were used. *** *p* <0.001, ** *p* <0.01, * *p* <0.05; bolded numbers are significant at *p* <0.05; all other values are not significant.

[a] One individual is missing in the sociodemographic variables due personal identity number not found in the register.

[b] Information is missing on 3 individuals (all men)

Numbers in italics are not exact (true value within a 1-3 range) due to protection of the anonymity.

For peer review only - http://bmjopen.bmj.com/site/about/guidelines.xhtml

Table 2 continued. Characteristics of participants and refusals, and same aged individuals in Gothenburg and Sweden by age and examination year.

	85-year-olds in 2015				88-year-olds in 2018			
	Participants	Refusals	Gothenburg	Sweden	Participants	Refusals	Gothenburg	Sweden
N	416[a]	256[b]	1 808	40 316	258	247[a]	1 321	29472
Sociodemographic factors								
Marital status, % (n)								
Married	37.8 (157)	31.5 (80)	35.4 (640)	36.8 (14 824)	26.7 (69)	29.7 (73)	30.0 (396)	29.6 (8 728)
Widowed	42.4 (176)	43.7 (111)	42.3 (764)	45.1 (18 172)	56.2 (145)	48.4 (119)	**49.3 (651)***	53.3 (15 700)
Divorced	14.5 (60)	18.1 (46)	16.8 (303)	12.3 (4 972)	12.8 (33)	16.3 (40)	15.6 (206)	11.8 (3 463)
Never married	5.3 (22)	6.7 (17)	5.6 (101)	5.8 (2 348)	4.3 (11)	5.7 (14)	5.1 (68)	5.4 (1 581)
Education, % (n)								
Elementary school	37.8 (157)	**55.1 (140)*****	**43.6 (788)***	**50.8 (20 499)*****	37.6 (97)	**48.4 (119)***	42.2 (558)	**49.7 (14 642)*****
Upper secondary school	40.0 (166)	**29.9 (76)****	**34.1 (617)***	**31.5 (12 687)*****	36.4 (94)	33.3 (82)	34.5 (456)	31.7 (9 347)
Higher education	20.7 (86)	**13.0 (33)***	18.5 (335)	**15.7 (6 323)****	23.6 (61)	**14.6 (36)***	19.1 (252)	**16.6 (4 879)****
Born in Sweden, % (n)	85.3 (354)	83.5 (212)	**80.9 (1 463)***	87.9 (35 445)	83.3 (215)	82.5 (203)	80.5 (1 064)	**88.0 (25 930)***
Income, mean (SD)	15 860 (9 759)	15 116 (20 451)	15 827 (22 202)	15 111 (21 828)	19 315 (25 829)	17 590 (20 007)	17 312 (20 615)	16 841 (25 586)
Paid labour, % (n)	N/A	N/A	N/A	N/A	N/A	N/A	N/A	N/A
Hospital discharge diagnoses, % (n)								
Cancer	20.2 (84)	18.0 (46)	20.1 (364)	17.9 (7 236)	19.8 (51)	20.2 (50)	20.9 (276)	19.1 (5 634)
Neuropsychiatric diseases	23.3 (97)	22.3 (57)	24.7 (446)	25.4 (1 0245)	24.8 (64)	**34.8 (86)***	27.7 (366)	29.0 (8 543)
Dementia	3.1 (13)	1.6 (4)	2.0 (36)	3.5 (1 413)	<4†	4.0 (10)	2.0 (27)	4.4 (1289)
Cardiovascular diseases	62.3 (259)	63.7 (163)	63.6 (1 150)	66.1 (26 660)	65.1 (168)	70.4 (174)	68.8 (909)	**72.2 (21 277)***
Diabetes mellitus	10.6 (44)	9.4 (24)	11.1 (200)	12.8 (5 176)	12.0 (31)	12.1 (30)	11.3 (149)	13.6 (4 019)

For peer review only

Unipolar depression	4.6 (19)	4.7 (12)	5.3 (95)	3.9 (1 555)	5.8 (15)	5.3 (13)	5.5 (73)	4.0 (1 191)
Alcohol-related disorders	<4†	<4†	1.2 (21)	1.0 (397)	0.0 (0)	<4†	1.1 (14)	0.8 (242)
Ischemic heart diseases	18.3 (76)	9.9 (51)	21.8 (395)	**24.1 (9 698)****	17.8 (46)	20.6 (51)	21.3 (282)	**25.1 (7 405)****
Cerebrovascular diseases	12.7 (53)	17.2 (44)	15.0 (272)	16.1 (6 503)	14.7 (38)	16.2 (40)	15.8 (209)	17.1 (5 044)
chronic obstructive pulmonary disease	4.8 (20)	4.7 (12)	5.6 (101)	6.0 (2 405)	5.4 (14)	2.8 (7)	4.9 (65)	6.1 (1 793)
Osteoarthritis	20.0 (83)	**13.3 (34)***	18.5 (335)	19.5 (7 854)	24.0 (62)	**15.4 (38)***	20.9 (276)	21.3 (6 268)

Comparisons made between participants and refusals, Gothenburg and Sweden.
Statistical analysis: to test differences between groups t test or χ2 test were used. *** *p* <0.001, ** *p* <0.01, * *p* <0.05; bolded numbers are significant at *p* <0.05; all other values are not significant.
[a] One individual is missing in the sociodemographic variables due personal identity number not found in the register.
[b] Two individuals are missing in the sociodemographic variables due personal identity number not found in the register.
N/A Not applicable as the cut of age for paid labour in Statistics Sweden registers is 84.
Numbers in italics are not exact (true value within a 1-3 range) due to protection of the anonymity.
†Less than four individuals. Exact number not presented to protect anonymity. Statistical comparison not performed.

For peer review only

Participants compared to refusals

Compared to refusals, participants had higher educational level and more often osteoarthritis at all examinations, less often neuropsychiatric disorders in 2000, 2005, and 2018, less often cardiovascular disorders in 2000 and 2005, were more often married in 2000 and 2005, had higher average income per month in 2009, had less often alcohol-related disorders in 2000 and 2009, were more often born in Sweden, had less often cerebrovascular diseases, chronic obstructive pulmonary disease, and dementia in 2000, and less often unipolar depression in 2005 (Table 2).

Participants compared to the target population of same-aged individuals in Gothenburg

The participants comprised 14.9% (2000), 24.9% (2005), 21.9% (2009), 23.0% (2015), and 19.5% (2018) of the target population, i.e. all same-aged individuals in the municipality of Gothenburg.

Compared to same-aged individuals in Gothenburg, participants had higher educational level in 2009 and 2015, had higher average income per month in 2000, were more often born in Sweden in 2009 and 2015, were more often widowed in 2018, and had less often cerebrovascular and neuropsychiatric disorders in 2000 (Table 2).

Participants compared to same-aged individuals in Sweden

Compared to same-aged individuals in Sweden, participants had higher educational level at all examinations, were less often born in Sweden at all examinations except in 2015, had higher average income per month in 2000 and 2009, were more often divorced in 2000 and 2005, had less often ischemic heart disease in 2015 and 2018, more often cancer in 2005, less often cardiovascular diseases in 2018, and less often cerebrovascular diseases in 2000 (Table 2).

Discussion

We examined a birth cohort from Gothenburg, Sweden, at repeated cross-sectional examinations from age 70 to 88, and found that response rate declined with age. Participants were examined regarding three levels of representativeness: in relation to refusals, in relation to same-aged

For peer review only - http://bmjopen.bmj.com/site/about/guidelines.xhtml

individuals in Gothenburg (the target population), and in relation to same-aged individuals in Sweden. We found a number of differences in sociodemographic factors and diseases in registry data between participants and refusals, as well as a lower mortality rate among participants. While similar proportions were observed in participants and the target population in Gothenburg for several factors, proportions differed when comparing to the same-aged population for the entire country of Sweden.

This study has both strengths and limitations. The strengths of this study include five time-points of cross-sectional health examinations, the use of register data to compare participants not only with refusals, but also with same-aged individuals from Gothenburg and same-aged individuals from the entire country. There are also several limitations. First, the same individuals are included at repeated examinations and might thus be influenced by the Hawthorne effect, i.e., they might change behaviours as a result of their participation, resulting in increased differences between participants and refusals (11). Second, participants make up 15-25% of the total comparison group of individuals in Gothenburg. This made the comparison group more similar to participants. On the other hand, if we excluded participants, the comparison group would have been less representative of same-aged individuals in Gothenburg. Third, using register data to compare participants with the target population might miss important differences. A previous study found only minor differences in disease burden between participants and non-participants when using registers, but large differences when using clinical and biochemical variables from a baseline examination conducted 20 years earlier (12). It should also be noted that the sensitivity of disorders in the NPR varies, with higher sensitivity for more severe disorders and less for milder disorders (9, 13). Fourth, in some years the examinations were delayed due to logistic issues, creating a higher mean age in the population examined than was targeted. This increased the number of individuals who died or developed disorders before the examination, a bias which becomes more important with age. Finally, a small group was excluded due to either not speaking Swedish (1.6% to 4.1%) or not being traceable (0.5%

For peer review only - http://bmjopen.bmj.com/site/about/guidelines.xhtml

to 3.6%). These groups were however small, and the exclusion did probably not affect the main results.

The participation rate was initially 70% at age 70, declined to 61-64% at ages 75-85, and dropped to 51% at age 88. Response rates between 50-70% has previously been reported in similar Swedish population-based studies of adults aged 65 years and older (14), with indications that non-response was higher among the oldest population. Possible reasons for declining response rate with advancing age may be participation exhaustion, an age effect, or a period effect. Participation exhaustion might occur due to a high number of extensive examinations. To minimize this effect, we offered to perform the examination at the participant's homes, or to split up the examinations at different occasions (15). However, the response rates in our previous studies rather support an age effect where the response rate among 70-year-olds born 1944 and 1930 was around 70% (4), and among 85-year-olds born 1923-24 and 1930 around 60% (6). Other explanations for an age effect, reported also by others (16-18), include cognitive impairment (17, 18), frailty, and illness (17), which all increase with age. Population-based studies in older populations might thus be less representative with increasing age. Support for a period effect is suggested by the declining response rates in research studies over the last decades (1). For example, the first H70 examination conducted in 1971 on 70-years-olds had a response rate of 85% (5). At that examination, participants and non-participants were very similar, in contrast to the examination of 70-year-olds in our study. Another example is a Finnish health survey (19) conducted 1987-2012. They reported a declining participation rate over time in all socioeconomic groups, but a steeper decline among those with low educational level. This resulted in increased differences between participants and non-participants (19). Reasons for period effects include an overall decrease in social participation, increased complexity of modern life, lower trust in society, science, and research, increased burden due to increasing number of invitations to participate in studies, and increasingly demanding research

For peer review only

For peer review only - http://bmjopen.bmj.com/site/about/guidelines.xhtml

projects (7). This increases the risk of healthy-responder bias, making the results from population-based studies less generalizable and cohort comparisons more difficult to perform.

Our finding that participants in general were healthier and had higher education and income than refusals are in line with a number of population-based studies. For example, participants compared to refusals are reported to be younger (16-18), more often cohabiting or married (16, 20-22) and born in the study country or in neighbouring countries (20, 21), have higher income (21), educational level (20, 22), socioeconomic status (12), better health status (12, 23), less often a diagnosis in hospital discharge registers (12, 16, 20), and lower mortality rates (12, 16). Among older persons, participants reportedly have less cognitive impairment (17, 18), illness, and frailty (17). Interestingly, refusals had less often osteoarthritis in the NPR compared to participants at all examinations, in contrast to what was observed for most other disorders. Osteoarthritis is a disorder with a fairly large proportion of undiagnosed cases (24). It is thus possible that those not seeking medical attention or receiving specialized care for osteoarthritis are also less prone to participate in health examination studies.

We also examined differences between participants and the total population of same-aged individuals in Gothenburg, i.e. the target population, to get a better appreciation of external validity than is achieved by only examining differences compared to refusals. Our finding that participants were more similar to its target population than to refusals, especially at younger ages, is in line with previous studies (12, 22). However, there were some differences. Participants had higher educational level and were more often born in Sweden at ages 79 and 85, had less often cerebrovascular and neuropsychiatric disorders and higher mean income at age 70, and were more often widowed at age 88. The proportion being excluded due to not speaking Swedish was larger at ages 79 and 85, compared to the other examination years. This might explain why participants compared to same-aged individuals in Gothenburg were more often born in Sweden at these ages.

For peer review only - http://bmjopen.bmj.com/site/about/guidelines.xhtml

There were more differences between participants and same-aged individuals in Sweden. This is not surprising, as Gothenburg is one of Sweden's largest cities, and a university city. In addition, it has a higher proportion of individuals born outside Sweden, and higher educational level than rural areas of Sweden. This illustrates the difficulty to generalize findings from a local target population to even a relatively small and homogenous population, as Sweden.

We found no significant differences in response rates between women and men, in contrast to other population-based studies, which report higher response rates among women (16, 20, 22, 25). However, in two of these studies there were no sex differences at higher ages (16, 25), and the other two only included individuals aged below 64 and 54 years, respectively (20, 22). Some studies report that sex differences in response rates diminish with age (22, 26).

Our study thus shows that although there are differences between participants and refusals, participants are more similar to the target population overall. To further minimize potential selection bias in future studies on older adults, adjusting the examinations to meet the needs of those more prone to decline might be an alternative. In the present study, home visits were offered to those who did not want to visit the clinic (15). However, further adjustments, such as shorter examinations, telephone interviews or questionnaires to those who are hesitant of participation might be applied. This would, however, potentially induce measurement errors. There are also statistical methods to potentially account for missing data, such as multiple imputation or weighting methods that could counteract some of the issues of selection bias (27).

Conclusion

In conclusion, our data show that participants were similar to the target population of Gothenburg. However, participants differed most compared to refusals, and to a certain extent to same-aged individuals in Sweden, indicating that the studies might underestimate the burden of disease. These results contribute with unique and up-to-date knowledge on participation bias in these high age

For peer review only - http://bmjopen.bmj.com/site/about/guidelines.xhtml

groups, and shows the importance to have different comparison groups when assessing representativeness. This finding is readily transferable to similar studies, and since population-based studies on older adults contribute with information on disease distribution and knowledge of risk-disease associations, it is important to be aware of potential limitations in the representativeness that might hamper generalizability.

For peer review only

For peer review only - http://bmjopen.bmj.com/site/about/guidelines.xhtml

Acknowledgments

Funding

The study was financed by grants from the Swedish state under the agreement between the Swedish government and the county councils, the ALF-agreement (ALF 716681); Stena Foundation; Swedish Research Council (11267, 2005-8460, 2007-7462, 2012-5041, 2015-02830, 2019-01096, 2013-8717, NEAR 2017-00639); Swedish Research Council for Health, Working Life and Wellfare (2004-0145, 2006-0596, 2008-1111, 2010-0870, 2013-1202, 2018-00471, 2001-2646, 2003-0234, 2004-0150, 2006-0020, 2008-1229, 2012-1138, AGECAP 2013-2300, 2013-2496); Konung Gustaf V:s och Drottning Victorias Frimurarestiftelse; Hjärnfonden (FO2014-0207, FO2016-0214, FO2018-0214, FO2019-0163, FO2020-0235); Alzheimerfonden (AF-554461, AF-647651, AF-743701, AF-844671, AF-930868, AF-940139); Eivind och Elsa K:son Sylvans stiftelse; The Alzheimer's Association Zenith Award (ZEN-01-3151); The Alzheimer's Association Stephanie B. Overstreet Scholars (IIRG-00-2159); The Bank of Sweden Tercentenary Foundation; Stiftelsen Söderström-Königska Sjukhemmet; Stiftelsen för Gamla Tjänarinnor; Handlanden Hjalmar Svenssons Forskningsfond.

Author SK was financed by grants from the Swedish state under the agreement between the Swedish government and the county councils, the ALF-agreement (ALFGBG-81392, ALF GBG-771071); the Alzheimerfonden (AF-842471, AF-737641); The Swedish Research Council (2019-02075); Stiftelsen Demensfonden; Stiftelsen Hjalmar Svenssons Forskningsfond; and Stiftelsen Wilhelm och Martina Lundgrens vetenskapsfond. Author SS was financed by grants from the ALF-agreement (ALFGBG-637271); and the Sahlgrenska Academy Homecoming Fellowship (Dnr V2012/294).

Disclosure of interest

The authors have no relevant financial or non-financial interests to disclose.

Author Contributions

For peer review only

Hanna Wetterberg, Lina Rydén, and Ingmar Skoog had full access to all the data in the study and take responsibility for the integrity of the data and the accuracy of the data analysis. Concept and design: Wetterberg, Rydén, and Skoog. Acquisition, analysis, or interpretation of data: Hanna Wetterberg, Lina Rydén, Felicia Ahlner, Hanna Falk Erhag, Pia Gudmundsson, Xinxin Guo, Erik Joas, Lena Johansson, Silke Kern, Madeleine Mellqvist Fässberg, Jenna Najar, Mats Ribbe, Therese Rydberg Sterner, Jessica Samuelsson, Simona Sacuiu, Robert Sigström, Johan Skoog, Margda Waern, Anna Zettergren, and Ingmar Skoog. Drafting of the manuscript: Wetterberg, Rydén, and Skoog. Statistical analysis: Wetterberg and Rydén. Principal investigator: Ingmar Skoog. Critical revision of the manuscript for important intellectual content: Hanna Wetterberg, Lina Rydén, Felicia Ahlner, Hanna Falk Erhag, Pia Gudmundsson, Xinxin Guo, Erik Joas, Lena Johansson, Silke Kern, Madeleine Mellqvist Fässberg, Jenna Najar, Mats Ribbe, Therese Rydberg Sterner, Jessica Samuelsson, Simona Sacuiu, Robert Sigström, Johan Skoog, Margda Waern, Anna Zettergren, and Ingmar Skoog.

Acknowledgement statement

The authors would like to thank all participants of the H70 studies in Gothenburg, and the research group members for their cooperation in data collection and management. We also acknowledge seminal contributions to this study by the late Dr. Svante Östling.

Ethics approval

The H70 study was conducted according to the ethical standards of the institutional committee and with the 1964 Helsinki Declaration and its later amendments or comparable ethical standards. The Regional Ethical Review Board approved the H70 studies in Gothenburg (approval numbers: 240800/S227-00, 041104/T453-04, 090309/075-09, 270415/131-15, and 230418/278-18). The Swedish Ethical Review Authority has approved the publication of aggregated data on the

For peer review only

For peer review only - http://bmjopen.bmj.com/site/about/guidelines.xhtml

individuals that declined participation in the study (approval numbers: 170620/2020-02181, 170620/2020-02188).

Consent to participate

Informed consent was obtained from all participants included in the study. In cases of dementia, informed consent was obtained from a close relative.

Availability of data and material

All data and analyses generated during the current study are available from the corresponding author on reasonable request.

References

1. Galea S, Tracy M. Participation Rates in Epidemiologic Studies. Ann Epidemiol. 2007;17(9):643-53.
2. Levin KA. Study design III: Cross-sectional studies. Evidence-Based Dentistry. 2006;7:24-5.
3. Mindell JS, Giampaoli S, Goesswald A, Kamtsiuris P, Mann C, Männistö S, Morgan K, Shelton NJ, Verschuren WM, Tolonen H. Sample selection, recruitment and participation rates in health examination surveys in Europe--experience from seven national surveys. BMC Med Res Methodol. 2015;15:78.
4. Rydberg Sterner T, Ahlner F, Blennow K, Dahlin-Ivanoff S, Falk H, Havstam Johansson L, Hoff M, Holm M, Horder H, Jacobsson T, Johansson B, Johansson L, Kern J, Kern S, Machado A, Mellqvist Fassberg M, Nilsson J, Ribbe M, Rothenberg E, Ryden L, Sadeghi A, Sacuiu S, Samuelsson J, Sigstrom R, Skoog J, Thorvaldsson V, Waern M, Westman E, Wetterberg H, Zetterberg H, Zetterberg M, Zettergren A, Ostling S, Skoog I. The Gothenburg H70 Birth cohort study 2014-16: design, methods and study population. Eur J Epidemiol. 2019;34(2):191-209.
5. Rinder L, Roupe S, Steen B, Svanborg A. Seventy-year-old people in Gothenburg. A population study in an industrialized Swedish city. Acta Med Scand. 1975;198(5):397-407.
6. Skoog I, Borjesson-Hanson A, Kern S, Johansson L, Falk H, Sigstrom R, Ostling S. Decreasing prevalence of dementia in 85-year olds examined 22 years apart: the influence of education and stroke. Sci Rep. 2017;7.
7. Morton SM, Bandara DK, Robinson EM, Carr PE. In the 21st Century, what is an acceptable response rate? Aust N Z J Public Health. 2012;36(2):106-8.
8. Bengtsson C, Blohme G, Hallberg L, Hallstrom T, Isaksson B, Korsan-Bengtsen K, Rybo G, Tibblin E, Tibblin G, Westerberg H. The study of women in Gothenburg 1968-1969--a population study. General design, purpose and sampling results. Acta Med Scand. 1973;193(4):311-8.
9. Ludvigsson JF, Andersson E, Ekbom A, Feychting M, Kim J-L, Reuterwall C, Heurgren M, Olausson PO. External review and validation of the Swedish national inpatient register. BMC Public Health. 2011;11:450-.
10. von Elm E, Altman DG, Egger M, Pocock SJ, Gotzsche PC, Vandenbroucke JP, Initiative S. Strengthening the Reporting of Observational Studies in Epidemiology (STROBE) statement: guidelines for reporting observational studies. BMJ. 2007;335(7624):806-8.
11. McCambridge J, Witton J, Elbourne DR. Systematic review of the Hawthorne effect: New concepts are needed to study research participation effects. J Clin Epidemiol. 2014;67(3):267-77.

For peer review only - http://bmjopen.bmj.com/site/about/guidelines.xhtml

12. Drivsholm T, Eplov LF, Davidsen M, Jørgensen T, Ibsen H, Hollnagel H, Borch-Johnsen K. Representativeness in population-based studies: A detailed description of non-response in a Danish cohort study. Scandinavian Journal of Public Health. 2006;34(6):623-31.
13. Ryden L, Sigstrom R, Nilsson J, Sundh V, Erhag HF, Kern S, Waern M, Ostling S, Wilhelmson K, Skoog I. Agreement between self-reports, proxy-reports and the National Patient Register regarding diagnoses of cardiovascular disorders and diabetes mellitus in a population-based sample of 80-year-olds. Age Ageing. 2019;48(4):513-8.
14. Lagergren M, Fratiglioni L, Hallberg IR, Berglund J, Elmstahl S, Hagberg B, Holst G, Rennemark M, Sjolund BM, Thorslund M, Wiberg I, Winblad B, Wimo A. A longitudinal study integrating population, care and social services data. The Swedish National study on Aging and Care (SNAC). Aging Clin Exp Res. 2004;16(2):158-68.
15. Lissner L, Skoog I, Andersson K, Beckman N, Sundh V, Waern M, Zylberstein DE, Bengtsson C, Bjorkelund C. Participation bias in longitudinal studies: experience from the Population Study of Women in Gothenburg, Sweden. Scand J Prim Health Care. 2003;21(4):242-7.
16. Bergholdt HKM, Bathum L, Kvetny J, Rasmussen DB, Moldow B, Hoeg T, Jemec GBE, Berner-Nielsen H, Nordestgaard BG, Ellervik C. Study design, participation and characteristics of the danish general suburban population study. Dan Med J. 2013;Sep;60(9):A469.
17. Chatfield MD, Brayne CE, Matthews FE. A systematic literature review of attrition between waves in longitudinal studies in the elderly shows a consistent pattern of dropout between differing studies. J Clin Epidemiol. 2005;58(1):13-9.
18. Matthews FE, Chatfield M, Brayne C, Cfas M. An investigation of whether factors associated with short-term attrition change or persist over ten years: data from the Medical Research Council Cognitive Function and Ageing Study (MRC CFAS). BMC Public Health. 2006;6.
19. Reinikainen J, Tolonen H, Borodulin K, Harkanen T, Jousilahti P, Karvanen J, Koskinen S, Kuulasmaa K, Mannisto S, Rissanen H, Vartiainen E. Participation rates by educational levels have diverged during 25 years in Finnish health examination surveys. Eur J Public Health. 2018;28(2):237-43.
20. Lundberg I, Damström Thakker K, Hällström T, Forsell Y. Determinants of non-participation, and the effects of non-participation on potential cause-effect relationships, in the PART study on mental disorders. Soc Psychiatry Psychiatr Epidemiol. 2005;40(6):475-83.
21. Bergstrand R, Vedin A, Wilhelmsson C, Wilhelmsen L. Bias Due to Non-Participation and Heterogenous Subgroups in Population Surveys. J Chronic Dis. 1983;36(10):725-8.
22. Korkeila K, Suominen S, Ahvenainen J, Ojanlatva A, Rautava P, Helenius H, Koskenvuo M. Non-response and related factors in a nation-wide health survey. Eur J Epidemiol. 2001;17(11):991-9.
23. Knudsen AK, Hotopf M, Skogen JC, Overland S, Mykletun A. The Health Status of Nonparticipants in a Population-based Health Study The Hordaland Health Study. Am J Epidemiol. 2010;172(11):1306-14.
24. Turkiewicz A, Gerhardsson de Verdier M, Engström G, Nilsson PM, Mellström C, Lohmander LS, Englund M. Prevalence of knee pain and knee OA in southern Sweden and the proportion that seeks medical care. Rheumatology. 2014;54(5):827-35.
25. Krokstad S, Langhammer A, Hveem K, Holmen TL, Midthjell K, Stene TR, Bratberg G, Heggland J, Holmen J. Cohort Profile: the HUNT Study, Norway. Int J Epidemiol. 2013;42(4):968-77.
26. Laaksonen M, Aittomäki A, Lallukka T, Rahkonen O, Saastamoinen P, Silventoinen K, Lahelma E. Register-based study among employees showed small nonparticipation bias in health surveys and check-ups. J Clin Epidemiol. 2008;61(9):900-6.
27. Härkänen T, Karvanen J, Tolonen H, Lehtonen R, Djerf K, Juntunen T, Koskinen S. Systematic handling of missing data in complex study designs – experiences from the Health 2000 and 2011 Surveys. Journal of Applied Statistics. 2016;43(15):2772-90.

For peer review only

Figure captions

Fig. 1 Sample flow chart describing the five cross-sectional examination waves

Figure legend

Rr = Response rate (participants/ eligible sample)
[1]All 70-year-olds living in Gothenburg and born in 1930 on days 3, 6, 12, 18, 21, 24, or 30
[2]All 75-year-olds living in Gothenburg and born in 1930 on days 2, 3, 5, 6, 11, 12, 16, 18, 20, 21, 24, 27 (in January-May), or 30
[3]n=386 had previously been examined at age 70.
[4]Included only individuals that had been invited to the 2000 or 2005 examination
[5]Misclassified as not living in Gothenburg and therefore not invited
[6] n=539 had previously been examined (n=307 at age 70, and n=511 at age 75, and n=279 in both)
[7]All 85-year-olds living in Gothenburg and born in 1930 on days 2, 3, 5, 6, 11, 12, 16, 18, 20, 21, 24, 27, or 30
[8]Misclassified as not living in Gothenburg and therefore not invited
[9]n=370 had previously been examined (n=199 at age 70, n=338 at age 75, n=337 at age 79, and n=174 in all three)
[10]All 88-year-olds living in Gothenburg and born in 1930 on days 2, 3, 5, 6, 11, 12, 16, 18, 20, 21, 24, 27, or 30
11n=247 had previously been examined (n=120 at age 70, n=205 at age 75, n=207 at age 79, n=224 at age 85, and n=102 in all four)

Fig. 2 Mortality graphs in participants and refusals, derived from Cox regression analysis, adjusted by sex.

Figure legend

HR: Hazard ratio refusals compared to participants, CI: confidence interval.

From age 75, Schoenfeld residuals indicated deviation from the assumption of proportional hazards ($p<0.001$), showing that the higher mortality among refusals was limited to the first 2 years, which is why a graph was not plotted. The 0–2-year mortality HR: 4.8, 95% CI: 2.7-8.6, and 3-5 year mortality HR: 1.3, 95% CI: 0.9-1.9.

a) 2000: 5-year mortality from age 70, HR: 3.7, 95% CI: 2.1-6.3, Schoenfeld residuals p=0.94.

b) 2009: 5-year mortality from age 79, HR: 1.6, 95% CI: 1.2-2.1, Schoenfeld residuals p=0.29.

c) 2015: 5-year mortality from age 85, HR: 1.6, 95% CI: 1.2-2.1, Schoenfeld residuals p=1.00.

d) 2018: 2-year mortality from age 88, HR: 2.8, 95% CI: 1.7-4.5, Schoenfeld residuals p=0.60.

For peer review only - http://bmjopen.bmj.com/site/about/guidelines.xhtml

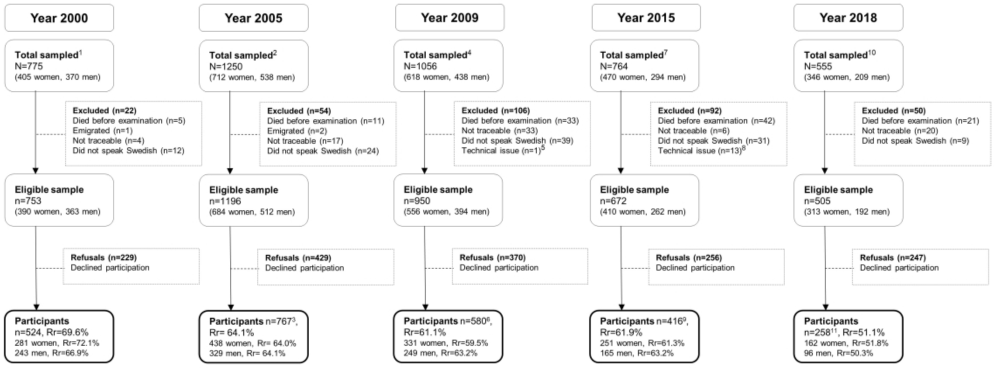

Figure captions.

Fig. 1 Sample flow chart describing the five cross-sectional examination waves.

Figure legend.

Rr = Response rate (participants/ eligble sample).

1 All 70-year-olds living in Gothenburg and born in 1930 on days 3, 6, 12, 18, 21, 24, or 30.

2 All 75-year-olds living in Gothenburg and born in 1930 on days 2, 3, 5, 6, 11, 12, 16, 18, 20, 21, 24, 27 (in January-May), or 30.

3 n=386 had previously been examined at age 70.

4 Included only individuals that had been invited to the 2000 or 2005 examination.

5 Misclassified as not living in Gothenburg and therefore not invited.

6 n=539 had previously been examined (n=307 at age 70, and n=511 at age 75, and n=279 in both).

7 All 85-year-olds living in Gothenburg and born in 1930 on days 2, 3, 5, 6, 11, 12, 16, 18, 20, 21, 24, 27, or 30.

8 Misclassified as not living in Gothenburg and therefore not invited.

9 n=370 had previously been examined (n=199 at age 70, n=338 at age 75, n=337 at age 79, and n=174 in all three).

10 All 88-year-olds living in Gothenburg and born in 1930 on days 2, 3, 5, 6, 11, 12, 16, 18, 20, 21, 24, 27, or 30.

11 n=247 had previously been examined (n=120 at age 70, n=205 at age 75, n=207 at age 79, n=224 at age 85, and n=102 in all four).

338x190mm (300 x 300 DPI)

For peer review only

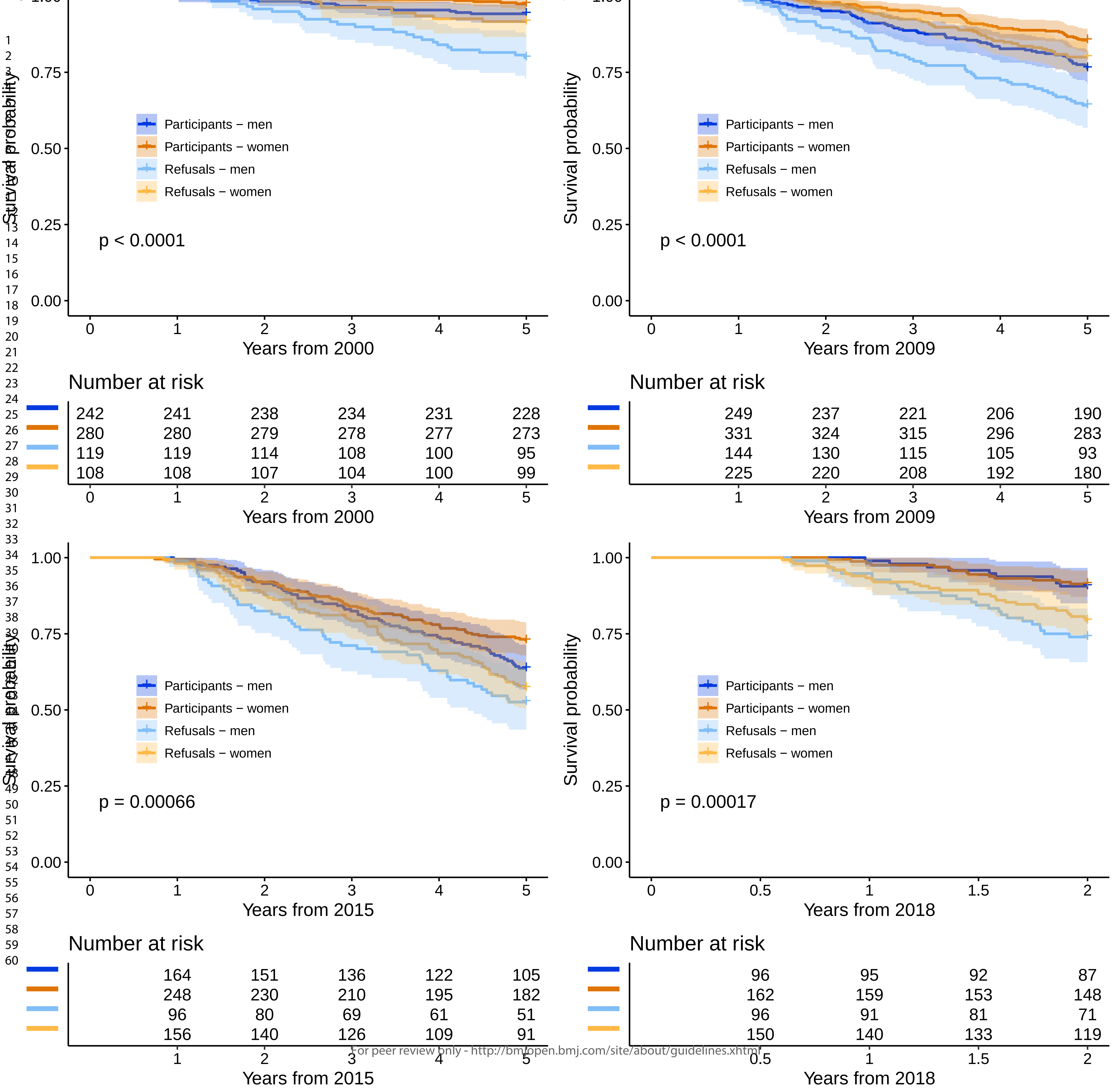
Survival probability
1.00
0.75
0.50
0.25
0.00
Participants − men
Participants − women
Refusals − men
Refusals − women
p < 0.0001
Years from 2000
Number at risk
242 241 238 234 231 228
280 280 279 278 277 273
119 119 114 108 100 95
108 108 107 104 100 99
Survival probability
p < 0.0001
Years from 2009
Number at risk
249 237 221 206 190
331 324 315 296 283
144 130 115 105 93
225 220 208 192 180
Survival probability
p = 0.00066
Years from 2015
Number at risk
164 151 136 122 105
248 230 210 195 182
96 80 69 61 51
156 140 126 109 91
Survival probability
p = 0.00017
Years from 2018
Number at risk
96 95 92 87
162 159 153 148
96 91 81 71
150 140 133 119

Additional file 1

BMJ Open

Representativeness in population-based studies of older adults – Five waves of cross-sectional examinations in the Gothenburg H70 Birth Cohort Study

Hanna Wetterberg, MSc[ab]*, Lina Rydén, MD[abc]*, Felicia Ahlner, MSc[ab], Hanna Falk Erhag, RN, PhD[ab], Pia Gudmundsson, PhD[ab], Xinxin Guo, MD, PhD[ab],[d], Erik Joas, PhD[ab], Lena Johansson, RN, PhD[ab], Silke Kern, MD, PhD[abc], Madeleine Mellqvist Fässberg, PhD[ab], Jenna Najar, MD, PhD[abc], Mats Ribbe, MD, PhD[ab], Therese Rydberg Sterner, PhD[abe], Jessica Samuelsson, MSc[ab], Simona Sacuiu MD, PhD[abc], Robert Sigström, MD, PhD[abc], Johan Skoog, PhD[ab], Margda Waern, MD, PhD[abf], Anna Zettergren, PhD[ab], Ingmar Skoog, MD, PhD[abc]

[*] HW and LR are joint first authors

[a]Neuropsychiatric Epidemiology Unit, Department of Psychiatry and Neurochemistry, Institute of Neuroscience and Physiology, Sahlgrenska Academy, at the University of Gothenburg, Sweden; [b]Centre for Ageing and Health (AGECAP) at the University of Gothenburg, Sweden; [c]Region Västra Götaland, Sahlgrenska University Hospital, Psychiatry, Cognition and Old Age Psychiatry Clinic, Gothenburg, Sweden; [d]Region Västra Götaland, Sahlgrenska University Hospital, Department of Mood disorders, Gothenburg, Sweden; [e]Aging Research Center, Department of Neurobiology, Care Sciences and Society, Karolinska Institutet and Stockholm University, Stockholm, Sweden; [f]Region Västra Götaland, Department of Psychiatry, Psychotic Disorders, Sahlgrenska University Hospital, Mölndal, Sweden

Corresponding author:

Hanna Wetterberg, hanna.wetterberg@gu.se

For peer review only

Table s1. Examinations included at each wave

	2000	2005	2009	2015	2018
General examinations					
General health interview	YES	YES	YES	YES	YES
Psychiatric interview	YES	YES	YES	YES	YES
Cognitive examination[a]	YES [g]	YES	YES	YES	YES
Physical examination by medical doctor	YES	YES	NO	NO	NO
Physical examination[b]	YES	YES	YES	YES	YES
Social factors interview	YES	YES	YES	YES	YES
Physiotherapy examination	YES	YES	YES	YES	YES
Functional ability[d]	YES	YES	YES	YES	YES
Periodic limb movement	YES[h]	YES	NO	NO	NO
Audiological examination	YES	YES	NO	YES[i]	NO
Dental examination	YES	YES	NO	NO	NO
Blood sampling[e]	YES	YES	YES	YES	YES
Genetic analyses	YES	YES	YES	YES	YES
Urine sampling	YES	YES	NO	NO	NO
Personality tests (Cesarec Marke Personality Scheme and Eysenck)	NO	YES	YES	YES	NO
Additional examinations					
Close informant interview	YES	YES	YES	YES	YES
Dietary examination	YES	NO	NO	YES	NO
Body composition examination					
Dual-energy X-ray absorptiometry	NO	YES[h]	YES[h]	YES[h]	YES[h]
Bioelectrical impedance spectroscopy	NO	YES[h]	YES[h]	YES[h]	YES[h]
Brain imaging examination					
Magnetic resonance imaging	NO	NO	NO	YES	YES
Computed tomography	YES	NO	YES	YES	YES
Cerebrospinal fluid sampling[f]	NO	NO	YES	YES	YES
Extended audiological examination	NO	NO	NO	YES[h]	YES[h]

[a] Including clinical screening tests as well as more extensive psychometric tests
[b] Including examinations such as blood pressure, anthropometry, visual acuity, neurological status, electrocardiogram, lung function examinations, Bioelectrical Impedance Analysis,
[d] Measured as Activities of Daily Living (ADL), and instrumental activities of daily living (IADL) similar to Katz and Lawton ADL, and from 2015 also the Barthel index.
[e] Used for e.g. clinical chemistry analyses.
[f] Collected by lumbar puncture.
[g] Half of the sample performed an extended psychometric test, which in later cohorts were performed by all participants.
[h] A subsample was invited.
[i] Performed on those examined at the research clinic and a subsample of those examined at home.

For peer review only

Table s2. The hospital discharge diagnoses included

	ICD 9	ICD 10
Cancer	140-208	C00-C97
Neuropsychiatric disorders including dementia disorders	290-319, 324-359<br>290, 291C, 294, 331	F01-F99, G06-G98<br>F01, F02, G30, G31]
Cardiovascular diseases	390-459	I00-I99
Diabetes mellitus	250	E10-E14
Unipolar depression	296B, 311	F32-F33
Alcohol-related disorders	291, 303, 305A	F10
Ischemic heart disease	410-414	I20-I25
Cerebrovascular diseases	430-438	I60-I69
Chronic obstructive pulmonary disease	490-492, 495-496	J40-J44
Osteoarthritis	715	M15-M19

For peer review only

Table s3. Comparison between examinations of excluded individuals

Total excluded (includes did not speak Swedish, died after sampling, and not traceable)

	**2000** 2.8% (22/775)	**2005** 4.3% (54/1250)	**2009** 10.0% (106/1056)	**2015** 12.0% (92/764)	**2018** 9.0% (50/555)
**2000**, 2.8% (22/775)	-	p=0.088	**p<0.001**	**p<0.001**	**p<0.001**
**2005**, 4.3% (54/1250)		-	**p<0.001**	**p<0.001**	**p<0.001**
**2009**, 10.0% (106/1056)			-	p=0.175	p=0.507
**2015**, 12.0% (92/764)				-	p=0.079
**2018**, 9.0% (50/555)					-

Did not speak Swedish

	**2000** 1.6% (12/775)	**2005** 1.9% (24/1250)	**2009** 3.7% (39/1056)	**2015** 4.1% (31/764)	**2018** 1.6% (9/555)
**2000,**1.6% (12/775)	-	p=0.538	**p=0.006**	**p=0.003**	p=0.916
**2005,** 1.9% (24/1250)		-	**p=0.009**	**p=0.001**	p=0.867
**2009,** 3.7% (39/1056)			-	p=0.690	**p=0.020**
**2015,** 4.1% (31/764)				-	**p=0.011**
**2018,** 1.6% (9/555)					-

Died after sampling

	**2000** 0.7% (5/775)	**2005** 0.9% (11/1250)	**2009** 3.1% (33/1056)	**2015** 5.5% (42/764)	**2018** 3.8% (21/555)
**2000,** 0.7% (5/775)	-	p=0.562	**p<0.001**	**p<0.001**	**p<0.001**
**2005,** 0.9% (11/1250)		-	**p<0.001**	**p<0.001**	**p=0.017**
**2009,** 3.1% (33/1056)			-	**p=0.012**	p=0.485
**2015,** 5.5% (42/764)				-	p=0.150
**2018,** 3.8% (21/555)					-

Not traceable

	**2000** 0.5% (4/775)	**2005** 1.4% (17/1250)	**2009** 3.1% (33/1056)	**2015** 0.8% (6/764)	**2018** 3.6% (20/555)
**2000**, 0.5% (4/775)	-	p=0.074	**p<0.001**	p=0.544	**p<0.001**
**2005**, 1.4% (17/1250)		-	**p=0.004**	p=0.324	**p<0.001**
**2009**, 3.1% (33/1056)			-	**p=0.001**	p=0.609
**2015**, 0.8% (6/764)				-	**p<0.001**
**2018**, 3.6% (20/555)					-

Statistical analysis: to test differences between groups χ2 or Fisher's exact test were used. Bolded numbers are significant at p <0.05.

For peer review only

Table s4. Characteristics of 70 years old participants and refusals, and same aged individuals in Gothenburg and Sweden in 2000, stratified by sex

	**Women**				**Men**			
	**Participants**	**Refusals**	**Gothenburg**	**Sweden**	**Participants**	**Refusals**	**Gothenburg**	**Sweden**
N	281 (53.6)	109 (47.6)	1 965 (56.0)	40 655 (53.9)	243[a] (46.4)	120 (52.4)	1 543 (44.0)	34 813 (46.1)
**Sociodemographic factors**								
Marital status, % (n)								
Married	46.3 (130)	49.5 (54)	49.0 (963)	**55.2 (22 425)****	74.8 (181)	**56.7 (68)*****	**65.1 (1 005)****	69.7 (24 272)
Widowed	24.9 (70)	22.0 (24)	23.2 (455)	24.4 (9 937)	2.9 (7)	**8.3 (10)***	**6.4 (98)***	**6.8 (2 354)***
Divorced	23.1 (65)	19.3 (21)	21.3 (419)	**14.5 (5 899)*****	14.9 (36)	15.0 (18)	16.9 (260)	13.4 (4 677)
Never married	5.7 (16)	9.2 (10)	6.5 (128)	5.9 (2 394)	7.4 (18)	**20.0 (24)*****	11.7 (180)	10.1 (3 510)
Education, % (n)								
Elementary school	54.4 (153)	58.7 (64)	51.5 (1 012)	55.3 (22 497)	41.0 (*100*)	**58.0 (*69*)****	43.2 (666)	**52.3 (18 197)*****
Upper secondary school	31.7 (89)	22.0 (24)	30.1 (591)	29.9 (12 150)	36.4 (88)	32.5 (39)	36.2 (558)	31.2 (10 877)
Higher education	12.1 (34)	15.6 (17)	14.0 (276)	12.5 (5 088)	21.5 (52)	**7.5 (9)*****	17.4 (269)	**14.9 (5 203)****
Born in Sweden, % (n)	83.6 (235)	75.2 (82)	80.0 (1 572)	86.2 (35 049)	83.9 (203)	77.5 (93)	80.9 (1 249)	**89.3 (31 075)****
Income, mean (SD)	8970 (5098)	8270 (6637)	8670 (5190)	8520 (7320)	14370 (10824)	**11980 (8646)***	**12270 (1403)****	**12770 (43821)***
Paid labour, % (n)	3.6 (10)	3.0 (*3*)	2.8 (55)	2.2 (875)	7.0 (17)	5.8 (7)	5.4 (83)	5.7 (1 975)
**Hospital discharge diagnoses,** % (n)								
Cancer	6.8 (19)	9.2 (10)	9.3 (182)	7.6 (3087)	8.2 (20)	10.0 (12)	8.6 (132)	6.9 (2401)
Neuropsychiatric diseases	5.7 (16)	7.3 (8)	9.0 (176)	7.8 (3161)	6.6 (16)	**20.8 (25)*****	**11.5 (177)***	9.2 (3196)
Dementia	1.1 (3)	1.8 (2)	0.6 (11)	0.5 (190)	0.4 (1)	**4.2 (5)***	0.6 (9)	0.5 (167)
Cardiovascular diseases	18.1 (51)	**29.4 (32)***	21.0 (412)	21.3 (8654)	30.0 (73)	40.0 (48)	33.4 (515)	32.9 (11455)
Diabetes mellitus	2.8 (8)	3.7 (4)	3.5 (69)	3.9 (1580)	6.2 (15)	5.8 (7)	6.6 (102)	6.1 (2105)
Unipolar depression	1.1 (3)	0 (0)	1.8 (36)	1.6 (659)	0.4 (1)	0 (0)	1.2 (18)	1.2 (400)
Alcohol-related disorders	0.7 (2)	1.8 (2)	0.8 (15)	0.6 (240)	2.5 (6)	**8.3 (10)***	3.2 (50)	2.3 (816)
Ischemic heart diseases	6.4 (18)	7.3 (8)	7.0 (137)	7.2 (2929)	14.8 (36)	17.5 (21)	17.2 (266)	16.4 (5712)
Cerebrovascular diseases	3.9 (11)	4.6 (5)	4.2 (83)	4.4 (1782)	1.2 (3)	**11.7 (14)*****	**7.0 (108)*****	**7.6 (2640)*****
chronic obstructive pulmonary disease	2.5 (7)	**8.3 (9)****	3.4 (66)	2.0 (808)	2.1 (5)	**6.7 (8)***	3.2 (49)	2.3 (811)

For peer review only

Osteoarthritis	7.8 (22)	2.8 (3)	5.3 (104)	6.1 (2479)	4.5 (11)	1.7 (2)	4.0 (61)	5.5 (1905)

Comparisons made between participants and refusals, Gothenburg and Sweden.
Statistical analysis: to test differences between groups t test or χ2 test were used. *** p <0.001, ** p <0.01, * p <0.05; bolded numbers are significant at p <0.05; all other values are not significant.
[a] One individual is missing in the sociodemographic variables due personal identity number not found in the register.
Numbers in italics are not exact (true value within a 1-3 range) due to protection of the anonymity.

For peer review only

Table s5. Characteristics of 75 years old participants and refusals, and same aged individuals in Gothenburg and Sweden in 2005, stratified by sex

	**Women**				**Men**			
	**Participants**	**Refusals**	**Gothenburg**	**Sweden**	**Participants**	**Refusals**	**Gothenburg**	**Sweden**
N (%)	438 (57.1)	246 (57.3)	1 768 (57.5)	37 075 (55.5)	329 [a] (42.9)	183 (42.7)	1 307 (42.5)	29 780 (44.5)
**Sociodemographic factors**								
Marital status, % (n)								
Married	41.8 (183)	41.9 (103)	42.9 (759)	46.2 (17 134)	71.9 (235)	**55.7 (102)***	**65.2 (852)***	67.5 (20 104)
Widowed	33.1 (145)	26.8 (66)	30.3 (535)	33.9 (12 581)	8.0 (26)	**14.8 (27)***	9.6 (126)	10.5 (3 134)
Divorced	20.1 (88)	25.6 (63)	20.7 (366)	**14.2 (5 282)***	13.1 (43)	16.4 (30)	14.8 (194)	12.7 (3 769)
Never married	5.0 (22)	5.7 (14)	6.1 (108)	5.6 (2 078)	7.0 (23)	**13.1 (24)***	10.3 (135)	9.3 (2 773)
Education, % (n)								
Elementary school	49.0 (*213*)	**59.3 (146)****	51.5 (910)	**54.7 (20 280)***	38.8 (127)	**57.9 (106)***	42.6 (557)	**51.4 (15 304)***
Upper secondary school	35.8 (157)	**23.6 (58)***	**30.3 (535)***	**30.2 (11 190)***	37.0 (121)	**26.2 (48)***	36.0 (470)	**31.4 (9 363)***
Higher education	15.1 (66)	13.0 (32)	14.1 (250)	12.9 (4 772)	22.9 (75)	**12.6 (23)****	18.6 (243)	**15.6 (4 636)***
Born in Sweden, % (n)	83.6 (366)	80.1 (197)	80.4 (1 421)	86.4 (32 025)	81.7 (267)	80.3 (147)	80.6 (1 053)	**89.4 (26 615)***
Income, mean (SD)	10240 (8273)	9920 (7564)	10010 (7157)	9650 (8295)	13680 (7477)	12760 (7789)	13630 (10275)	13410 (24038)
Paid labour, % (n)	1.6 (7)	1.0 (2)	1.8 (31)	1.1 (409)	3.7 (12)	2.7 (5)	3.4 (44)	3.4 (1 016)
**Hospital discharge diagnoses, %** (n)								
Cancer	12.1 (53)	11.0 (27)	12.7 (225)	10.4 (3872)	14.6 (48)	14.2 (26)	13.2 (172)	**10.8 (3216)***
Neuropsychiatric diseases	12.3 (54)	14.2 (35)	12.3 (218)	11.2 (4157)	10.6 (35)	**18.6 (34)***	**16.4 (214)****	13.1 (3913)
Dementia	2.1 (9)	2.0 (5)	1.8 (32)	**0.9 (340)***	0.6 (2)	1.6 (3)	1.0 (13)	0.9 (264)
Cardiovascular diseases	32.9 (144)	35.4 (87)	33.5 (592)	32.5 (12042)	42.2 (139)	**53.0 (97)***	47.4 (619)	45.6 (13576)
Diabetes mellitus	8.0 (35)	8.5 (21)	6.3 (111)	6.0 (2238)	9.7 (32)	10.9 (20)	10.6 (139)	8.8 (2634)
Unipolar depression	1.4 (6)	**3.7 (9)***	2.8 (49)	2.4 (883)	1.8 (6)	1.6 (3)	2.5 (33)	1.6 (489)
Alcohol-related disorders	1.1 (5)	0.8 (2)	0.7 (13)	0.6 (230)	1.5 (5)	3.3 (6)	2.9 (38)	2.3 (676)
Ischemic heart diseases	9.6 (42)	11.0 (27)	11.1 (196)	11.1 (4103)	22.5 (74)	23.5 (43)	24.2 (316)	22.7 (6773)

For peer review only

Cerebrovascular diseases	6.4 (28)	6.5 (16)	6.4 (113)	7.1 (2635)	10.6 (35)	10.9 (20)	11.6 (151)	11.5 (3419)
chronic obstructive pulmonary disease	4.3 (19)	6.5 (16)	3.9 (69)	3.0 (1094)	4.0 (13)	6.6 (12)	5.4 (71)	3.9 (1158)
Osteoarthritis	12.1 (53)	**6.1 (15)***	9.5 (167)	10.3 (3834)	6.1 (20)	3.8 (7)	5.7 (74)	8.9 (2643)

Comparisons made between participants and refusals, Gothenburg and Sweden.
Statistical analysis: to test differences between groups t test or χ2 test were used. *** p <0.001, ** p <0.01, * p <0.05; bolded numbers are significant at p <0.05; all other values are not significant.
[a] One individual is missing in the sociodemographic variables due personal identity number not found in the register.
Numbers in italics are not exact (true value within a 1-3 range) due to protection of the anonymity.

For peer review only

Table s6. Characteristics of 79 years old participants and refusals, and same aged individuals in Gothenburg and Sweden in 2009, stratified by sex

	**Women**				**Men**			
	**Participants**	**Refusals**	**Gothenburg**	**Sweden**	**Participants**	**Refusals**	**Gothenburg**	**Sweden**
N (%)	331 (57.1)	225 (60.8)	1566 (59.2)	33167 (57.0)	249 (42.9)	145 (39.2)	1081 (40.8)	25017 (43.0)
**Sociodemographic factors**								
Marital status, % (n)								
Married	34.1 (113)	37.8 (85)	35.2 (552)	37.7 (12519)	69.9 (174)	**54.5 (79) ****	63.5 (686)	64.8 (16217)
Widowed	40.8 (135)	34.7 (78)	38.4 (602)	43.2 (14325)	12.4 (31)	17.2 (25)	13.5 (146)	14.6 (3648)
Divorced	19.6 (65)	23.1 (52)	20.6 (322)	13.7 (4536)**	9.6 (24)	15.9 (23)	14.0 (151)	12.0 (3014)
Never married	5.4 (18)	4.4 (10)	5.7 (90)	5.4 (1787)	8.0 (20)	12.4 (18)	9.1 (98)	8.5 (2138)
Education, % (n)								
Elementary school	44.0 (*144*)	**63.0 (*142*)*****	**50.1 (784) ***	**54.1 (17955)*****	36.1 (90)	**52.0 (*75*)****	39.0 (422)	**50.4 (12601)*****
Upper secondary school	38.1 (126)	**23.6 (53) *****	**30.6 (479) ****	**30.2 (10033) ****	39.8 (99)	31.7 (46)	37.4 (404)	**31.8 (7953)****
Higher education	17.8 (59)	12.4 (28)	15.2 (238)	**13.3 (4419) ***	24.1 (60)	**14.5 (21)***	20.7 (224)	**16.2 (4056)*****
Born in Sweden, % (n)	83.7 (277)	86.2 (194)	80.4 (1259)	86.4 (28655)	85.1 (212)	86.2 (125)	80.6 (871)	**89.5 (22391)***
Income, mean (SD)	12720 (13490)	**10440 (3880)****	11740 (9393)	**11230 (11699)***	16680 (10368)	14750 (11032)	16150 (15193)	**14780 (19926)****
Paid labour, % (n)	1.0 (*2*)	1.0 (2)	0.6 (10)	0.5 (160)	2.4 (6)	2.0 (*3*)	0.8 (9 [a])*	1.7 (432)
**Hospital discharge diagnoses,** % (n)								
Cancer	16.0 (53)	11.1 (25)	14.7 (230)	12.7 (4217)	15.3 (38)	17.2 (25)	16.5 (178)	14.5 (3634)
Neuropsychiatric diseases	14.2 (47)	16.4 (37)	16.8 (263)	15.6 (5168)	19.3 (48)	13.8 (20)	20.4 (221)	17.7 (4422)
Dementia	2.1 (7)	1.3 (3)	1.8 (28)	1.7 (568)	1.6 (4)	0 (0)	2.0 (22)	1.8 (451)
Cardiovascular diseases	42.9 (142)	37.3 (84)	44.8 (702)	44.9 (14903)	53.0 (132)	53.1 (77)	56.2 (608)	56.5 (14132)
Diabetes mellitus	7.6 (25)	6.2 (14)	8.0 (126)	8.4 (2792)	10.8 (27)	9.0 (13)	11.8 (128)	2903 (11.6)
Unipolar depression	2.7 (9)	4.0 (9)	4.2 (66)	3.3 (1094)	4.4 (11)	2.8 (4)	3.7 (40)	**2.1 (526)***
Alcohol-related disorders	1.2 (4)	0.9 (2)	0.7 (11)	0.6 (189)	0.4 (1)	**5.5 (8)****	**3.1 (34)***	**2.2 (554)***
Ischemic heart diseases	12.4 (41)	9.3 (21)	14.0 (219)	14.5 (4795)	23.7 (59)	29.0 (42)	26.5 (287)	27.2 (6794)
Cerebrovascular diseases	7.6 (25)	6.7 (15)	9.6 (151)	9.8 (3236)	14.9 (37)	11.7 (17)	15.2 (164)	14.9 (3737)
chronic obstructive	3.9 (13)	4.9 (11)	5.4 (85)	4.0 (1342)	6.0 (15)	3.4 (5)	6.1 (66)	5.4 (1340)

For peer review only

For peer review only - http://bmjopen.bmj.com/site/about/guidelines.xhtml

pulmonary disease								
Osteoarthritis	17.2 (57)	**9.3 (21)****	14.4 (226)	14.8 (4919)	11.2 (28)	**3.4 (5)****	9.2 (99)	12.4 (3110)

Comparisons made between participants and refusals, Gothenburg and Sweden.
Statistical analysis: to test differences between groups t test or χ2 test were used. *** p <0.001, ** p <0.01, * p <0.05; bolded numbers are significant at p <0.05; all other values are not significant.
[a] Information is missing on 3 individuals
Numbers in italics are not exact (true value within a 1-3 range) due to protection of the anonymity.

For peer review only

Table s7. Characteristics of 85 years old participants and refusals, and same aged individuals in Gothenburg and Sweden in 2015, stratified by sex

	**Women**				**Men**			
	**Participants**	**Refusals**	**Gothenburg**	**Sweden**	**Participants**	**Refusals**	**Gothenburg**	**Sweden**
N (%)	251[a] (60.3)	159[a] (62.1)	1 132 (62.6)	24 417 (60.6)	165 (39.7)	97[a] (37.9)	676 (37.4)	15 899 (39.4)
**Sociodemographic factors**								
Marital status, % (n)								
Married	22.4 (56)	20.9 (33)	23.1 (262)	23.3 (5700)	61.2 (101)	49.0 (47)	55.9 (378)	57.4 (9124)
Widowed	54.0 (135)	53.8 (85)	52.1 (590)	58.6 (14319)	24.8 (41)	27.1 (26)	25.7 (174)	24.2 (3853)
Divorced	18.8 (47)	22.2 (35)	19.8 (224)	**13.1 (3196)****	7.9 (13)	11.5 (11)	11.7 (79)	11.2 (1776)
Never married	4.8 (12)	3.2 (5)	4.9 (56)	4.9 (1202)	6.1 (10)	12.5 (12)	6.7 (45)	7.2 (1146)
Education, % (n)								
Elementary school	41.0 (*103*)	**58.0 (*92*)*****	46.8 (530)	**52.7 (12865)*****	33.0 (*54*)	**50.0 (*48*)****	38.2 (258)	**48.0 (7634)*****
Upper secondary school	39.6 (99)	**28.5 (45)***	**32.0 (362)***	**30.8 (7516)****	40.6 (67)	32.3 (31)	37.7 (255)	**32.5 (5171)***
Higher education	18.0 (45)	12.0 (19)	17.0 (193)	14.3 (3483)	24.8 (41)	**14.6 (14)***	21.0 (142)	**17.9 (2840)***
Born in Sweden, % (n)	84.0 (210)	82.3 (130)	80.4 (910)	86.8 (21194)	87.3 (144)	85.4 (82)	81.8 (553)	89.6 (14251)
Income, mean (SD)	14130 (10371)	14780 (21934)	14810 (25351)	13750 (20675)	18480 (8103)	15670 (17839)	17530 (15431)	17200 (23336)
Paid labour, % (n)	N/A	N/A	N/A	N/A	N/A	N/A	N/A	N/A
**Hospital discharge diagnoses,** % (n)								
Cancer	17.9 (45)	18.9 (30)	18.8 (213)	16.3 (3990)	23.6 (39)	16.5 (16)	22.3 (151)	20.4 (3246)
Neuropsychiatric diseases	23.1 (58)	23.3 (37)	24.5 (277)	25.3 (6175)	23.6 (39)	20.6 (20)	25.0 (169)	25.6 (4070)
Dementia	<4†	<4†	2.4 (27)	3.6 (877)	<4†	<4†	1.3 (9)	3.4 (536)
Cardiovascular diseases	58.6 (147)	60.4 (96)	60.5 (685)	63.2 (15428)	67.9 (112)	69.1 (67)	68.8 (465)	70.6 (11232)
Diabetes mellitus	6.8 (17)	10.1 (16)	9.0 (102)	**11.5 (2812)***	16.4 (27)	8.2 (8)	14.5 (98)	14.9 (2364)
Unipolar depression	3.6 (9)	<4†	5.8 (66)	4.5 (1109)	6.1 (10)	<4†	4.3 (29)	**2.8 (446)***
Alcohol-related disorders	<4†	<4†	0.5 (6)	0.5 (125)	<4†	<4†	2.2 (15)	1.7 (272)
Ischemic heart diseases	12.0 (30)	11.9 (19)	16.4 (186)	**18.7 (4561)****	27.9 (46)	33.0 (32)	30.9 (209)	32.3 (5137)
Cerebrovascular diseases	10.4 (26)	16.4 (26)	13.3 (150)	14.1 (3440)	16.4 (27)	18.6 (18)	18.0 (122)	19.3 (3063)
chronic obstructive	4.8 (12)	5.0 (8)	4.8 (54)	5.2 (1272)	4.8 (8)	4.1 (4)	7.0 (47)	7.1 (1133)

pulmonary disease								
Osteoarthritis	23.9 (60)	17.0 (27)	21.9 (248)	21.2 (5188)	13.9 (23)	7.2 (7)	12.9 (87)	16.8 (2666)

Comparisons made between participants and refusals, Gothenburg and Sweden.
Statistical analysis: to test differences between groups t test or χ2 test were used. *** p <0.001, ** p <0.01, * p <0.05; bolded numbers are significant at p <0.05; all other values are not significant.
[a] One individual is missing in the sociodemographic variables due personal identity number not found in the register.
[b] Information is missing on 2 individuals
Numbers in italics are not exact (true value within a 1-3 range) due to protection of the anonymity.
N/A Not applicable as the cut of age for paid labour in Statistics Sweden registers is 84.
†Less than four individuals. exact number not presented to protect anonymity. Statistical comparison not performed.

For peer review only

Table s8. Characteristics of 88 years old participants and refusals, and same aged individuals in Gothenburg and Sweden in 2018

	Women				Men			
	Participants	Refusals	Gothenburg	Sweden	Participants	Refusals	Gothenburg	Sweden
N (%)	162 (62.8)	151 (61.1)	850 (64.3)	18589 (63.1)	96 (37.2)	96[a] (38.9)	471 (35.7)	10883 (36.9)
**Sociodemographic factors**								
Marital status, % (n)								
Married	10.5 (17)	15.9 (24)	**17.2 (146)***	**16.6 (3078)***	54.2 (52)	51.6 (49)	53.1 (250)	51.9 (5650)
Widowed	69.1 (112)	61.6 (93)	**59.8 (508)***	66.3 (12325)	34.4 (33)	27.4 (26)	30.4 (143)	31.0 (3375)
Divorced	16.0 (26)	19.9 (30)	18.2 (155)	12.4 (2309)	7.3 (7)	10.5 (10)	10.8 (51)	10.6 (1154)
Never married	4.3 (7)	2.6 (4)	4.8 (41)	4.7 (877)	4.2 (4)	10.5 (10)	5.7 (27)	6.5 (704)
Education, % (n)								
Elementary school	37.7 (61)	**54.3 (82)****	45.6 (388)	**51.8 (9625)*****	37.5 (36)	38.9 (37)	36.1 (170)	46.1 (5017)
Upper secondary school	36.0 (*60*)	29.1 (44)	32.1 (273)	30.9 (5741)	33.0 (*34*)	39.0 (*38*)	38.9 (183)	33.1 (3606)
Higher education	23.5 (38)	**13.2 (20)***	17.9 (152)	**15.0 (2792)****	24.0 (23)	16.8 (16)	21.2 (100)	19.2 (2087)
Born in Sweden, % (n)	82.7 (134)	82.8 (125)	80.0 (680)	87.0 (16165)	84.4 (81)	82.1 (78)	81.5 (384)	89.7 (9765)
Income, mean (SD)	19590 (30923)	16200 (19687)	16200 (22412)	15600 (23116)	18850 (13561)	19800 (20415)	19320 (16731)	18960 (29208)
Paid labour, % (n)	N/A	N/A	N/A	N/A	N/A	N/A	N/A	N/A
**Hospital discharge diagnoses,** % (n)								
Cancer	19.1 (31)	20.5 (31)	19.8 (168)	17.4 (3240)	20.8 (20)	19.8 (19)	22.9 (108)	22.0 (2394)
Neuropsychiatric diseases	24.7 (40)	**40.4 (61)****	28.1 (239)	29.0 (5384)	25.0 (24)	26.0 (25)	27.0 (127)	29.0 (3159)
Dementia	<4†	3.3 (5)	2.1 (18)	4.5 (831)	<4†	5.2 (5)	1.9 (9)	4.2 (458)
Cardiovascular diseases	66.0 (107)	67.5 (102)	66.9 (569)	70.3 (13063)	63.5 (61)	75.0 (72)	72.2 (340)	**75.5 (8214)****
Diabetes mellitus	11.7 (19)	9.3 (14)	9.5 (81)	12.5 (2318)	12.5 (12)	16.7 (16)	14.4 (68)	15.6 (1701)
Unipolar depression	5.6 (9)	6.0 (9)	6.4 (54)	4.7 (870)	6.3 (6)	4.2 (4)	4.0 (19)	2.9 (321)
Alcohol-related disorders	0 (0)	<4†	0.5 (4)	0.4 (74)	0 (0)	<4†	2.1 (10)	1.5 (168)
Ischemic heart diseases	13.6 (22)	13.2 (20)	16.6 (141)	**20.0 (3726)***	25.0 (24)	32.3 (31)	29.9 (141)	33.8 (3679)
Cerebrovascular diseases	14.2 (23)	17.2 (26)	14.8 (126)	15.3 (2850)	15.6 (15)	14.6 (14)	17.6 (83)	20.2 (2194)
chronic obstructive	5.6 (9)	<4†	4.4 (37)	5.5 (1020)	5.2 (5)	<4†	5.9 (28)	7.1 (773)

For peer review only

pulmonary disease								
Osteoarthritis	29.0 (47)	**18.5 (28)***	24.5 (208)	23.1 (4292)	15.6 (15)	10.4 (10)	14.4 (68)	18.2 (1976)

Comparisons made between participants and refusals, Gothenburg and Sweden.
Statistical analysis: to test differences between groups t test or χ2 test were used. *** p <0.001, ** p <0.01, * p <0.05; bolded numbers are significant at p <0.05; all other values are not significant.
Numbers in italics are not exact (true value within a 1-3 range) due to protection of the anonymity.
N/A Not applicable as the cut of age for paid labour in Statistics Sweden registers is 84.
†Less than four individuals. exact number not presented to protect anonymity. Statistical comparison not performed.

For peer review only

Fig s1. Response rates in different subgroups at all examination years

	2000			2005			2009			2015			2018		
	Total	Women	Men	Total	Women	Men	Total	Women	Men	Total	Women	Men	Total	Women	Men
**Response rate**	69.5% (523/752)	72.1% (281/390)	66.9% (242/362)	64.1% (765/1194)	64.0% (438/684)	64.1% (327/510)	61.1% (580/950)	59.5% (331/556)	63.2% (249/394)	62.0% (415/669)	61.3% (250/408)	63.2% (165/261)	51.2% (258/504)	51.8% (162/313)	50.3% (96/191)
**Marital status**, *% (n/N)*															
Married, *ref*	71.8% (311/433)	70.7% (130/184)	72.7% (181/249)	67.1% (418/623)	64.0% (183/286)	69.7% (235/337)	63.6% (287/451)	57.1% (113/198)	68.8% (174/253)	66.2% (157/237)	62.9% (56/89)	68.2% (101/148)	48.6% (69/142)	41.5% (17/41)	51.5% (52/101)
Widowed	69.4% (77/111)	74.5% (70/94)	**41.2%** (7/17)**	64.8% (171/264)	68.7% (145/211)	**49.1%** (26/53)**	61.7% (166/269)	63.4% (135/213)	55.4% (31/56)	61.3% (176/287)	61.4% (135/220)	61.2% (41/67)	54.9% (145/264)	54.6% (112/205)	55.9% (33/59)
Divorced	72.1% (101/140)	75.6% (65/86)	66.7% (36/54)	**58.5%* (131/224)**	58.3% (88/151)	58.9% (43/73)	**54.3%* (89/164)**	55.6% (65/117)	**51.1%* (24/47)**	56.6% (60/106)	57.3% (47/82)	54.2% (13/24)	45.2% (33/73)	46.4% (26/56)	41.2% (7/17)
Unmarried	**50.0%*** (34/68)**	61.5% (16/26)	**42.9%*** (18/42)**	**54.2%* (45/83)**	61.1% (22/36)	**48.9%** (23/47)**	57.6% (38/66)	64.3% (18/28)	**52.6%* (20/38)**	56.4% (22/39)	70.6% (12/17)	**45.5%* (10/22)**	44.0% (11/25)	63.6% (7/11)	28.6% (4/14)
**Education**, *% (n/N)*															
Elementary school ≤9y, *ref*	65.5% (253/386)	70.5% (153/217)	59.2% (100/169)	57.4% (340/592)	59.3% (213/359)	54.5% (127/233)	51.9% (234/451)	50.3% (144/286)	54.5% (90/165)	52.9% (157/297)	52.8% (103/195)	52.9% (54/102)	44.9% (97/216)	42.7% (61/143)	49.3% (36/73)
Upper secondary school	**73.8%* (177/240)**	78.8% (89/113)	69.3% (88/127)	**72.4%* (278/384)**	**73.0%*** (157/215)**	**71.6%*** (121/169)**	**69.4%*** (225/324)**	**70.4%*** (126/179)**	**68.3%* (99/145)**	**68.6%*** (166/242)**	**68.8%** (99/144)**	**68.4%* (67/98)**	53.4% (94/176)	**57.7%* (60/104)**	47.2% (34/72)
Higher education	**76.8%* (86/112)**	66.7% (34/51)	**85.2%*** (52/61)**	**71.9%* (141/196)**	67.3% (66/98)	**76.5%*** (75/98)**	**70.8%*** (119/168)**	**67.8%** (59/87)**	**74.1%** (60/81)**	**72.3%*** (86/119)**	**70.3%* (45/64)**	**74.5%** (41/55)**	**62.9%** (61/97)**	**65.5%** (38/58)**	59.0% (23/39)
**Ethnicity**, *% (n/N)*															
Born in Sweden, *ref*	71.5% (438/613)	74.1% (235/317)	68.6% (203/296)	64.8% (633/977)	65.0% (366/563)	64.5% (267/414)	60.5% (489/808)	58.8% (277/471)	62.9% (212/337)	62.5% (354/566)	61.8% (210/340)	63.7% (144/226)	51.4% (215/418)	51.7% (134/259)	50.9% (81/159)
Not born in Sweden	**61.2%* (85/139)**	63.0% (46/73)	59.1% (39/66)	60.8% (132/217)	59.5% (72/121)	62.5% (60/96)	64.1% (91/142)	63.5% (54/85)	64.9% (37/57)	59.2% (61/103)	58.8% (40/68)	60.0% (21/35)	50.0% (43/86)	51.9% (28/54)	46.9% (15/32)

The colour shading indicate response rate. The more dark colour, the higher response rate in comparison to the other subgroups the same examination year.

Comparisons made between the different subgroups at each examination year in the total groups, among women, and among men.

Statistical analysis: to test differences between groups $\chi 2$ test were used. *** p <0.001, ** p <0.01, * p <0.05; bolded numbers are significant at p <0.05; all other values are not significant.

338x190mm (300 x 300 DPI)

STROBE 2007 (v4) Statement—Checklist of items that should be included in reports of *cohort studies*

Section/Topic	Item #	Recommendation	Reported on page #
**Title and abstract**	1	(*a*) Indicate the study's design with a commonly used term in the title or the abstract	1
		(*b*) Provide in the abstract an informative and balanced summary of what was done and what was found	4
**Introduction**			
Background/rationale	2	Explain the scientific background and rationale for the investigation being reported	6
Objectives	3	State specific objectives, including any prespecified hypotheses	6
**Methods**			
Study design	4	Present key elements of study design early in the paper	6
Setting	5	Describe the setting, locations, and relevant dates, including periods of recruitment, exposure, follow-up, and data collection	6, 9
Participants	6	(*a*) Give the eligibility criteria, and the sources and methods of selection of participants. Describe methods of follow-up	6
		(*b*) For matched studies, give matching criteria and number of exposed and unexposed	N/A
Variables	7	Clearly define all outcomes, exposures, predictors, potential confounders, and effect modifiers. Give diagnostic criteria, if applicable	7-8
Data sources/ measurement	8*	For each variable of interest, give sources of data and details of methods of assessment (measurement). Describe comparability of assessment methods if there is more than one group	*7-8*
Bias	9	Describe any efforts to address potential sources of bias	8
Study size	10	Explain how the study size was arrived at	Figure 1
Quantitative variables	11	Explain how quantitative variables were handled in the analyses. If applicable, describe which groupings were chosen and why	8
Statistical methods	12	(*a*) Describe all statistical methods, including those used to control for confounding	8
		(*b*) Describe any methods used to examine subgroups and interactions	8
		(*c*) Explain how missing data were addressed	N/A
		(*d*) If applicable, explain how loss to follow-up was addressed	N/A
		(*e*) Describe any sensitivity analyses	N/A
**Results**			

For peer review only

Participants	13*	(a) Report numbers of individuals at each stage of study—eg numbers potentially eligible, examined for eligibility, confirmed eligible, included in the study, completing follow-up, and analysed	Figure 1
		(b) Give reasons for non-participation at each stage	Figure 1
		(c) Consider use of a flow diagram	Figure 1
Descriptive data	14*	(a) Give characteristics of study participants (eg demographic, clinical, social) and information on exposures and potential confounders	Table 3
		(b) Indicate number of participants with missing data for each variable of interest	N/A
		(c) Summarise follow-up time (eg, average and total amount)	N/A
Outcome data	15*	Report numbers of outcome events or summary measures over time	Table 2, Table 3
Main results	16	(*a*) Give unadjusted estimates and, if applicable, confounder-adjusted estimates and their precision (eg, 95% confidence interval). Make clear which confounders were adjusted for and why they were included	Table 2, Table 3
		(*b*) Report category boundaries when continuous variables were categorized	N/A
		(*c*) If relevant, consider translating estimates of relative risk into absolute risk for a meaningful time period	N/A
Other analyses	17	Report other analyses done—eg analyses of subgroups and interactions, and sensitivity analyses	N/A
**Discussion**			
Key results	18	Summarise key results with reference to study objectives	12
**Limitations**			
Interpretation	20	Give a cautious overall interpretation of results considering objectives, limitations, multiplicity of analyses, results from similar studies, and other relevant evidence	12
Generalisability	21	Discuss the generalisability (external validity) of the study results	15
**Other information**			
Funding	22	Give the source of funding and the role of the funders for the present study and, if applicable, for the original study on which the present article is based	16

*Give information separately for cases and controls in case-control studies and, if applicable, for exposed and unexposed groups in cohort and cross-sectional studies.

Note: An Explanation and Elaboration article discusses each checklist item and gives methodological background and published examples of transparent reporting. The STROBE checklist is best used in conjunction with this article (freely available on the Web sites of PLoS Medicine at http://www.plosmedicine.org/, Annals of Internal Medicine at http://www.annals.org/, and Epidemiology at http://www.epidem.com/). Information on the STROBE Initiative is available at www.strobe-statement.org.

For peer review only
